# Chiral 2-Aminobenzimidazole as Bifunctional Catalyst in the Asymmetric Electrophilic Amination of Unprotected 3-Substituted Oxindoles

**DOI:** 10.3390/molecules23061374

**Published:** 2018-06-06

**Authors:** Llorenç Benavent, Alejandro Baeza, Megan Freckleton

**Affiliations:** Departamento de Química Orgánica and Instituto de Síntesis Orgánica (ISO), Facultad de Ciencias, Universidad de Alicante, Apdo. 99, E-03080 Alicante, Spain; l.benavent@ua.es (L.B.); mf91@alu.ua.es (M.F.)

**Keywords:** organocatalysis, electrophilic amination, benzimidazoles, asymmetric catalysis, oxindoles

## Abstract

The use of readily available chiral *trans*-cyclohexanediamine-benzimidazole derivatives as bifunctional organocatalysts in the asymmetric electrophilic amination of unprotected 3-substituted oxindoles is presented. Different organocatalysts were evaluated; the most successful one contained a dimethylamino moiety (**5**). With this catalyst under optimized conditions, different oxindoles containing a wide variety of substituents at the 3-position were aminated in good yields and with good to excellent enantioselectivities using di-*tert*-butylazodicarboxylate as the aminating agent. The procedure proved to be also efficient for the amination of 3-substituted benzofuranones, although with moderate results. A bifunctional role of the catalyst, acting as Brønsted base and hydrogen bond donor, is proposed according to the experimental results observed.

## 1. Introduction

Substituted oxindoles containing a chiral center at the C3 position are attractive targets in organic synthesis since they are recognized as drug candidates and also for their use as intermediates in alkaloid synthesis. More particularly, the development of efficient methods to prepare 3-aminooxindoles is of current interest owing to the wide presence of these frameworks in bioactive natural products and pharmaceutically active compounds [1,2,3,4,5]. Among the different strategies developed for the construction of these oxindoles, asymmetric organocatalyzed electrophilic amination has emerged as a powerful tool because it is considered a simple and straightforward methodology [6,7,8,9]. Additional to this, the well-known advantages in the use of organocatalysts must be also taken into account: they are readily available or simple-to-prepare catalysts, easy to handle, and there is no risk of metal contamination in the final product [10,11]. For these reasons, it is not surprising that in the last decade, different studies have successfully described the organocatalytic asymmetric electrophilic 3-amination of oxindoles, giving rise to the desired compounds in high yields and enantioselectivities and with a broad substrate scope [12,13,14]. However, most of the works reported so far require the use of *N*-protected oxindoles, which implies a tedious protection-deprotection sequence.

**Figure 1:**
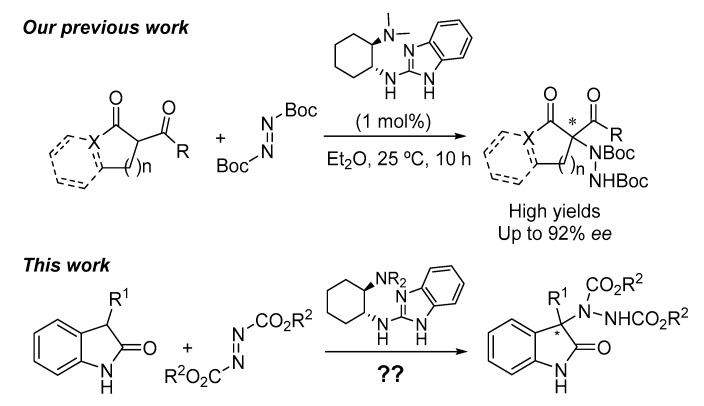
Benzimidazole-based organocatalyst in the asymmetric electrophilic amination of active methylene compounds.

In the last years, our research group has been involved in the synthesis and application of benzimidazole derivatives as bifunctional organocatalysts which activate different active methylene compounds through hydrogen bond interactions in different asymmetric organocatalyzed transformations [15,16,17,18,19]. More concretely, a few years ago we reported the successful employment of such organocatalysts in the enantioselective electrophilic amination of 1,3-dicarbonyl compounds [20,21]. Continuing with this research line, we disclose the application of 2-aminobenzimidazoles as catalysts in the asymmetric electrophilic amination of 3-substituted unprotected oxindoles (Figure 1).

## 2. Results and Discussion

**Figure 2:**
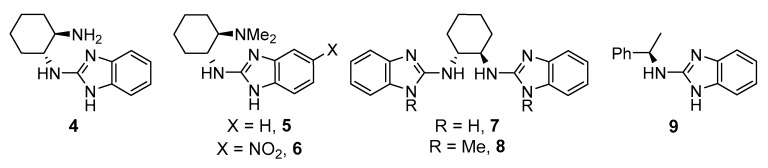
Benzimidazole-derived organocatalysts tested.

Firstly, the search for the optimal conditions was performed by choosing the model reaction between commercial available unprotected 3-methyl-2-oxindole (**1a**) and di-*t*-butylazodicarboxylate (**2a**), using 10 mol % of catalyst loading. Different parameters such as benzimidazole derivative (Figure 2), solvent, and temperature were examined (Table 1). The model reaction was not serendipitously selected because, to the best of our knowledge, only a few articles describe the successful enantioselective amination of such an unprotected oxindole [14,22,23,24]. As observed in the table, among the different benzimidazole derivatives tested (**4**–**9**) at room temperature and using toluene as solvent (Table 1, entries 1–6), catalyst **5** bearing a dimethylamino moiety—which has previously shown its efficiency for the amination of 1,3-dicarbonyl compounds [20]—provided the best results in terms of enantioselectivity (Table 1, entry 2). With the best catalyst at hand, other solvents were evaluated (Table 1, entries 7–11) without observing an improvement in the *ee* of the amination product **3aa**. However, in the case of using hexane as solvent, similar results to those obtained in toluene were achieved (Table 1, entry 10). Therefore, both solvents were tested in a reaction performed at 0 °C for 72 h (Table 1, entries 12 and 13). Whereas the enantioselectivity in toluene increased up to 81% without detriment to conversion, a significant drop in the optical purity of **3aa** was observed in hexane, probably due to the lower solubility of the reagents and catalyst in this solvent at lower temperatures. Attempts to further increase the *ee* of **3aa** by lowering the reaction temperature were unsuccessful, probably for the same solubility reasons (Table 1, entries 14 and 15). It is also important to remark that the concentration barely affects the results observed for the best conditions. However, reducing the catalyst amount (5 mol %) resulted in a dramatic drop of enantioselectivity.

**Scheme 1:**
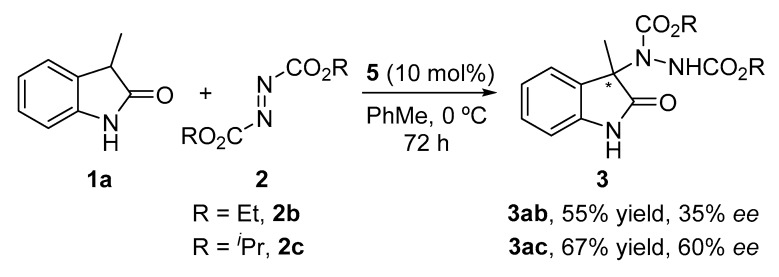
Influence of azodicarboxylate reagent.

Next, we decided to evaluate the influence of the azodicarboxylate reagent. Thus, diethyl (**2b**) and diisopropyl (**2c**) derivatives were allowed to react with oxindole **1a** under the best conditions (Scheme 1). As depicted, none of them improved upon the results obtained with **2a**.

**Scheme 2:**
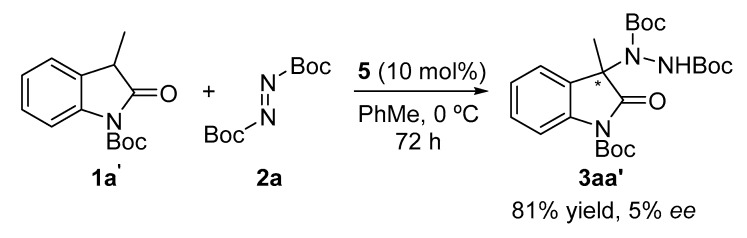
Influence of the *N*-Boc protecting group.

Finally, the last parameter evaluated was the presence of a protecting group on the oxindole. We selected the *Boc* group due to its ease of removal. Surprisingly, the reaction produced the expected amination product in good yields but with low enantioselectivity (Scheme 2).

Once all the parameters which can have an effect in the reaction outcome were studied, the scope of the asymmetric benzimidazole **5** organocatalyzed electrophilic amination was evaluated. As previously mentioned, good yields and enantioselectivities were achieved with 3-methyloxindole (**1a**) (Table 2, entry 1). Similar results were obtained with the 5-bromo analogue **1b** (Table 2, entry 2). Surprisingly, when 3-phenyloxindole (**1c**) was employed, a slight drop in both yield and enantioselectivity was observed (Table 2, entry 3). Better results, especially in term of *ee* values, were obtained with 3-allyloxindole (**3d**) and ester derivative **3e**, reaching 84% and 85% *ee*, respectively (Table 2, entries 4 and 5). Next, a series of 3-benzyl-substituted oxindoles were evaluated. Thus, 3-benzyloxindole (**3e**) was firstly tested giving rise to good yield and enantioselectivity in compound **3fa** (Table 2, entry 6). Surprisingly, excellent enantioselectivity was achieved when a 3-PMB-oxindole (**3g**) was employed (Table 2, entry 7). Encouraged by this result, a couple of *para*-substituted benzyl oxindoles were synthesized and tested (Table 2, entries 8 and 9). As can be observed in the table, the introduction of a NO_2_ group produced the amination product **3ha** with good yields and enantioselectivity. In contrast, we were pleased to discover that the presence of CF_3_ gave rise to the product **3ia** with perfect enantioselectivity.

**Scheme 3:**
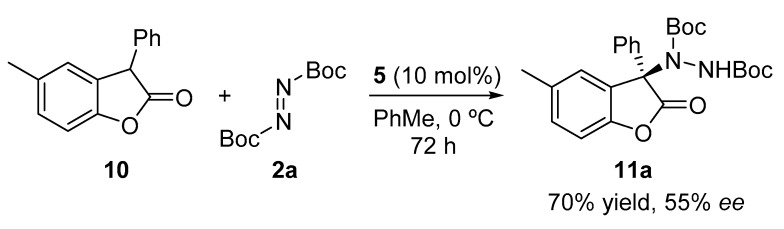
Enantioselective organocatalyzed electrophilic amination of benzofuranone **10**.

After having evaluated the scope of the methodology with different oxindoles and for the sake of comparison, we decided to test 5-methyl-3-phenylbenzofuranone under the optimal reaction conditions (Scheme 3). As depicted, lower enantioselectivity was achieved with this substrate when compared with the oxindole analogue (see Table 2, entry 3).

**Figure 3:**
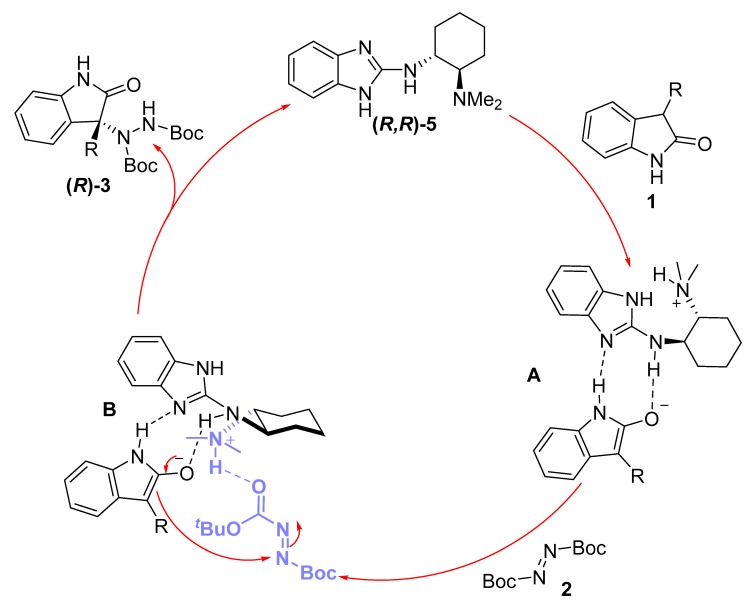
Proposed reaction mechanism.

Concerning a possible reaction mechanism, there is some experimental evidence that needs to be considered before hypothesizing a possible catalytic cycle. Firstly, when comparing some optical rotation values obtained from the amination products **3** with those described previously in the literature, we could conclude that catalyst **5** possessing an (*R,R*)-absolute configuration produced the corresponding (*R*)-products. Secondly, from the results obtained in the catalyst screening, it can be deduced that the dimethylamino moiety is essential for achieving good enantioselectivities, since all the catalysts lacking such a group produced low *ee* values. Finally, the fact that *N*-Boc-protected oxindole gave rise to the amination product in high yields but as racemic mixtures (Scheme 2) led us to think there might be another type of influence besides the possible steric hindrance. With all these data in hand, we hypothesize the following catalytic cycle (Figure 3), assuming the bifunctional role of catalyst **5**: Organocatalyst **5**, acting firstly as a Brønsted base, through the guanidine or dimethylamino moiety, could deprotonate the 3-substituted oxindole. Once the enolate is formed, the guanidine moiety can stablish hydrogen bond interactions not only with the enolate but also with the N–H bond from the amide group of oxindole, as depicted in intermediate **A**. Next, the protonated dimethylamino group can activate the diazocarboxylate by another hydrogen bond interaction (intermediate **B**), forming a close transition state in which the enolate reacts through the *Re*-face, affording the corresponding (*R*)-amination product and releasing the catalyst.

## 3. Materials and Methods 

All reagents were purchased from commercial sources and used without further purification. Substrates which were not commercially available were synthesized according to known literature procedures (see Supporting Information for details). NMR spectra were performed on a Bruker AC-300 or Bruker Avance-400 (Bruker Corporation, Karlsruhe, Germany) using CDCl_3_ as solvent and TMS as internal standard unless otherwise stated. Optical rotations were measured on a Jasco P-1030 Polarimeter with a 5 cm cell (c given in g/100 mL). Enantioselectivities were determined by HPLC analysis (Agilent 1100 Series HPLC) equipped with a G1315B diode array detector and a Quat Pump G1311A (Agilent Technologies, Palo Alto, CA, United States) equipped with the corresponding Daicel chiral column; the retention time of the major enantiomer is highlighted in bold. HRMS were measured using HPLC–HRMS (ESI) equipment (Agilent 1100 Series LC/MSD Trap SL, Agilent Technologies). Analytical TLC was performed on Merck silica gel plates and the spots were visualized with UV light at 254 nm (Merck Millipore, Billerica, MA, USA). Flash chromatography employed Merck silica gel 60 (0.040–0.063 mm) (Merck Millipore, Billerica, MA, USA).

### General Procedure for the Asymmetric Amination of 3-Substituted Oxindoles

In an open-air tube at room temperature (25 °C), the corresponding 3-substituted oxindole (0.10 mmol) was added to a solution of organocatalyst **5** (10 mol %) in toluene (1 mL). After 5 min, the mixture was introduced into a cooling bath at 0 °C and after 2 min, di-*tert*-butylazodicarboxilate (0.11 mmol, 1.1 eq.) was added in one portion. The reaction was then allowed to react for 3 days. After this time, water (5 mL) and ethyl acetate (5 mL) were added, and the aqueous layer was then re-extracted twice (2 × 10 mL). The combined organic phases were dried (MgSO_4_) and evaporated under reduced pressure. Finally, the reaction crude was purified by column chromatography on silica gel or TLC preparative using hexane/ethylacetate mixtures as eluent.

*Di-tert-butyl (R)-1-(3-methyl-2-oxoindolin-3-yl)-hydrazine-1,2-dicarboxylate* (**3aa**) [23]. Colorless oil; [α]_D_^28^ = −7.66 (c = 1.0, CHCl_3_, 81% *ee*). ^1^H-NMR (300 MHz, CDCl_3_) δ*_H_ =* 8.31 (s, 1H), 7.79 (d, *J* = 7.3 Hz, 1H), 7.20 (dd, *J* = 10.9, 4.3 Hz, 1H), 7.06 (t, *J* = 7.5 Hz, 1H), 6.82 (s, 1H), 1.53 (d, *J* = 5.7 Hz, 12H), 1.20 (s, 9H); ^13^C-NMR (75 MHz, CDCl_3_) δ_C_ = 180.35, 156.21, 153.14, 139.34, 133.55, 128.16, 124.05, 123.02, 109.89, 82.54, 81.24, 66.11, 28.25, 27.78, 23.55; chiral HPLC analysis: Chiralcel OD-H column, Hexane/iPrOH 95 : 05, flow rate = 1 mL/min, λ = 240 nm, retention times: 7.2, 8.3 min. 

*Di-tert-butyl 1-(1-(tert-butoxycarbonyl)-3-methyl-2-oxoindolin-3-yl)-hydrazine-1,2-dicarboxylate* (**3aa’**) [25]. Colorless oil; ^1^H NMR (300 MHz, CDCl_3_) ) δ*_H_ =* 7.90 (d, *J* = 7.1 Hz, 1H), 7.80 (d, *J* = 8.0 Hz, 1H), 7.32 (d, *J* = 7.8 Hz, 1H), 7.20 (t, *J* = 7.4 Hz, 1H), 6.65 (s, 1H), 1.65 (s, 9H), 1.53 (d, *J* = 3.4 Hz, 12H), 1.14 (s, 9H); ^13^C-NMR (101 MHz, CDCl_3_) δ_C_ = 176.27, 156.24, 152.87, 149.24, 137.97, 128.44, 124.98, 123.69, 114.65, 84.33, 81.52, 65.81, 28.22, 28.11, 27.66, 24.38; chiral HPLC analysis: Chiralcel IA column, Hexane/iPrOH 90 : 10, flow rate = 1 mL/min, λ = 240 nm, retention times: 12.7, 18.1 min.

*Diethyl 1-(3-methyl-2-oxoindolin-3-yl)-hydrazine-1,2-dicarboxylate* (**3ab**) [23]. Colorless oil; ^1^H-NMR (300 MHz, CDCl_3_) δ*_H_ =* 8.45 (s, 1H), 7.77 (d, *J* = 7.4 Hz, 1H), 7.35 (s, 1H), 7.20 (t, *J* = 7.3 Hz, 1H), 7.05 (t, *J* = 7.5 Hz, 1H), 6.86 (d, *J* = 7.6 Hz, 1H), 4.30 (dd, *J* = 13.5, 6.9 Hz, 2H), 4.00 (d, *J* = 6.8 Hz, 2H), 1.55 (s, 3H), 1.34 (t, *J* = 7.1 Hz, 3H), 1.04 (s, 3H); ^13^C-NMR (101 MHz, CDCl_3_) δ_C_ = 179.59, 157.09, 154.20, 139.39, 132.56, 128.51, 124.18, 123.14, 109.84, 66.27, 62.83, 62.28, 22.95, 14.52, 13.98; chiral HPLC analysis: Chiralcel AD-H column, Hexane/iPrOH 90 : 10, flow rate = 1 mL/min, λ = 240 nm, retention times: 19.3, 33.2 min.

*Diisopropyl (R)-1-(3-methyl-2-oxoindolin-3-yl)-hydrazine-1,2-dicarboxylate* (**3ac**) [23]. Colorless oil; [α]_D_^28^ = −3.64 (c = 1.0, CHCl_3_, 60% *ee*); ^1^H-NMR (300 MHz, CDCl_3_) δ*_H_ =* 8.44 (s, 1H), 7.78 (d, *J* = 7.4 Hz, 1H), 7.20 (t, *J* = 7.3 Hz, 1H), 7.06 (dd, *J* = 13.4, 5.8 Hz, 2H), 6.86 (d, *J* = 7.6 Hz, 1H), 5.05 (dt, *J* = 12.4, 6.2 Hz, 1H), 4.73 (dt, *J* = 12.1, 6.0 Hz, 1H), 1.54 (s, 3H), 1.33 (dd, *J* = 6.2, 1.6 Hz, 6H), 1.08 (d, *J* = 6.1 Hz, 3H), 0.89 (bs, 3H); ^13^C-NMR (101 MHz, CDCl_3_) δ_C_ = 179.69, 156.88, 153.76, 139.38, 132.93, 128.42, 124.19, 123.09, 109.73, 70.81, 70.08, 66.12, 23.09, 22.04, 21.91, 21.65, 21.48; chiral HPLC analysis: Chiralcel AD-H column, Hexane/iPrOH 90 : 10, flow rate = 1 mL/min, λ = 240 nm, retention times: 14.2, 18.7 min.

*Di-tert-butyl (R)-1-(5-bromo-3-methyl-2-oxoindolin-3-yl)-hydrazine-1,2-dicarboxylate* (**3ba**) [24]. Yellow solid; [α]_D_^28^ = +18.64 (c = 1.0, CHCl_3_, 80% *ee*); ^1^H-NMR (300 MHz, CDCl_3_) δ*_H_* = 8.80 (s, 1H), 7.93 (s, 1H), 7.32 (dd, *J* = 8.1, 1.8 Hz, 2H), 6.96–6.68 (m, 2H), 1.54 (m, 12H), 1.25 (s, 9H); ^13^C-NMR (101 MHz, CDCl_3_) δ_C_ = 178.15, 155.91, 152.78, 138.07, 131.16, 127.33, 124.07, 111.19, 82.77, 81.46, 66.89, 28.24, 27.87, 14.12; chiral HPLC analysis: Chiralcel OD-H column, Hexane/iPrOH 95 : 05, flow rate = 0.8 mL/min, λ = 240 nm, retention times: 10.6, 12.4 min.

*Di-tert-butyl (R)-1-(2-oxo-3-phenylindolin-3-yl)-hydrazine-1,2-dicarboxylate* (**3ca**) [24]. White solid; [α]_D_^28^ = −60.38 (c = 1.0, CHCl_3_, 70% *ee*); ^1^H NMR (300 MHz, CDCl_3_): δ*_H_ =* 8.25 (s, 1H), 8.09 (d, *J* = 7.3 Hz, 1H), 7.65–7.57 (m, 2H), 7.30–7.27 (m, 3H), 7.25 (s, 1H), 7.15 (t, *J* = 7.4 Hz, 1H), 6.82 (d, *J* = 7.6 Hz, 1H), 6.26 (bs, 1H), 1.32 (s, 9H), 1.21 (s, 9H); ^13^C-NMR (101 MHz, CDCl_3_) δ_C_ = 177.89, 154.81, 153.48, 140.03, 133.71, 130.59, 129.43, 128.65, 128.59, 128.15, 126.94, 122.64, 109.90, 82.76, 80.83, 72.64, 28.04, 27.81; chiral HPLC analysis: Chiralcel OD-H column, Hexane/iPrOH 90 : 10, flow rate = 1 mL/min, λ = 240 nm, retention times: 5.9, 9.0 min.

*Di-tert-butyl 1-(3-allyl-2-oxoindolin-3-yl)-hydrazine-1,2-dicarboxylate* (**3da**). Yellow oil; [α]_D_^28^ = −11.47 (c = 1.0, CHCl_3_, 84% *ee*); ^1^H-NMR (300 MHz, CDCl_3_) δ*_H_* = 7.93 (s, 1H), 7.76 (d, *J* = 7.4 Hz, 1H), 7.20 (t, *J* = 7.6 Hz, 1H), 7.07 (t, *J* = 7.5 Hz, 1H), 6.71 (m, 2H), 5.42–5.24 (m, 1H), 4.97 (dd, *J* = 19.9, 13.6 Hz, 2H), 2.70 (d, *J* = 7.5 Hz, 2H), 1.54 (s, 9H), 1.22 (s, 9H); ^13^C-NMR (101 MHz, CDCl_3_) δ_C_ = 177.84, 156.13, 153.12, 139.88, 130.16, 128.36, 124.69, 123.00, 120.13, 109.35, 82.67, 81.39, 68.86 40.71, 28.26, 27.82; HRMS (ESI-MS) calculated for C_32_H_44_N_4_NaO_4_: 571.3260, found: 571.3310; chiral HPLC analysis: Chiralcel AD-H column, Hexane/iPrOH 90 : 10, flow rate = 1 mL/min, λ = 240 nm, retention times: 10.1, 16.1 min.

*Di-tert-butyl-1-[3-(2-ethoxy-2-oxoethyl)-2-oxoindolin-3-yl]-hydrazine-1,2-dicarboxylate* (**3ea**). Yellow oil; [α]_D_^28^ = −1.53 (c = 1.0, CHCl_3_, 85% *ee*); ^1^H-NMR (300 MHz, CDCl_3_) δ*_H_* = 8.06 (s, 1H), 7.74 (d, *J* = 7.4 Hz, 1H), 7.21 (t, *J* = 7.3 Hz, 1H), 7.05 (t, *J* = 7.6 Hz, 1H), 6.86–6.66 (m, 2H), 3.95 (q, *J* = 7.1 Hz, 2H), 3.08 (d, *J* = 14.2 Hz, 1H), 2.90 (d, *J* = 14.1 Hz, 1H), 1.54 (s, 9H), 1.24 (s, 9H), 1.04 (t, *J* = 7.1 Hz, 3H); ^13^C-NMR (101 MHz, CDCl_3_) δ_C_ = 168.30, 155.89, 152.80, 140.29, 129.70, 128.96, 125.33, 122.91, 109.67, 82.91, 81.56, 66.66, 60.95, 41.32, 28.27, 27.85, 13.83; HRMS (ESI-MS) calculated for C_34_H_48_N_4_NaO_8_: 663.3370, found: 663.3375; chiral HPLC analysis: Chiralcel AD-H column, Hexane/iPrOH 70 : 30, flow rate = 1 mL/min, λ = 240 nm, retention times: 8.4, 11.9 min.

*Di-tert-butyl (R)-1-(3-benzyl-2-oxoindolin-3-yl)-hydrazine-1,2-dicarboxylate* (**3fa**) [24]. White solid; [α]_D_^28^ = −7.83 (c = 1.0, CHCl_3_, 70% *ee*); ^1^H-NMR (300 MHz, CDCl_3_) δ*_H_ =* 7.95 (dd, *J* = 5.3, 3.2 Hz, 1H), 7.26 (s, 1H), 7.11 (dd, *J* = 5.6, 3.2 Hz, 2H), 7.06–6.88 (m, 4H), 6.71 (d, *J* = 7.0 Hz, 2H), 6.44 (dd, *J* = 5.5, 3.1 Hz, 1H), 3.32 (d, *J* = 12.0 Hz, 1H), 3.15 (d, *J* = 12.1 Hz, 1H), 1.58 (s, 9H), 1.23 (d, *J* = 2.6 Hz, 9H); ^13^C-NMR (101 MHz, CDCl_3_) δ_C_ = 177.66, 156.33, 152.80, 139.96, 132.90, 130.45, 128.41, 127.39, 126.75, 124.88, 122.81, 109.06, 82.69, 81.47, 69.96, 42.19, 28.29, 27.82; chiral HPLC analysis: Chiralcel AD-H column, Hexane/iPrOH 90 : 10, flow rate = 1 mL/min, λ = 240 nm, retention times: 8.9, 10.6 min.

*Di-tert-butyl* *(R)-1-(3-(4-methoxybenzyl)-2-oxoindolin-3-yl)-hydrazine-1,2-dicarboxylate* (**3ga**) [24]. Yellow oil; [α]_D_^28^ = −102.12 (c = 1.0, CHCl_3_, 93% *ee*); ^1^H-NMR (300 MHz, CDCl_3_) δ*_H_* = 7.97–7.88 (m, 1H), 7.11 (d, *J* = 4.1 Hz, 3H), 6.80 (s, 1H), 6.63 (d, *J* = 8.0 Hz, 2H), 6.53 (d, *J* = 8.1 Hz, 2H), 6.46 (s, 1H), 3.69 (s, 3H), 3.29 (d, *J* = 12.2 Hz, 1H), 3.10 (d, *J* = 12.1 Hz, 1H), 1.57 (s, 9H), 1.22 (s, 9H); ^13^C-NMR (101 MHz, CDCl_3_) δ_C_ = 177.67, 158.40, 156.29, 153.12, 131.42, 128.37, 124.81, 122.79, 112.84, 109.11, 82.66, 81.47, 69.93, 55.02, 41.36, 28.28, 27.83; chiral HPLC analysis: Chiralcel AD-H column, Hexane/iPrOH 90 : 10, flow rate = 1 mL/min, λ = 240 nm, retention times: 13.1, 15.9 min.

*Di-tert-butyl-1-[3-(4-nitrobenzyl)-2-oxoindolin-3-yl]-hydrazine-1,2-dicarboxylate* (**3ha**). Red oil; [α]_D_^28^ = −32.43 (c = 1.0, CHCl_3_, 80% *ee);* ^1^H-NMR (300 MHz, CDCl_3_) δ*_H_* = 7.97 (dd, *J* = 5.2, 3.3 Hz, 1H), 7.84 (d, *J* = 8.8 Hz, 2H), 7.14 (dd, *J* = 5.6, 3.2 Hz, 2H), 6.94 (s, 1H), 6.87 (d, *J* = 8.8 Hz, 2H), 6.46 (dd, *J* = 5.5, 3.1 Hz, 1H), 3.40 (d, *J* = 11.9 Hz, 1H), 3.22 (d, *J* = 11.9 Hz, 1H), 1.57 (s, 9H), 1.22 (s, 9H); ^13^C-NMR (101 MHz, CDCl_3_) δ_C_ = 177.18, 156.42, 152.93, 147.01, 140.96, 139.71, 131.34, 129.02, 124.92, 123.21, 122.52, 109.46, 83.06, 81.81, 69.57, 41.88, 28.28, 27.81; HRMS (ESI-MS) calculated for C_25_H_30_N_4_O_6_: 482.2165, found: 480.2332; chiral HPLC analysis: Chiralcel AD-H column, Hexane/iPrOH 90 : 10, flow rate = 1 mL/min, λ = 240 nm, retention times: 18.9, 28.6 min.

*Di-tert-butyl (R)-1-[02-oxo-3-(4-(trifluoromethyl)benzyl)-indolin-3-yl]hydrazine-1,2-dicarboxylate* (**3ia**) [24]. Yellow oil; [α]_D_^28^ = −98.62 (c = 1.0, CHCl_3_, 99% *ee);* ^1^H-NMR (300 MHz, CDCl_3_) δ*_H_* = 8.01–7.86 (bs, 1H), 7.40 (s, 1H), 7.22 (d, *J* = 8.1 Hz, 2H), 7.15–7.09 (m, 2H), 6.98 (s, 1H), 6.80 (d, *J* = 8.1 Hz, 2H), 6.43 (bs, 1H), 3.34 (d, *J* = 12.0 Hz, 1H), 3.18 (d, *J* = 12.0 Hz, 1H), 1.57 (s, 9H), 1.22 (s, 9H); ^13^C-NMR (101 MHz, CDCl_3_) δ_C_ = 177.32, 156.36, 152.98, 139.85, 137.24, 130.76, 130.24, 128.75, 124.84, 124.22, 122.98, 109.33, 82.88, 81.67, 69.67, 41.87, 28.27, 27.80; chiral HPLC analysis: Chiralcel AD-H column, Hexane/iPrOH 95 : 05, flow rate = 1 mL/min, λ = 240 nm, retention times: rt = 16.2, 20.4 min.

*Di-tert-butyl (R)-1-(5-methyl-2-oxo-3-phenyl-2,3-dihydrobenzofuran-3-yl)-hydrazine-1,2-dicarboxylate* (**11a**). Colorless oil; [α]_D_^28^ = −23.86 (c = 1.0, CHCl_3_, 55% *ee*); ^1^H-NMR (300 MHz, CDCl_3_) δ*_H_* = 7.88 (s, 1H), 7.57 (dd, *J* = 6.7, 2.9 Hz, 2H), 7.36–7.32 (m, 3H), 7.23–7.13 (m, 1H), 6.97 (d, *J* = 8.2 Hz, 1H), 6.28 (s, 1H), 2.47 (s, 3H), 1.34 (s, 9H), 1.29 (s, 9H); ^13^C-NMR (101 MHz, CDCl_3_) δ_C_ = 174.61, 154.62, 153.21, 150.91, 133.92, 132.85, 130.84, 130.13, 129.27, 129.02, 128.46, 128.31, 127.55, 110.73, 110.36, 83.85, 81.18, 71.45, 28.01, 27.78, 21.46; HRMS (ESI-MS) calculated for C_40_H_47_N_2_NaO_6_: 674.3332, found: 674.2301; chiral HPLC analysis: Chiralcel AD-H column, Hexane/iPrOH 70 : 30, flow rate = 1 mL/min, λ = 240 nm, retention times: 7.4, 16.9 min.

## 4. Conclusions

In summary, in this work we have described the use of readily available chiral *trans*-cyclohexanediamine-benzimidazole derivative (**5**) as an organocatalyst for the asymmetric electrophilic amination of 3-substituted oxindoles employing di-*t*-butylazodicarboxylate as the aminating agent. In general, for this challenging transformation, which avoids the need for protection of the oxindole substrate, the amination products were obtained in good yields and with enantioselectivities varying from good to high when 10 mol % of catalyst was employed. On the contrary, moderate enantioselectivities were achieved with benzofuranone derivatives. Finally, according to some experimental finds and assuming the bifunctional role of the catalyst, a possible catalytic cycle has been postulated. However, a more comprehensive study of the reaction mechanism is necessary.

## Figures and Tables

**Table 1:** Optimal condition screening ^a^.

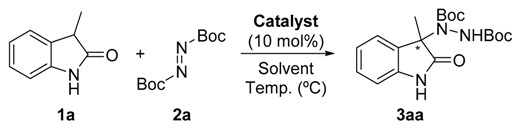					
Entry	Catalyst	Solvent	Temp. (°C)	Conv. (%) ^b^	*ee* (%) ^c^
1	4	PhMe	25	90	13
2	5	PhMe	25	80	73
3	6	PhMe	25	80	57
4	7	PhMe	25	92	5
5	8	PhMe	25	40	rac.
6	9	PhMe	25	87	3
7	5	CH_2_Cl_2_	25	80	56
8	5	Et_2_O	25	85	66
9	5	THF	25	75	50
10	5	C_6_H_14_	25	81	73
11	5	MeOH	25	79	5
12	5	PhMe	0 ^d^	89	81
13	5	C_6_H_14_	0 ^d^	82	51
14	5	PhMe	−20 ^d^	56	71
15	5	PhMe	−78 ^e^	59	70

^a^ Unless otherwise stated, general conditions were **1a** (0.15 mmol), **2a** (1.1 equiv.), organocatalyst (10 mol %) in toluene (1 mL) at the corresponding temperature for 24 h. ^b^ Conversions determined by ^1^H NMR from the crude reaction mixture. ^c^ Determined by chiral HPLC analysis (Daicel Chiralcel OD-H, see Materials and Methods section for details). ^d^ The reaction was run for 72 h. ^e^ The reaction was run for 96 h.

**Table 2:** Reaction scope ^a^.

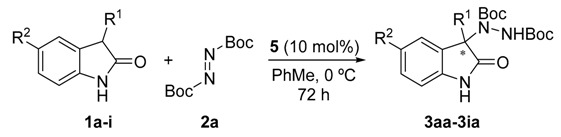				
Entry	1	3	Yield (%) ^b^	*ee* (%) ^c^
1	1a (R^1^ = Me, R^2^ = H)	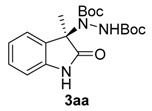	78	81
2	1b (R^1^ = Me, R^2^ = Br)	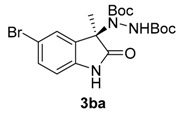	84	80
3	1c (R^1^ = Ph, R^2^ = H)	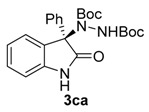	61	70
4	1d (R^1^ = Allyl, R^2^ = H)	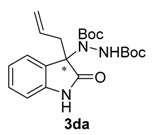	87	84
5	1e (R^1^ = CH_2_CO_2_Et, R^2^ = H)	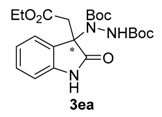	71	85
6	1f (R^1^ = Bn, R^2^ = H)	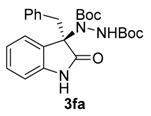	83	70
7	1g (R^1^ = *p*-MeOBn, R^2^ = H)	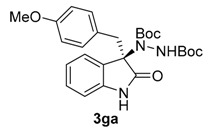	75	93
8	1h (R^1^ = *p*-NO_2_Bn, R^2^ = H)	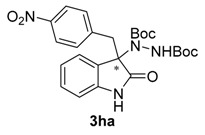	79	80
9	1i (R^1^ = *p*-CF_3_Bn, R^2^ = H)	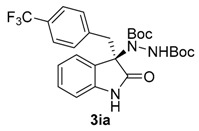	76	99

^a^ Unless otherwise stated, general conditions were **1** (0.15 mmol), **2a** (1.1 equiv.), **5** (10 mol %) in the corresponding toluene (1 mL) at 0 ºC for 72 h. ^b^ Isolated yield after column chromatography. ^c^ Determined by chiral HPLC analysis (see Materials and Methods section for details).

## Supplementary Materials

The following are available online. General remarks and as well as NMR spectra and HPLC charts of amination compounds are provided.
