# Supplementary material for: Chiral 2-Aminobenzimidazole as Bifunctional Catalyst in the Asymmetric Electrophilic Amination of Unprotected 3-Substituted Oxindoles
Source: Molecules. 2018 Jun 6;23(6):1374. doi: 10.3390/molecules23061374 (PMC6099788; doi:10.3390/molecules23061374)
Supplement: Supplementary file 1 [file molecules-23-01374-s001.pdf]

SUPPORTING INFORMATION

Chiral 2-Aminobenzimidazole as Bifunctional Catalyst in the Asymmetric Electrophilic Amination of Unprotected 3-Substituted Oxindoles

Llorenç Benavent [1], Alejandro Baeza [1,*], Megan Freckleton [1]

Departamento de Química Orgánica, Facultad de Ciencias and Instituto de Síntesis Orgánica,

Alicante University. Aptdo. 99. E-03080, Alicante, Spain.

General Remarks and General Procedure 1

Physical and Spectroscopical Data of 3-Substituted Oxindoles 2

^{1}H-NMR Spectra of 3-Substituted Oxindoles 5

NMR Spectra of Amination Products 15

HPLC Chromatograms of Representative Compounds 28

General Remarks:

All reagents were purchased from commercial sources and used without further purification. Substrates which were not commercially available were synthesized according to known literature procedures. Since the 3-substituted oxindoles were described in the literature, only ^{1}H NMR is listed (see NMR spectra below). NMR spectra were performed on a Bruker AC-300 or Bruker Avance-400 400 (Bruker Corporation) using $CDCl_3$ as solvent and TMS as internal standard unless otherwise stated. Optical rotations were measured on a Jasco P-1030 Polarimeter with a 5 cm cell (c given in g/100 mL). Enantioselectivities were determined by HPLC analysis (Agilent 1100 Series HPLC) equipped with a G1315B diode array detector and a Quat Pump G1311A (Agilent Technologies) equipped with the corresponding Daicel chiral column; the retention time of the major enantiomer is highlighted in bold. Analytical TLC was performed on Merck silica gel plates and the spots visualized with UV light at 254 nm (Merck millipore). Flash chromatography employed Merck silica gel 60 (0.040-0.063 mm) (Merck millipore).

General Procedure for the Asymmetric Amination of 3-Substituted Oxindoles

In an open-air tube at room temperature (25 ºC), the corresponding 3-substituted-oxindole (0.15 mmol) was added to a solution of organocatalyst 5 (10 mol%) in toluene (1 mL). After 5 minutes, the mixture was introduced into a cooling bath at 0 ºC, and after two minutes di-*tert*-butylazodicarboxilate (0.11 mmol, 1.1 eq.) was added in one portion. The reaction was then allowed to react for 3 days. After this time, water (5 mL) and ethyl acetate (5 mL) were added, and the aqueous layer was then re-extracted twice (2 x 10 mL). The combined organic phases were dried ($MgSO_4$) and evaporated under reduced pressure. Finally, the reaction crude was purified by column chromatography on silica gel or TLC preparative using hexane/ethyl acetate mixtures as eluent. Spectroscopic data are listed within the article.

Physical and Spectroscopical Data of 3-substituted oxindoles

***tert*-Butyl 3-methyl-2-oxoindoline-1-carboxylate (1a`).**[1]

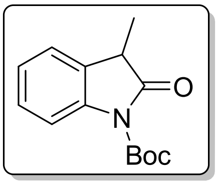

Yellow oil. (90% Yield)

^{1}H-NMR (300 MHz, $CDCl_3$): δ_H = 7.43 (d, J = 7.7 Hz, 1H), 7.33 (dd, J = 7.6, 1.6 Hz, 1H), 7.30–7.18 (m, 2H), 3.92 (q, J = 7.0 Hz, 1H), 1.50 (d, J = 9.1 Hz, 12H); ^{13}C-NMR (101 MHz, $CDCl_3$) δ_C = 177.33, 149.04, 138.68, 129.78, 124.99, 123.45, 115.28, 73.40, 29.68, 28.04, 25.68.

5-bromo-3-methylindolin-2-one (1b):[2]

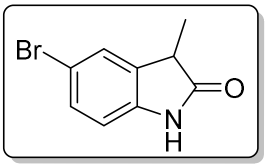

White solid. (>99% Yield)

^{1}H-NMR (300 MHz, $CDCl_3$) δ_H = 8.88 (s, 1H), 7.40–7.32 (m, 2H), 6.81 (d, J = 8.8 Hz, 1H), 3.49 (q, J = 7.6 Hz, 1H), 1.51 (d, J = 7.7 Hz, 3H); ^{13}C-NMR (101 MHz, $CDCl_3$) δ_C = 180.99, 140.23, 133.28, 130.78, 127.09, 115.06, 111.27, 41.19, 15.10.

3-Phenylindolin-2-one (1c):[2]

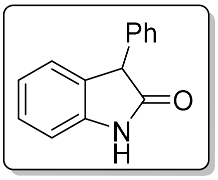

Pale yellow solid. (78% Yield)

^{1}H-NMR (300 MHz, $CDCl_3$): δ_H = 8.25 (bs, 1H), 7.39–7.25 (m, 4H), 7.24–7.21 (m, 2H), 7.14 (d, J = 7.4 Hz, 1H), 7.04 (td, J = 7.5, 0.9 Hz, 1H), 6.94 (d, J = 7.8 Hz, 1H), 4.64 (s, 1H); ^{13}C-NMR (101 MHz, $CDCl_3$) δ_C = 179.27, 141.84, 136.51, 129.69, 128.99, 128.54, 128.41, 127.69, 125.18, 122.70, 110.24, 52.86.

3-allylindolin-2-one (1d):[3]

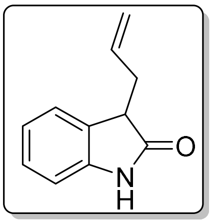

Pale yellow solid. (46% Yield)

^{1}H-NMR (300 MHz, $CDCl_3$) δ_H = 8.95 (s, 1H), 7.27 (d, J = 7.4 Hz, 1H), 7.22 (t, J = 7.7 Hz, 1H), 7.02 (td, J = 7.6, 0.8 Hz, 1H), 6.91 (d, J = 7.7 Hz, 1H), 5.86–5.69 (m, 1H), 5.09 (ddd, J = 13.6, 10.7, 0.9 Hz, 2H), 3.54 (dd, J = 7.3, 5.0 Hz, 1H), 2.91–2.78 (m, 1H), 2.60 (dt, J = 14.4, 7.7 Hz, 1H); ^{13}C-NMR (101 MHz, $CDCl_3$) δ_C = 180.34, 141.67, 133.87, 129.25, 127.97, 124.38, 122.21, 118.10, 109.91, 45.83, 34.74.

Ethyl 2-(2-oxoindolin-3-yl)-acetate (1e):[4]

[1] Santanu Ghosh, S.; Chaudhuri, S.; Bisai, A. *Org. Lett.* **2015**, *17*, 1373-1376.
[2] Cheng, L.; Liu, L.; Wang, D.; Chen, Y-J. *Org. Lett.* **2009**, *Vol. 11,No. 17*, 3874-3877.
[3] Prakash D., C.; Bor-Cherng, H. and Gene-Hsiang L. *Org. Lett.* **2017**, *19*, 6112-6115.
[4] Cao, S-H.; Zhang, X-C.; Wei, Y. and Shi, M. *Eur. J. Org. Chem.* **2011**, 2668–2672.

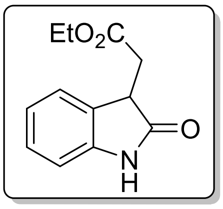

Red solid. (82% Yield)

^{1}H-NMR (300 MHz, $CDCl_3$) δ_H = 8.57 (bs, 1H), 7.22 (t, *J* = 7.6 Hz, 2H), 7.01 (t, *J* = 8.0 Hz, 1H), 6.90 (d, *J* = 7.6 Hz, 1H), 4.21–4.09 (m, 2H), 3.82 (dd, *J* = 7.8, 4.5 Hz, 1H), 3.08 (dd, *J* = 16.9, 4.5 Hz, 1H), 2.84 (dd, *J* = 16.9, 7.8 Hz, 1H), 1.21 (t, *J* = 7.1 Hz, 3H); ^{13}C-NMR (101 MHz, $CDCl_3$) δ_C = 179.56, 171.07, 141.66, 128.30, 124.19, 124.03, 122.45, 110.02, 61.02, 42.41, 34.76, 14.06.

3-benzylindolin-2-one (1f):[2]

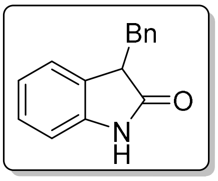

White solid. (82.8% Yield)

^{1}H-NMR (300 MHz, $CDCl_3$): δ_H = 8.40 (s, 1H), 7.26–7.13 (m, 6H), 6.90 (td, *J* = 7.6, 1.0 Hz, 1H), 6.83 (d, *J* = 7.8 Hz, 1H), 6.74 (d, *J* = 7.4 Hz, 1H), 3.74 (dd, *J* = 9.2, 4.5 Hz, 1H), 3.49 (dd, *J* = 13.7, 4.6 Hz, 1H), 2.93 (dd, *J* = 13.7, 9.2 Hz, 1H); ^{13}C-NMR (101 MHz, $CDCl_3$) δ_C = 179.77, 141.41, 137.78, 129.42, 128.97, 128.34, 127.97, 126.68, 124.83, 122.05, 109.79, 47.56, 36.62.

3-(4-methoxybenzyl)-indolin-2-one (1g):[2]

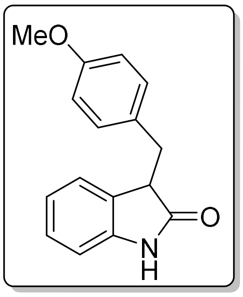

Yellow solid. (213 mg, 84% Yield)

^{1}H-NMR (300 MHz, $CDCl_3$) δ_H = 8.77 (bs, 1H), 7.20–7.12 (m, 1H), 7.07 (d, *J* = 8.7 Hz, 2H), 6.91 (td, *J* = 7.6, 1.0 Hz, 1H), 6.84 (d, *J* = 7.7 Hz, 1H), 6.81–6.74 (m, 3H), 3.76 (s, 3H), 3.70 (dd, *J* = 9.1, 4.5 Hz, 1H), 3.41 (dd, *J* = 13.8, 4.6 Hz, 1H), 2.90 (dd, *J* = 13.8, 9.0 Hz, 1H); ^{13}C-NMR (101 MHz, $CDCl_3$) δ_C = 179.78, 158.31, 141.46, 130.40, 129.70, 129.07, 127.91, 124.82, 122.01, 113.69, 109.77, 55.18, 47.77, 35.74.

3-(4-nitrobenzyl)-indolin-2-one (1h):[2]

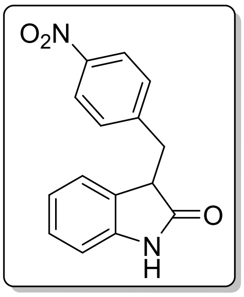

Brown solid. (167 mg, 62% Yield)

^{1}H-NMR (300 MHz, $CDCl_3$) δ_H = 8.07 (d, *J* = 8.8 Hz, 2H), 7.91 (s, 1H), 7.30 (d, *J* = 8.7 Hz, 2H), 7.19 (dd, *J* = 11.6, 5.2 Hz, 1H), 7.01–6.90 (m, 2H), 6.80 (d, *J* = 7.8 Hz, 1H), 3.82 (dd, *J* = 7.6, 4.9 Hz, 1H), 3.49 (dd, *J* = 13.7, 4.9 Hz, 1H), 3.24 (dd, *J* = 13.7, 7.7 Hz, 1H); ^{13}C-NMR (101 MHz, $CDCl_3$) δ_C = 178.17, 145.11, 141.12, 130.35, 128.50, 124.50, 123.46, 122.45, 109.89, 46.84, 36.06.

3-[4-(trifluoromethyl)benzyl]indolin-2-one (1i)[2]

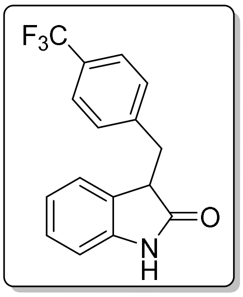

Yellow solid. (67% Yield)

^{1}H-NMR (300 MHz, $CDCl_3$) δ_H = 8.51 (bs, 1H), 7.49 (d, *J* = 8.0 Hz, 2H), 7.27 (d, *J* = 7.2 Hz, 2H), 7.19 (t, *J* = 7.7 Hz, 1H), 6.95 (td, *J* = 7.6, 0.8 Hz, 1H), 6.84 (dd, *J* = 7.6, 2.9 Hz, 2H), 3.78 (dd, *J* = 8.4, 4.7 Hz, 1H), 3.48 (dd, *J* = 13.8, 4.6 Hz, 1H), 3.10 (dd, *J* = 13.8, 8.4 Hz, 1H); ^{13}C-NMR (101 MHz, $CDCl_3$) δ_C = 178.70, 141.67, 141.21, 129.78, 128.28, 125.24, 125.21, 124.62, 122.30, 109.82, 47.06, 36.20.

5-methyl-3-phenylbenzofuran-2(3H)-one (10).[5]

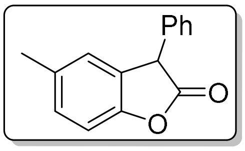

White solid; (100 mg, 44% Yield)

^{1}H-NMR (300 MHz, $CDCl_3$) δ_H = 7.39–7.31 (m, 3H), 7.22 (dt, *J* = 5.3, 2.1 Hz, 2H), 7.17–7.12 (m, 1H), 7.06 (d, *J* = 8.2 Hz, 1H), 7.00 (d, *J* = 0.6 Hz, 1H), 4.84 (s, 1H), 2.32 (s, 3H); ^{13}C-NMR (101 MHz, $CDCl_3$) δ_C = 175.44, 151.86, 135.35, 134.20, 129.76, 129.13, 128.31, 128.17, 127.00, 125.74, 110.46, 49.94, 21.11.

[5] Bhaskar B., D.; Manoj K., C. and Sandip K., N. *Synth. Commun.* **2016**, *46*, 1772-1780.

^{1}H-NMR SPECTRA OF 3-SUBSTITUTED OXINDOLES

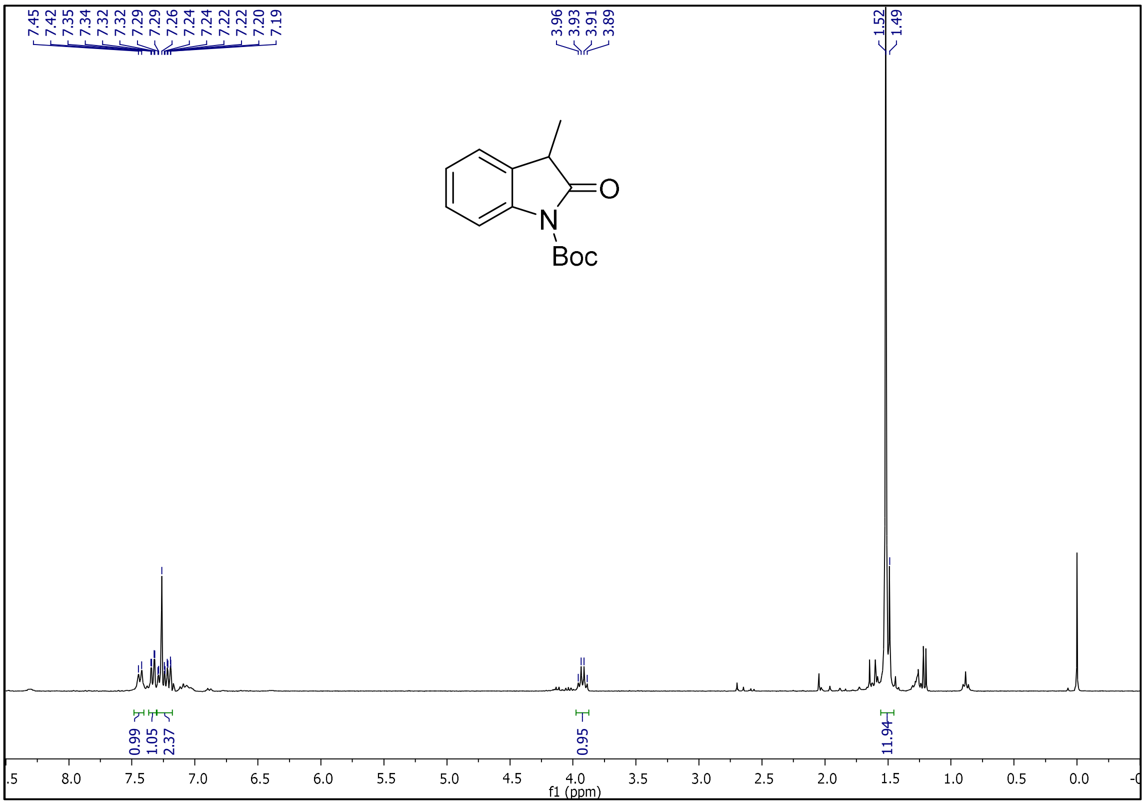

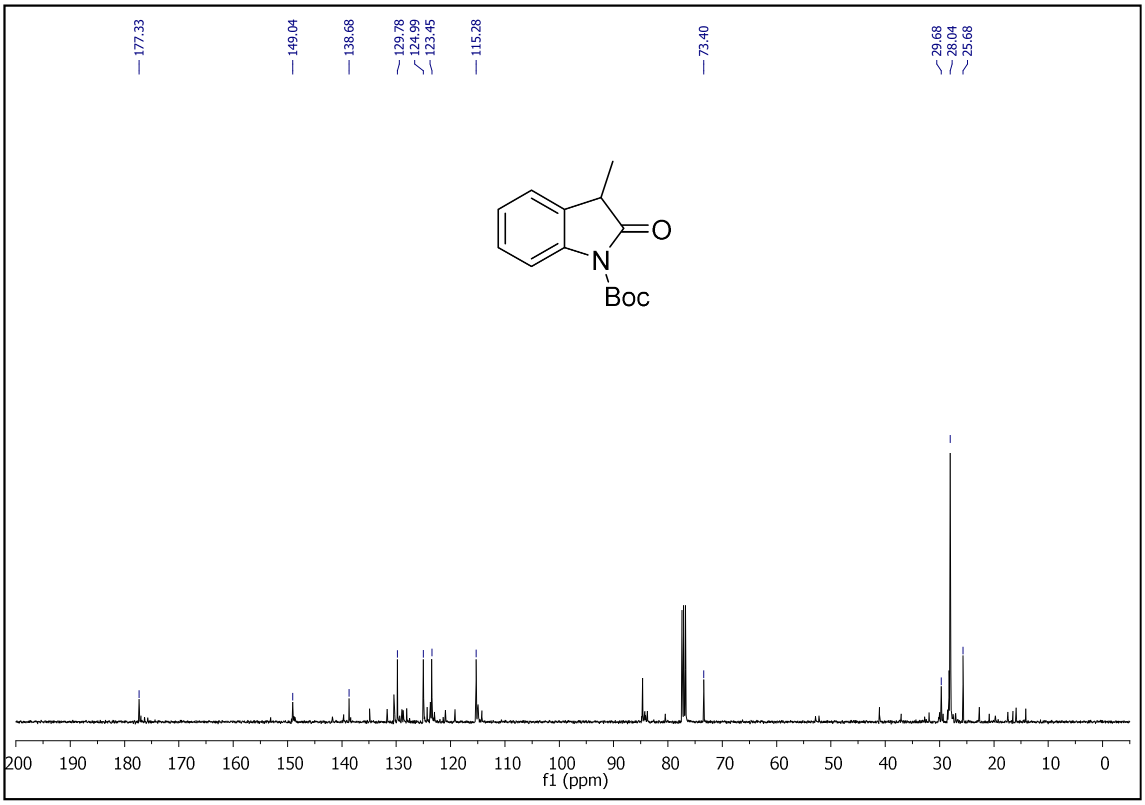

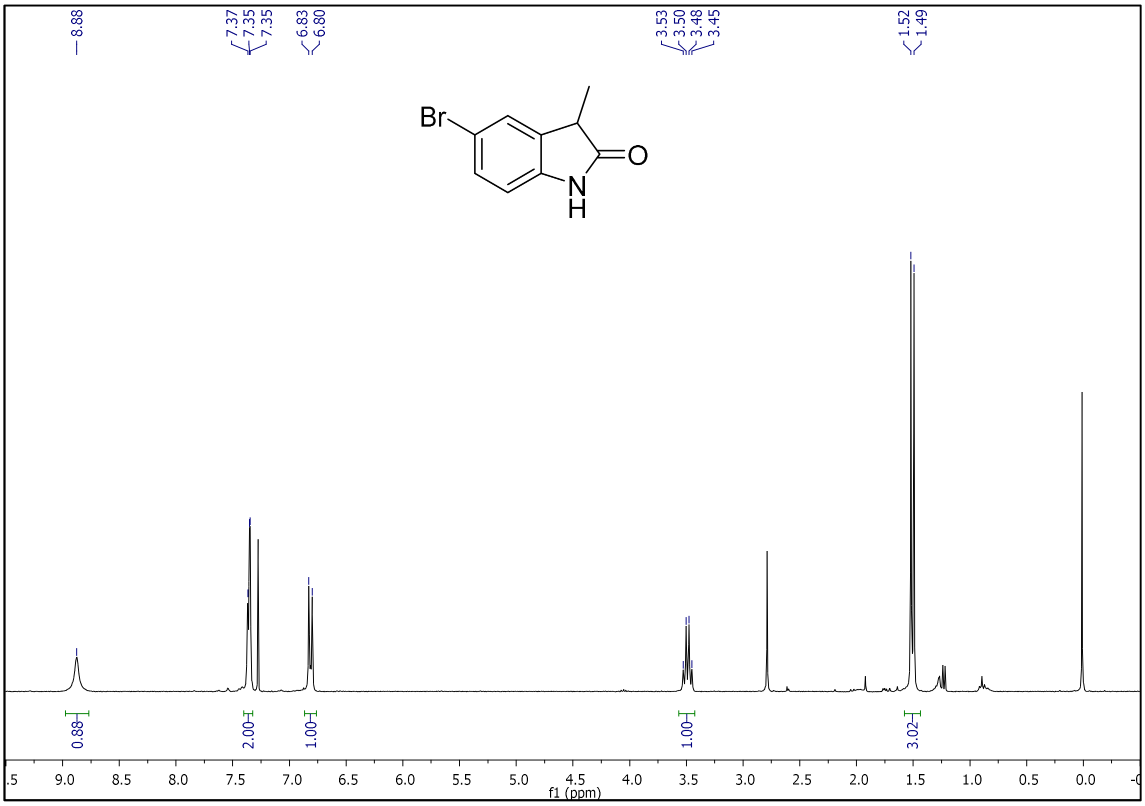
8.88
7.37
7.35
7.35
6.83
6.80
3.53
3.50
3.48
3.45
1.52
1.49
Br
O
N
H
0.88
2.00
1.00
1.00
3.02
f1 (ppm)

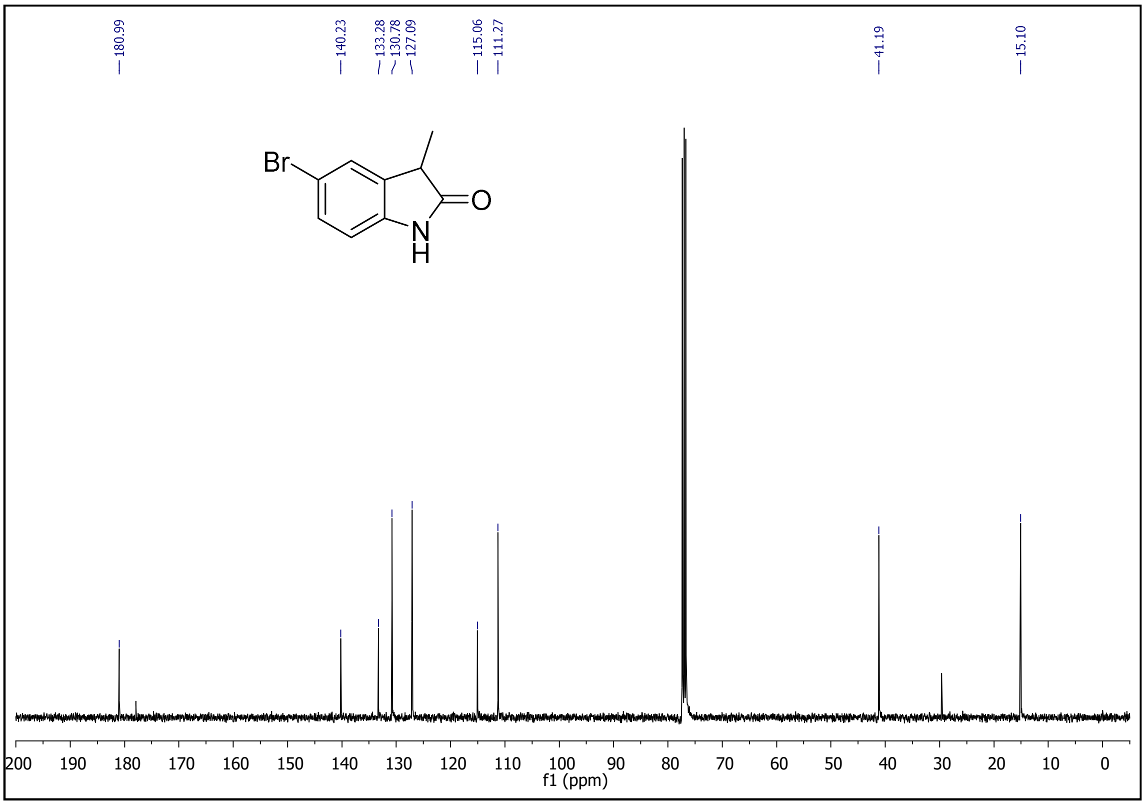
180.99
140.23
133.28
130.78
127.09
115.06
111.27
41.19
15.10
Br
O
N
H
f1 (ppm)

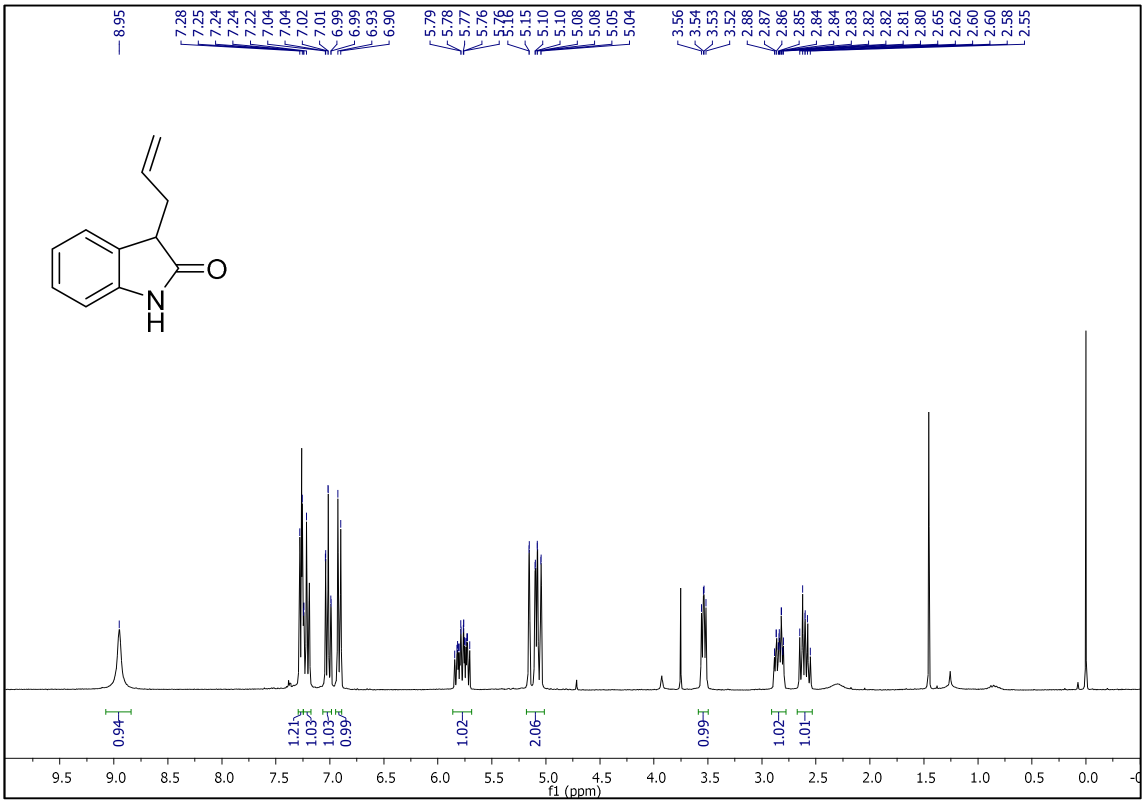
8.95
7.28 7.25 7.24 7.24 7.22 7.04 7.02 7.01 6.99 6.99 6.93 6.90
5.79 5.78 5.77 5.76 5.76 5.16 5.15 5.10 5.08 5.08 5.05 5.04
3.56 3.54 3.53 3.52 2.88 2.87 2.86 2.85 2.84 2.84 2.83 2.82 2.82 2.81 2.80 2.65 2.62 2.60 2.60 2.58 2.55
O
N
H
0.94
1.21
1.03
1.03
0.99
1.02
2.06
0.99
1.02
1.01
f1 (ppm)

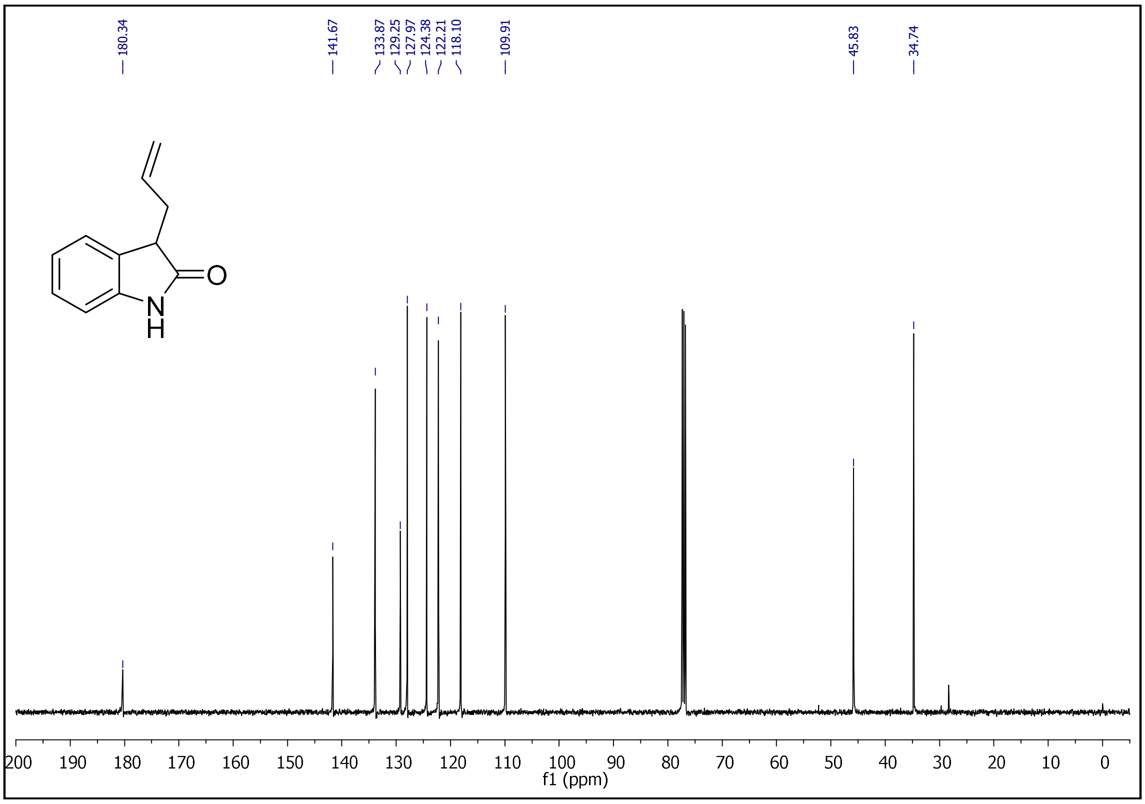
180.34
141.67
133.87 129.25 127.97 124.38 122.21 118.10
109.91
45.83
34.74
O
N
H
f1 (ppm)

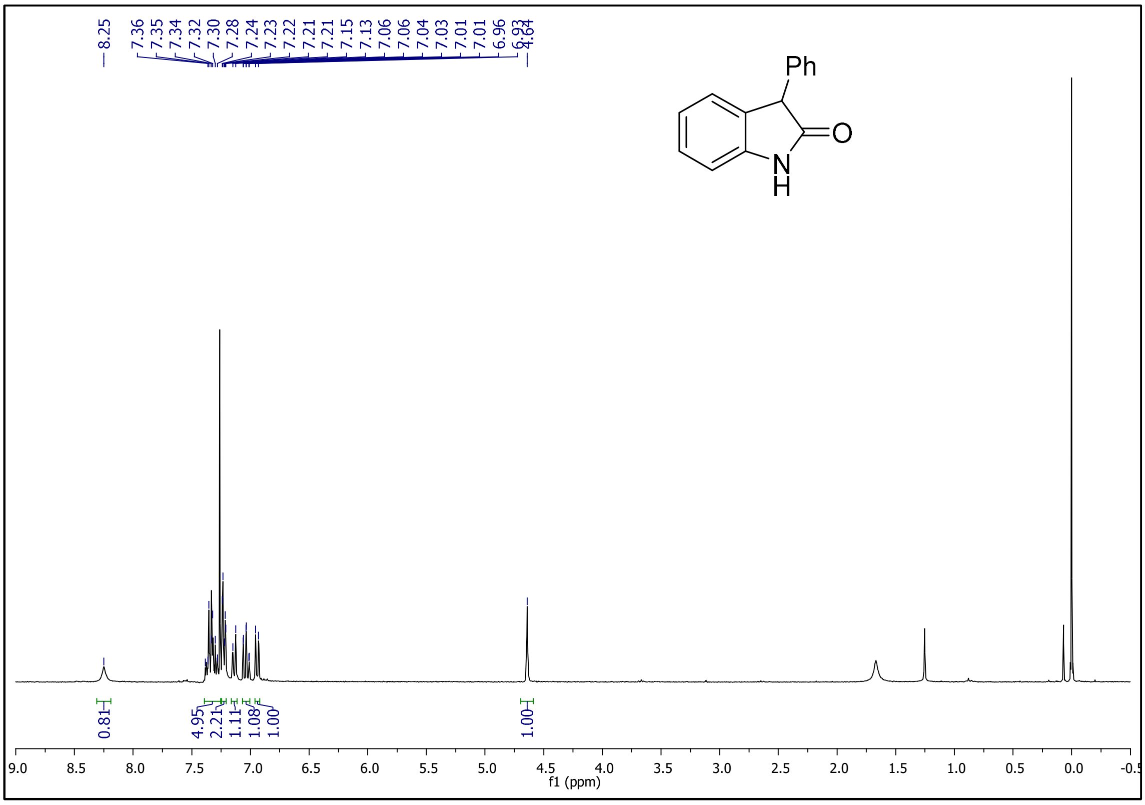
8.25
7.36
7.35
7.34
7.32
7.30
7.28
7.24
7.23
7.22
7.21
7.21
7.15
7.13
7.06
7.06
7.04
7.03
7.01
7.01
6.96
6.93
4.64
Ph
O
N
H
0.81
4.95
2.21
1.11
1.08
1.00
1.00
9.0 8.5 8.0 7.5 7.0 6.5 6.0 5.5 5.0 4.5 4.0 3.5 3.0 2.5 2.0 1.5 1.0 0.5 0.0
f1 (ppm)

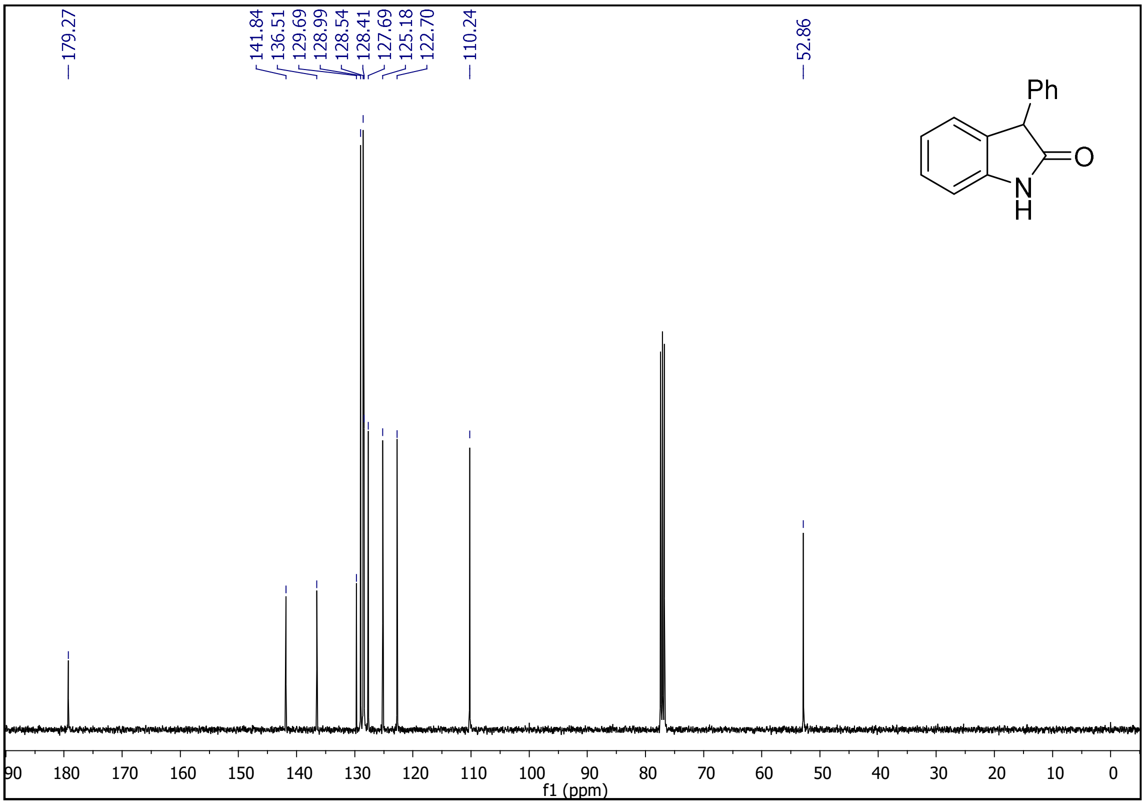
179.27
141.84
136.51
129.69
128.99
128.54
128.41
127.69
125.18
122.70
110.24
52.86
Ph
O
N
H
90 180 170 160 150 140 130 120 110 100 90 80 70 60 50 40 30 20 10 0
f1 (ppm)

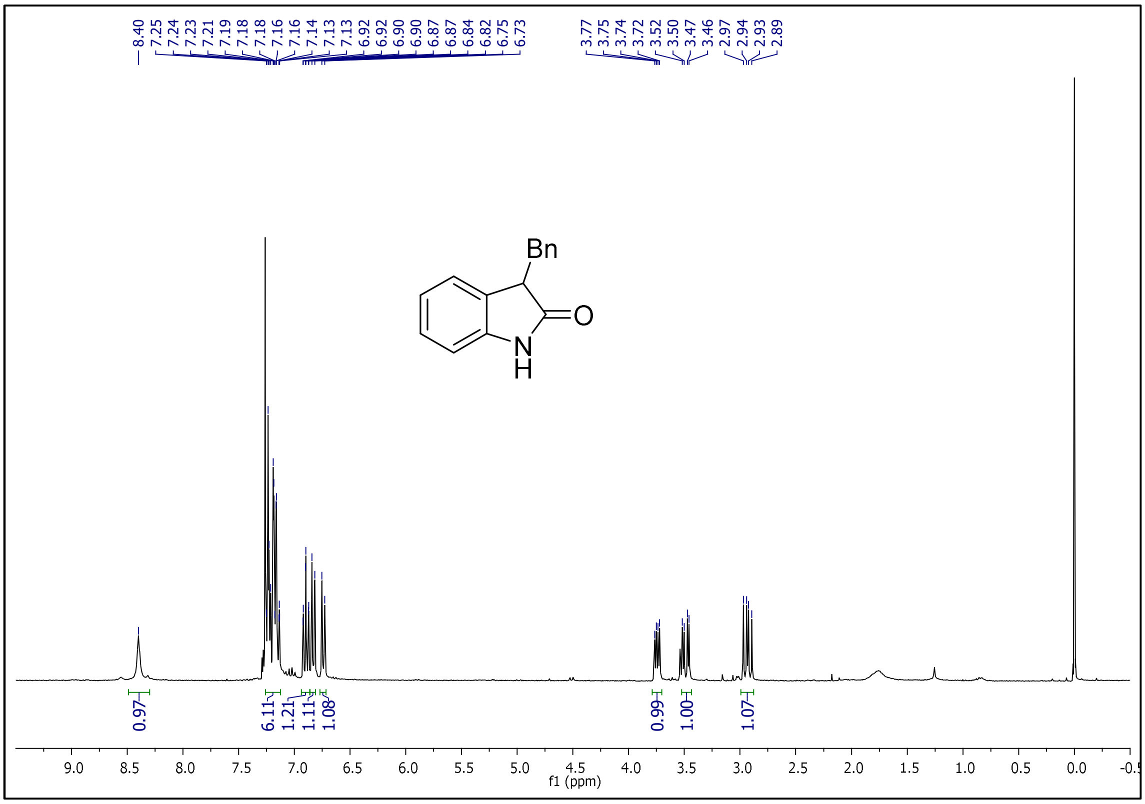
8.40
7.25
7.24
7.23
7.21
7.19
7.18
7.16
7.16
7.14
7.13
7.13
6.92
6.92
6.90
6.90
6.87
6.87
6.84
6.82
6.75
6.73
3.77
3.75
3.74
3.72
3.52
3.50
3.47
3.46
2.97
2.94
2.93
2.89
Bn
O
N
H
0.97
6.11
1.21
1.11
1.08
0.99
1.00
1.07
9.0
8.5
8.0
7.5
7.0
6.5
6.0
5.5
5.0
4.5
4.0
3.5
3.0
2.5
2.0
1.5
1.0
0.5
0.0
f1 (ppm)

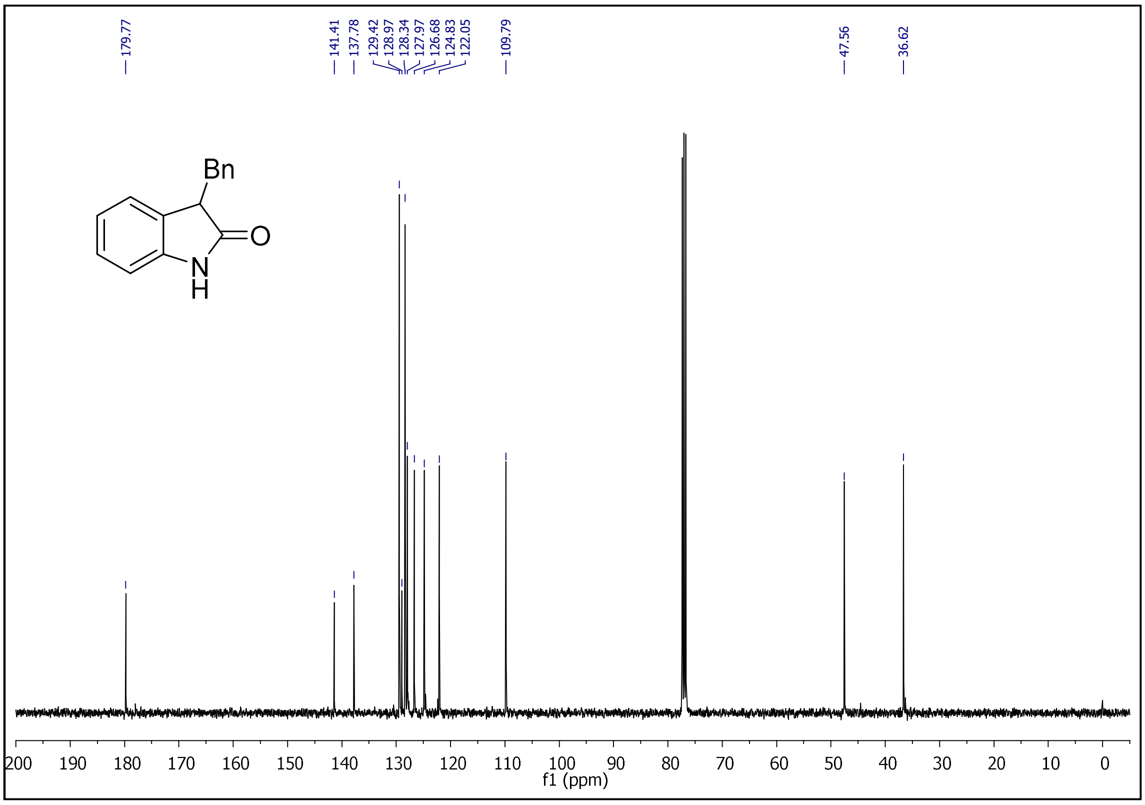
179.77
141.41
137.78
129.42
128.97
128.34
127.97
126.68
124.83
122.05
109.79
47.56
36.62
Bn
O
N
H
200
190
180
170
160
150
140
130
120
110
100
90
80
70
60
50
40
30
20
10
0
f1 (ppm)

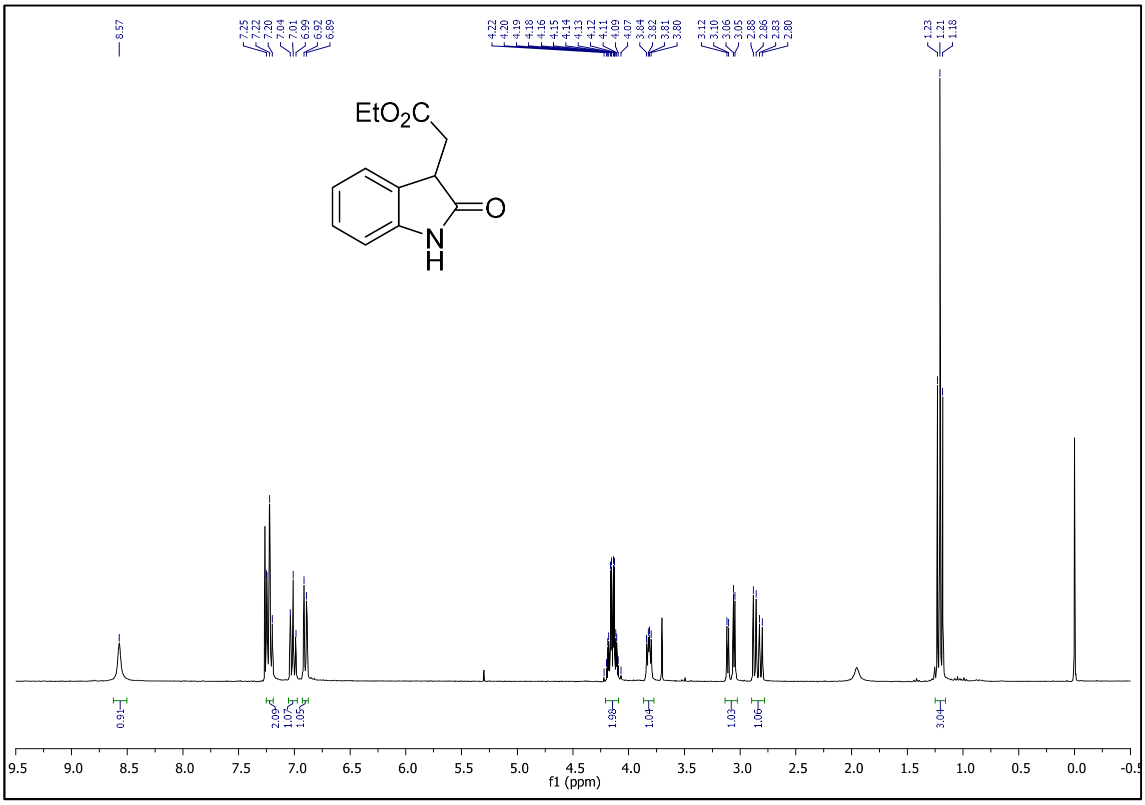
8.57
7.25
7.22
7.20
7.04
7.01
6.99
6.92
6.89
4.22
4.20
4.19
4.18
4.16
4.15
4.14
4.13
4.12
4.11
4.09
4.07
3.84
3.82
3.81
3.80
3.12
3.10
3.06
3.05
2.88
2.86
2.83
2.80
1.23
1.21
1.18
EtO2C
N
H
O
0.91
2.09
1.07
1.05
1.98
1.04
1.03
1.06
3.04
f1 (ppm)

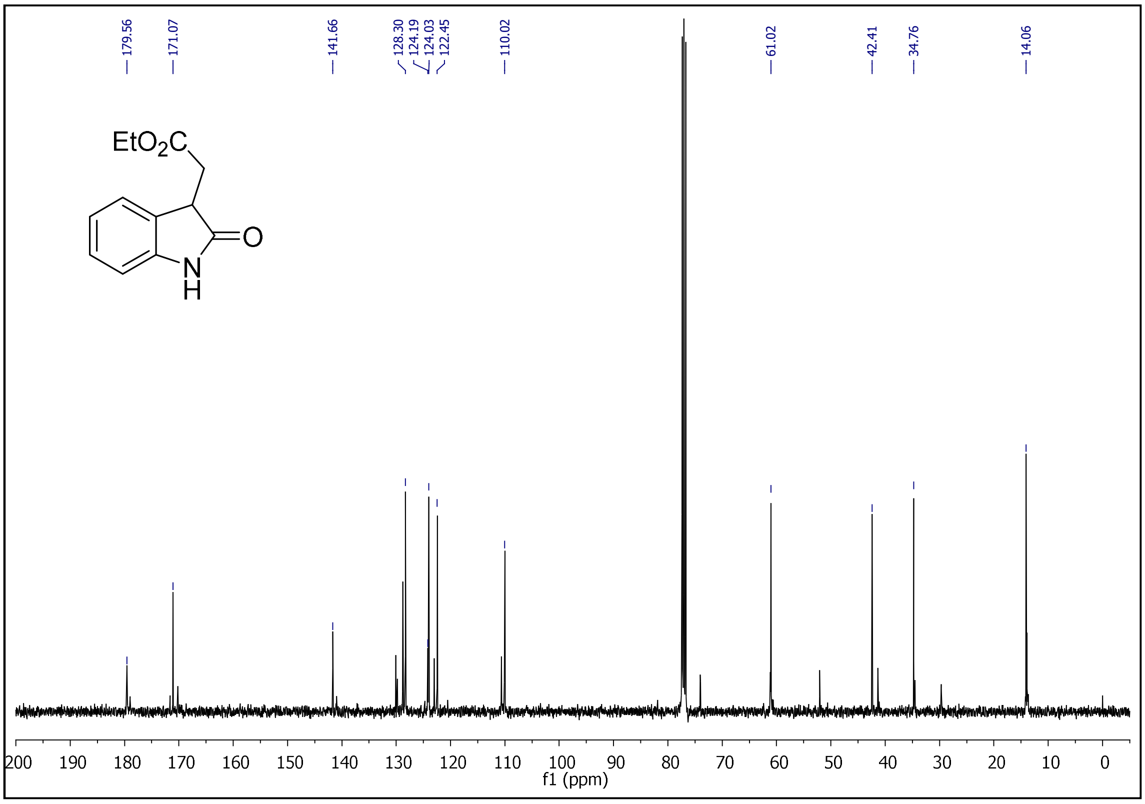
179.56
171.07
141.66
128.30
124.19
124.03
122.45
110.02
61.02
42.41
34.76
14.06
EtO2C
N
H
O
f1 (ppm)

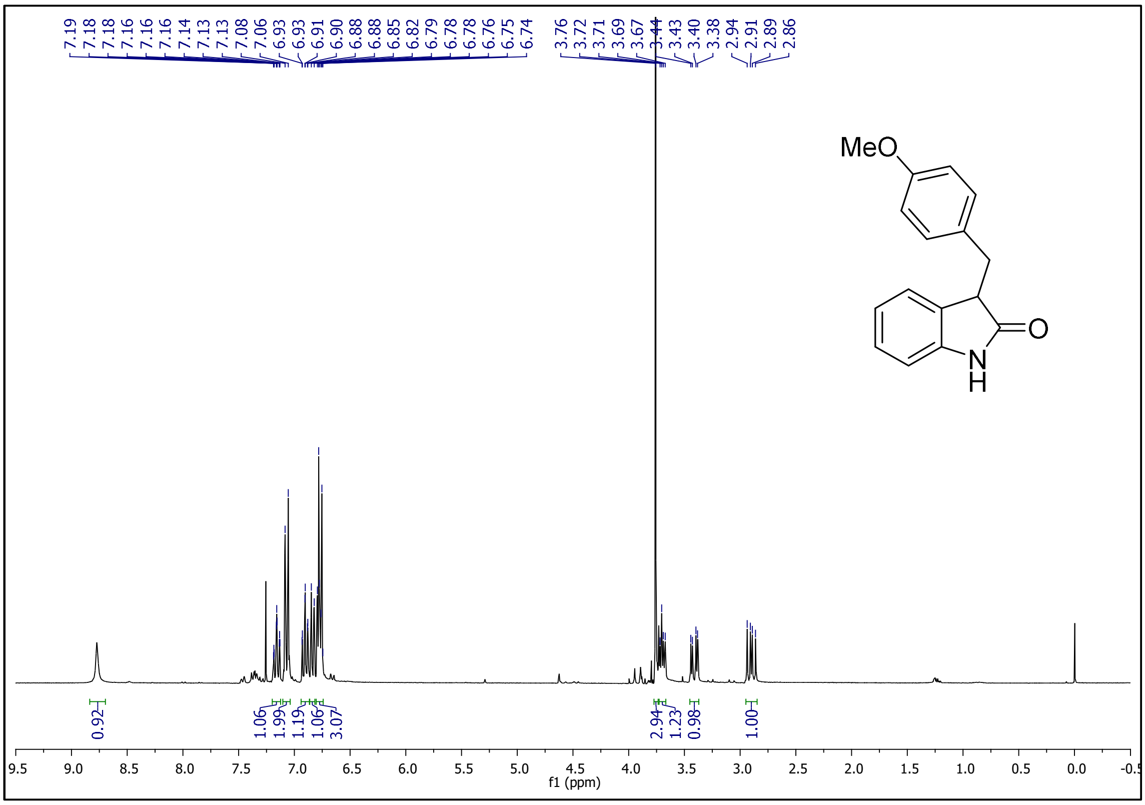
7.19 7.18 7.18 7.16 7.16 7.14 7.13 7.13 7.08 7.06 6.93 6.93 6.91 6.90 6.88 6.88 6.85 6.82 6.79 6.78 6.78 6.76 6.75 6.74
3.76 3.72 3.71 3.69 3.67 3.44 3.43 3.40 3.38 2.94 2.91 2.89 2.86
MeO
O
N
H
0.92
1.06 1.99 1.19 1.06 3.07
2.94 1.23 0.98
1.00
9.5 9.0 8.5 8.0 7.5 7.0 6.5 6.0 5.5 5.0 4.5 4.0 3.5 3.0 2.5 2.0 1.5 1.0 0.5 0.0
f1 (ppm)

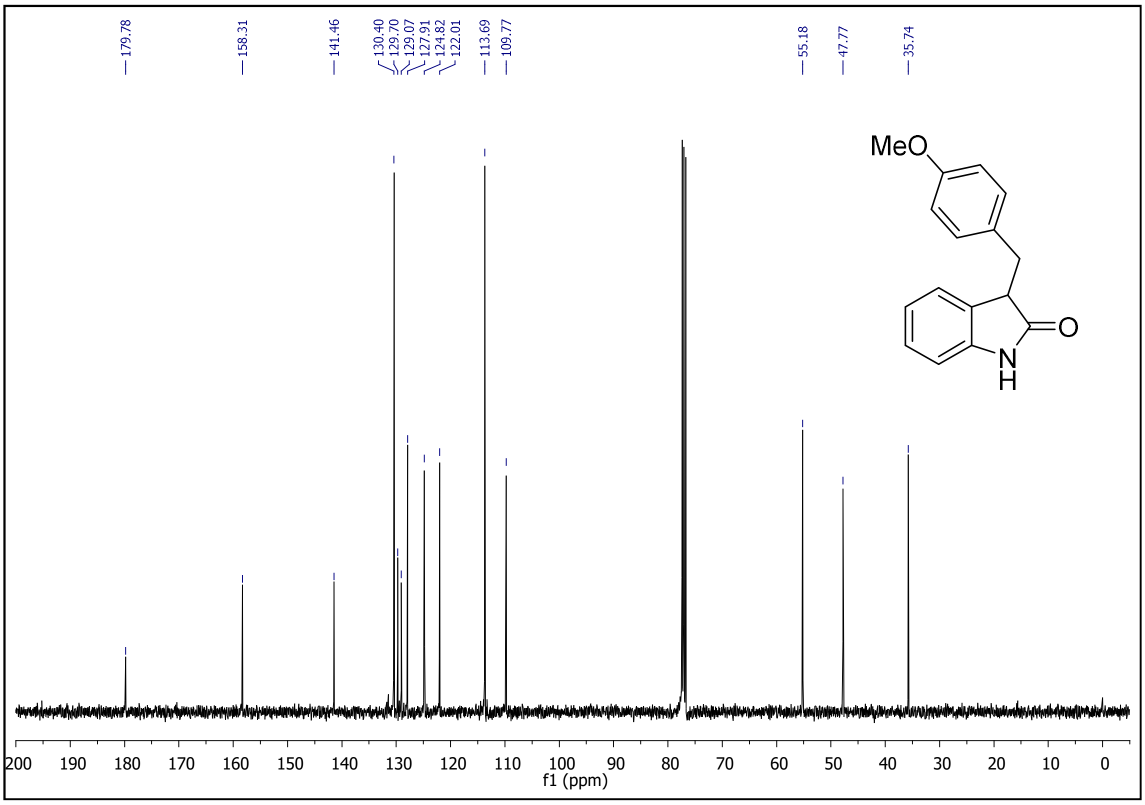
179.78
158.31
141.46
130.40 129.70 129.07 127.91 124.82 122.01
113.69 109.77
55.18
47.77
35.74
MeO
O
N
H
200 190 180 170 160 150 140 130 120 110 100 90 80 70 60 50 40 30 20 10 0
f1 (ppm)

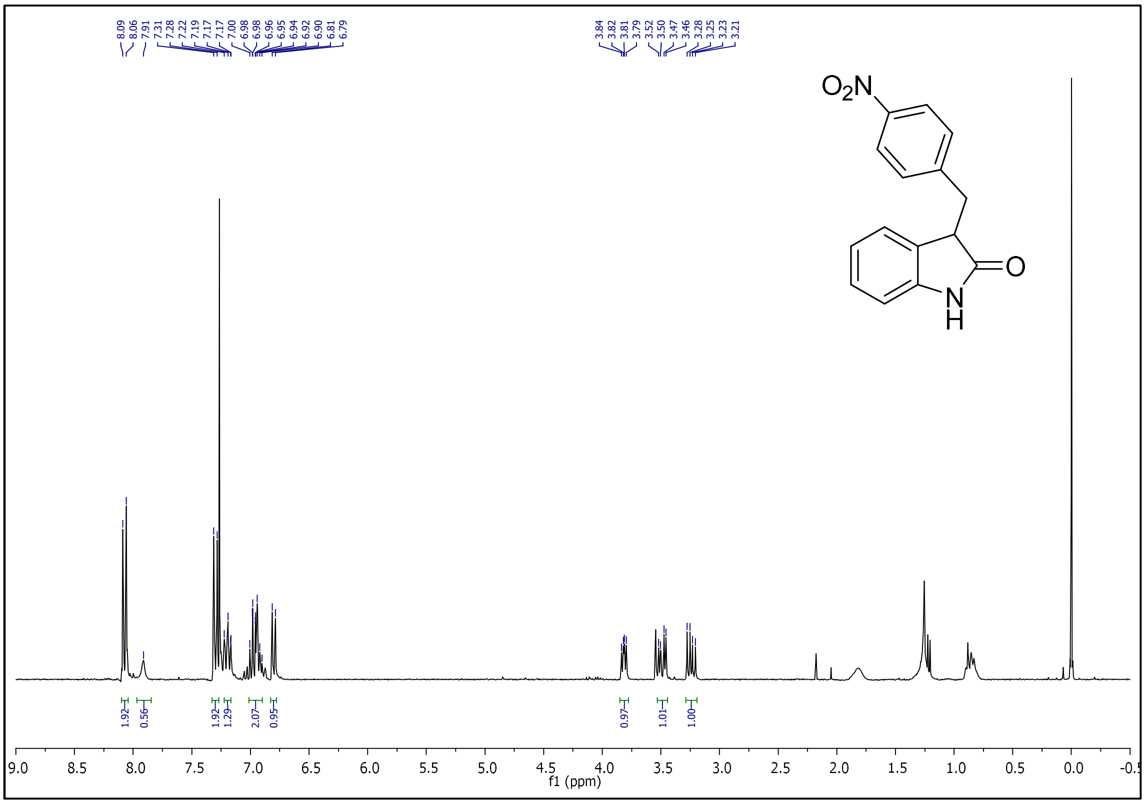
8.09
8.06
7.91
7.31
7.28
7.22
7.19
7.17
7.17
7.00
6.98
6.98
6.96
6.95
6.94
6.92
6.90
6.81
6.79
3.84
3.82
3.81
3.79
3.52
3.50
3.47
3.46
3.28
3.25
3.23
3.21
O2N
N
H
O
1.92
0.56
1.92
1.29
2.07
0.95
0.97
1.01
1.00
f1 (ppm)

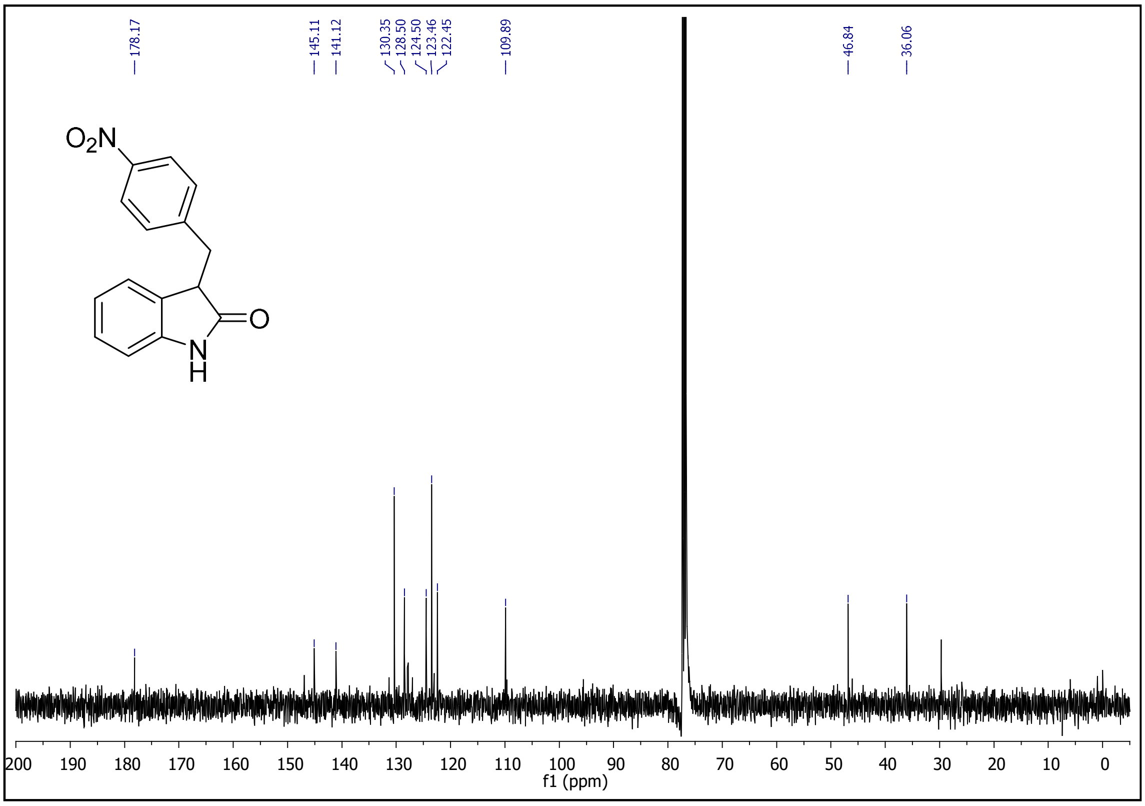
178.17
145.11
141.12
130.35
128.50
124.50
123.46
122.45
109.89
46.84
36.06
O2N
N
H
O
f1 (ppm)

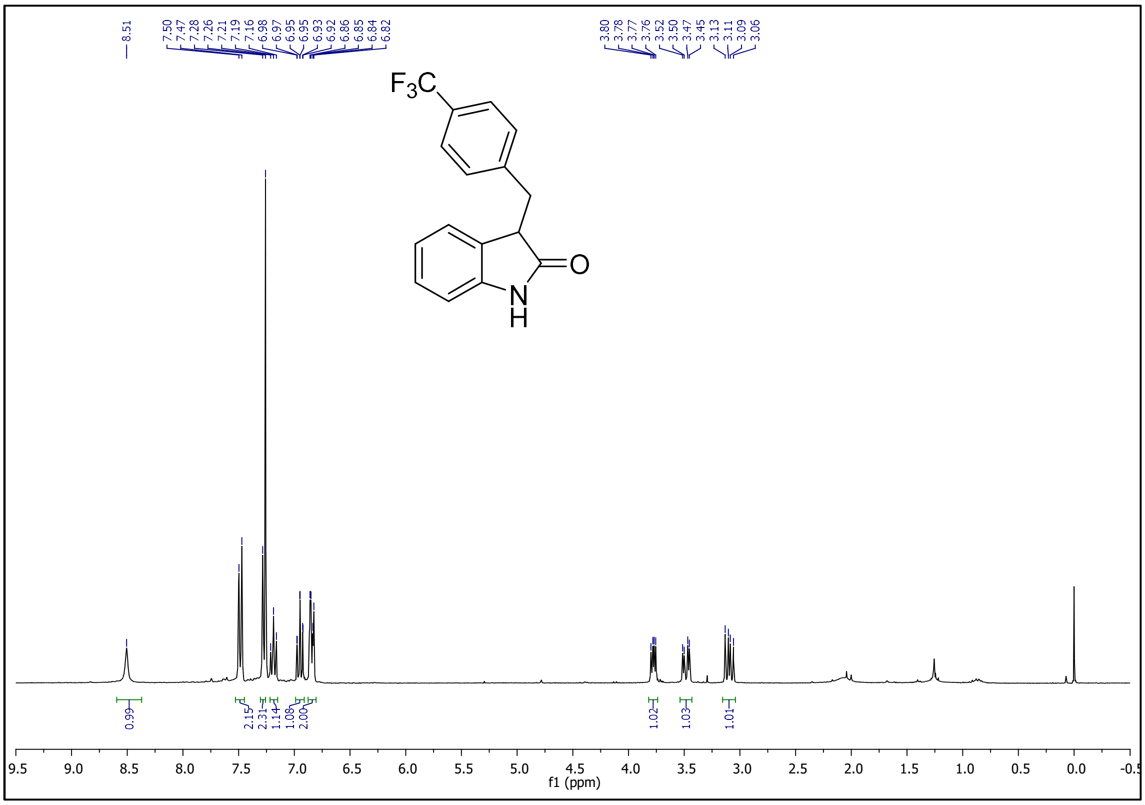
8.51
7.50
7.47
7.28
7.26
7.21
7.19
7.16
6.98
6.97
6.95
6.95
6.93
6.92
6.86
6.85
6.84
6.82
3.80
3.78
3.77
3.76
3.52
3.50
3.47
3.45
3.13
3.11
3.09
3.06
F3C
N
H
O
0.99
2.15
2.31
1.14
1.08
2.00
1.02
1.03
1.01
f1 (ppm)

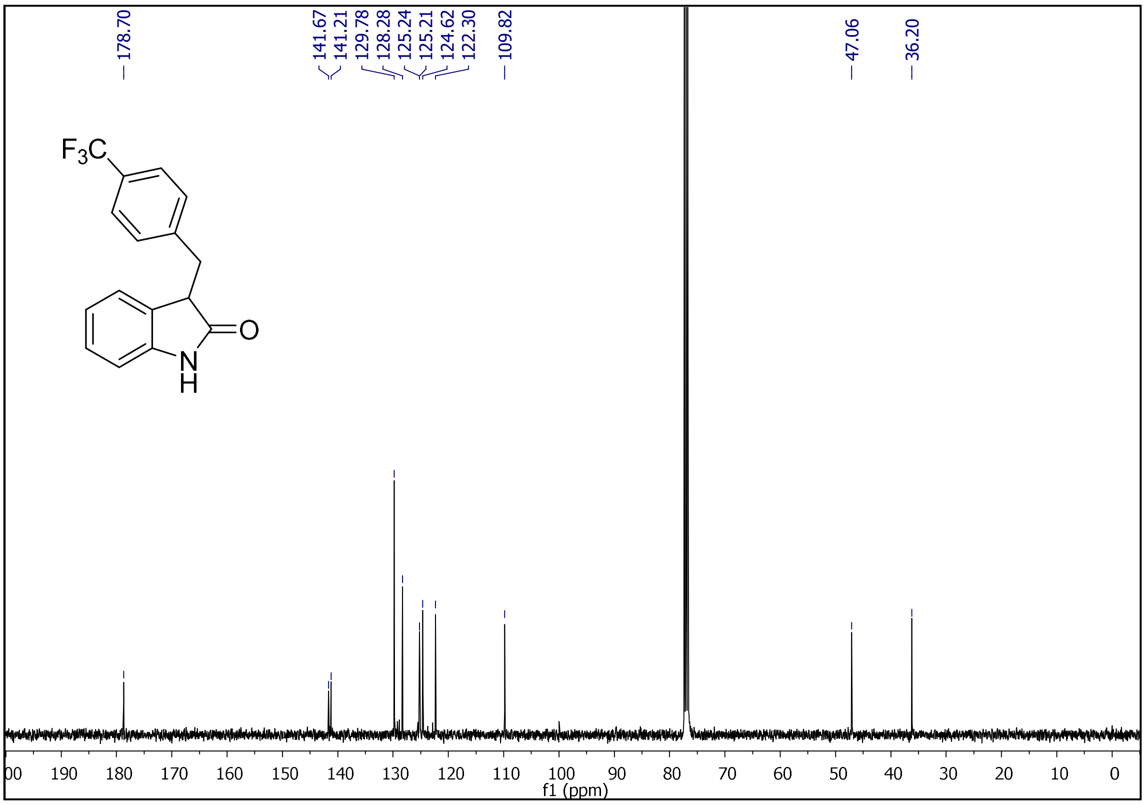
178.70
141.67
141.21
129.78
128.28
125.24
125.21
124.62
122.30
109.82
47.06
36.20
F3C
N
H
O
f1 (ppm)

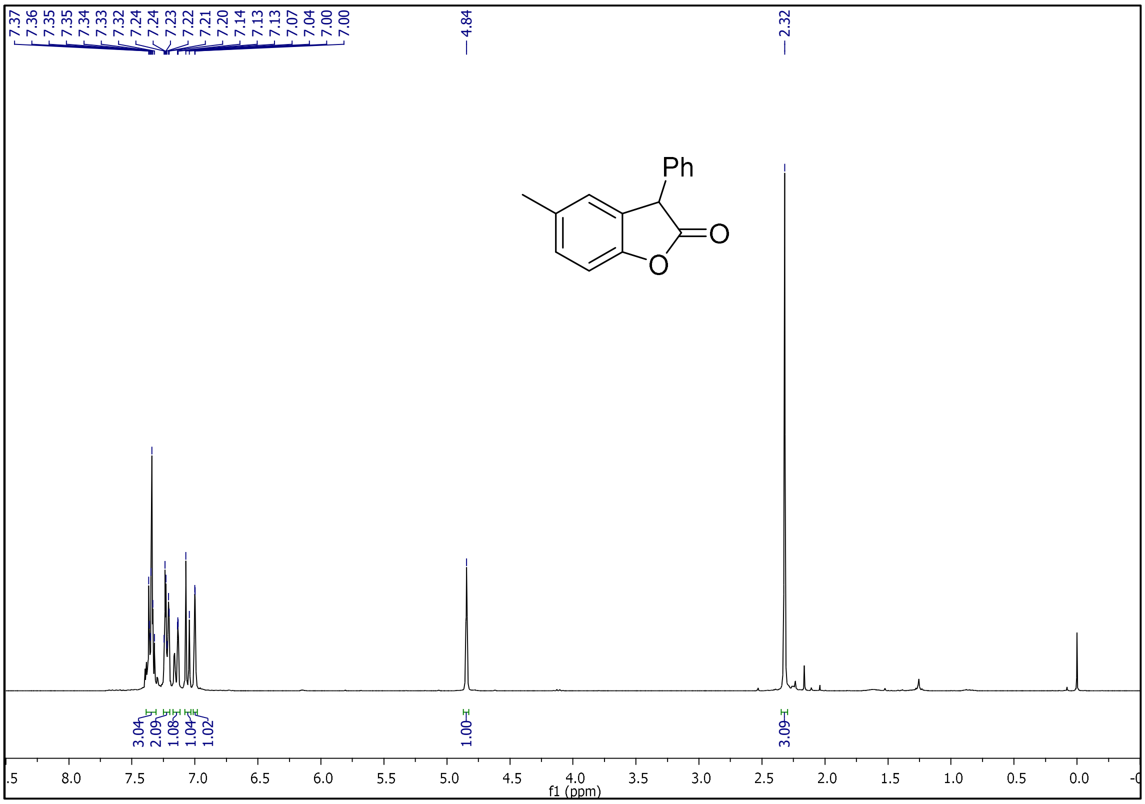
7.37
7.36
7.35
7.35
7.34
7.33
7.32
7.24
7.24
7.23
7.22
7.21
7.20
7.14
7.13
7.13
7.07
7.04
7.00
7.00
4.84
2.32
Ph
O
O
3.04
2.09
1.08
1.04
1.02
1.00
3.09
f1 (ppm)

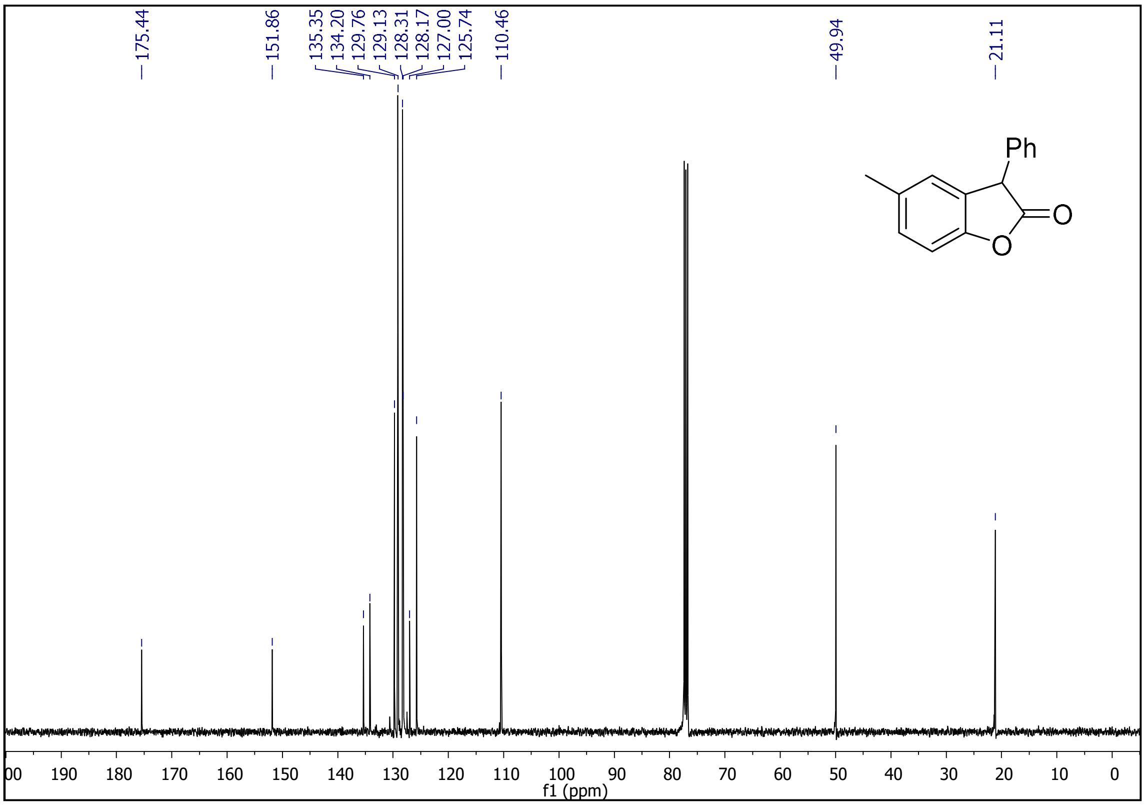
175.44
151.86
135.35
134.20
129.76
129.13
128.31
128.17
127.00
125.74
110.46
49.94
21.11
Ph
O
O
f1 (ppm)

NMR SPECTRA OF AMINATION PRODUCTS

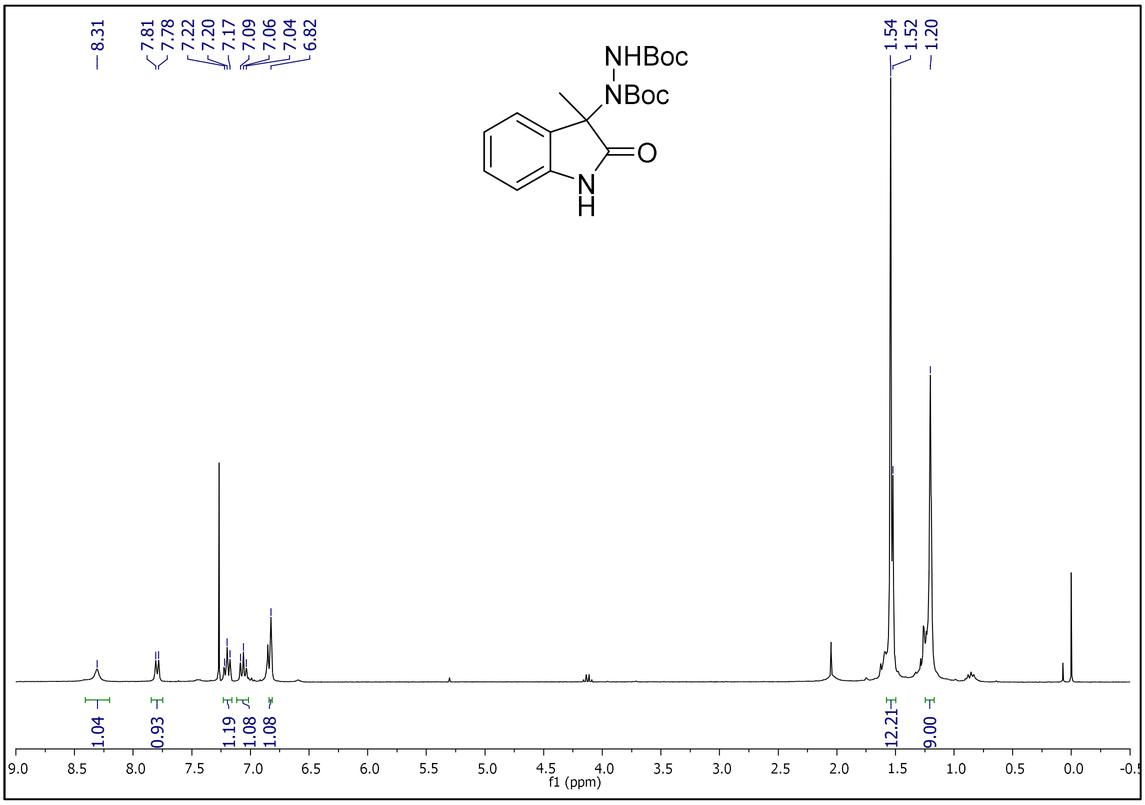

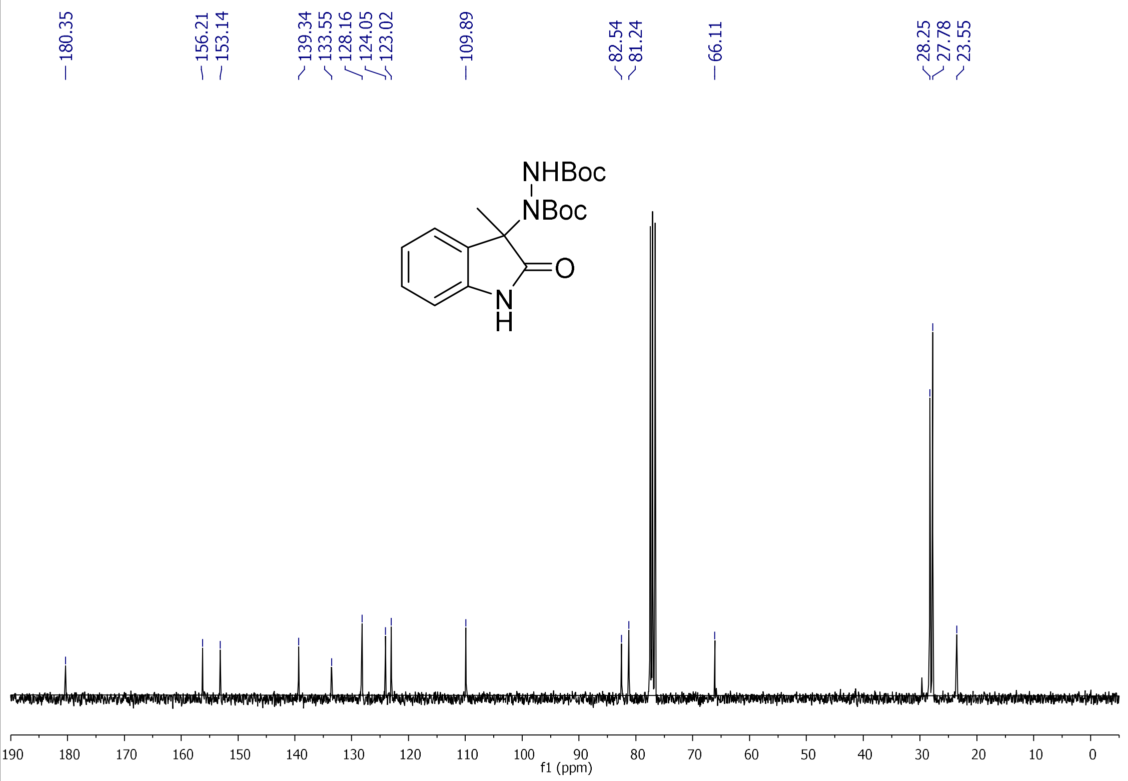

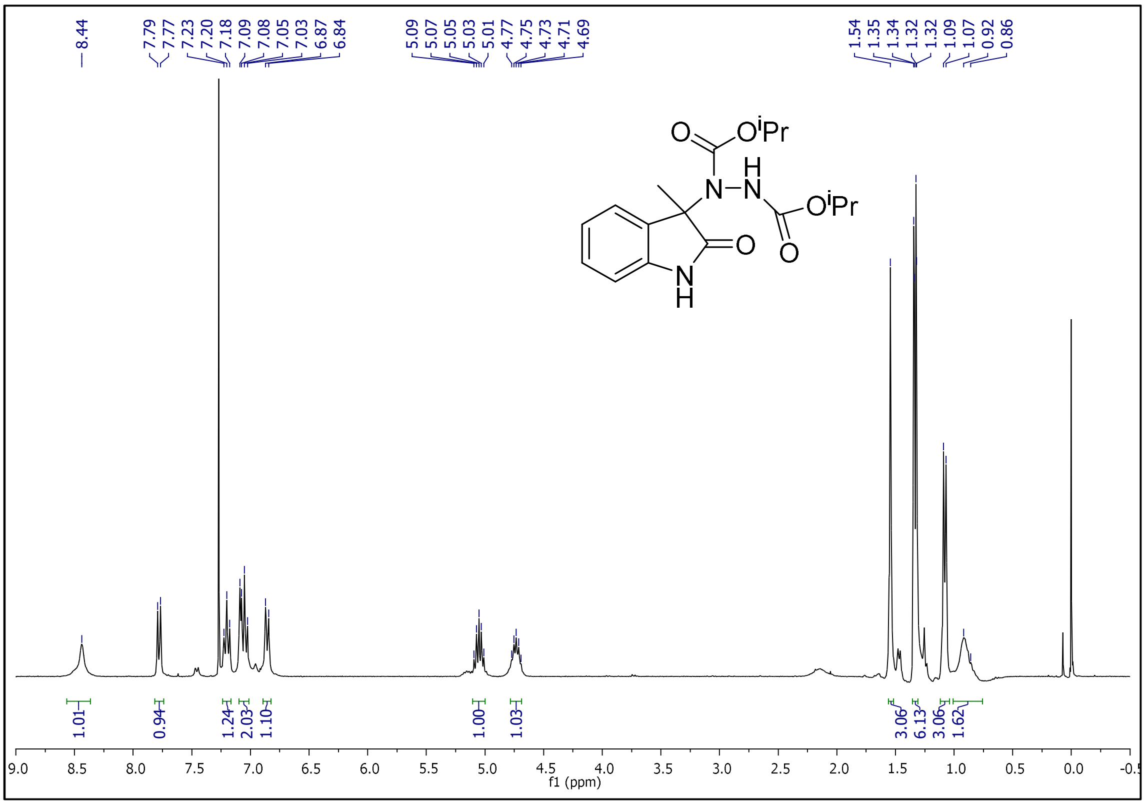
8.44
7.79
7.77
7.23
7.20
7.18
7.09
7.08
7.05
7.03
6.87
6.84
5.09
5.07
5.05
5.03
5.01
4.77
4.75
4.73
4.71
4.69
1.54
1.35
1.34
1.32
1.32
1.09
1.07
0.92
0.86
O
O^{i}Pr
H
N
N
O^{i}Pr
O
O
N
H
1.01
0.94
1.24
2.03
1.10
1.00
1.03
3.06
6.13
3.06
1.62
f1 (ppm)

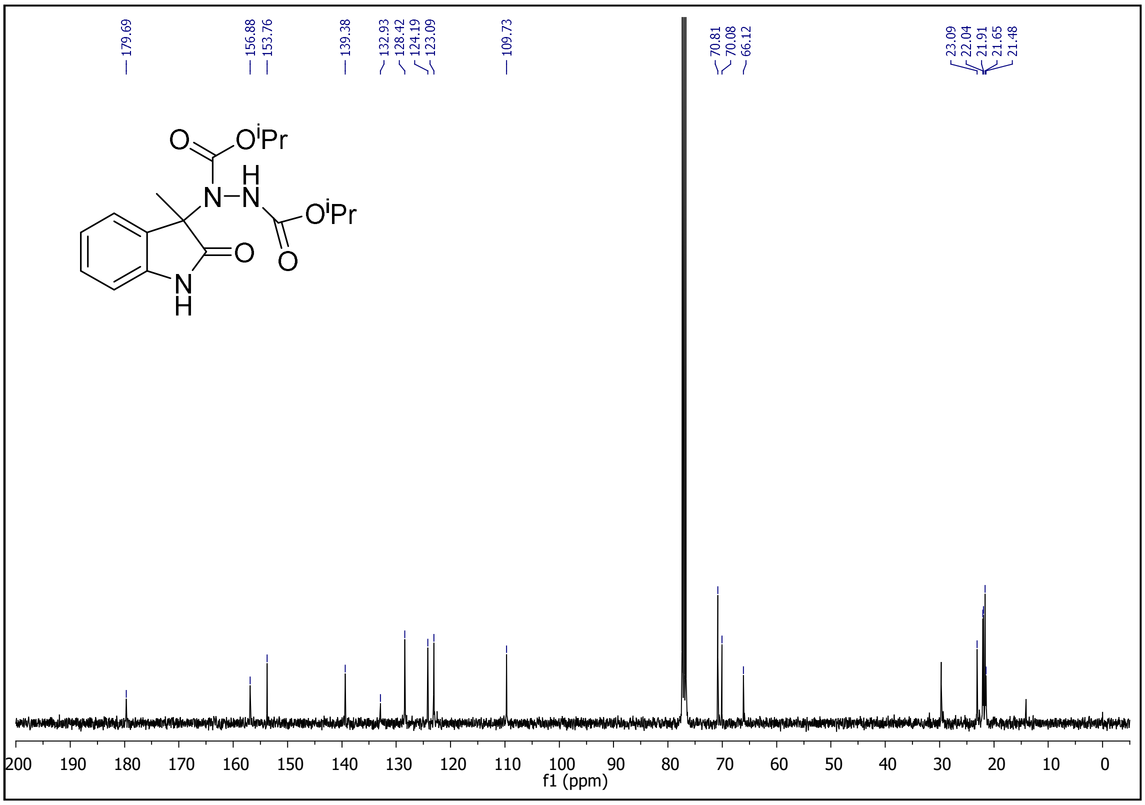
179.69
156.88
153.76
139.38
132.93
128.42
124.19
123.09
109.73
70.81
70.08
66.12
23.09
22.04
21.91
21.65
21.48
O
O^{i}Pr
H
N
N
O^{i}Pr
O
O
N
H
f1 (ppm)

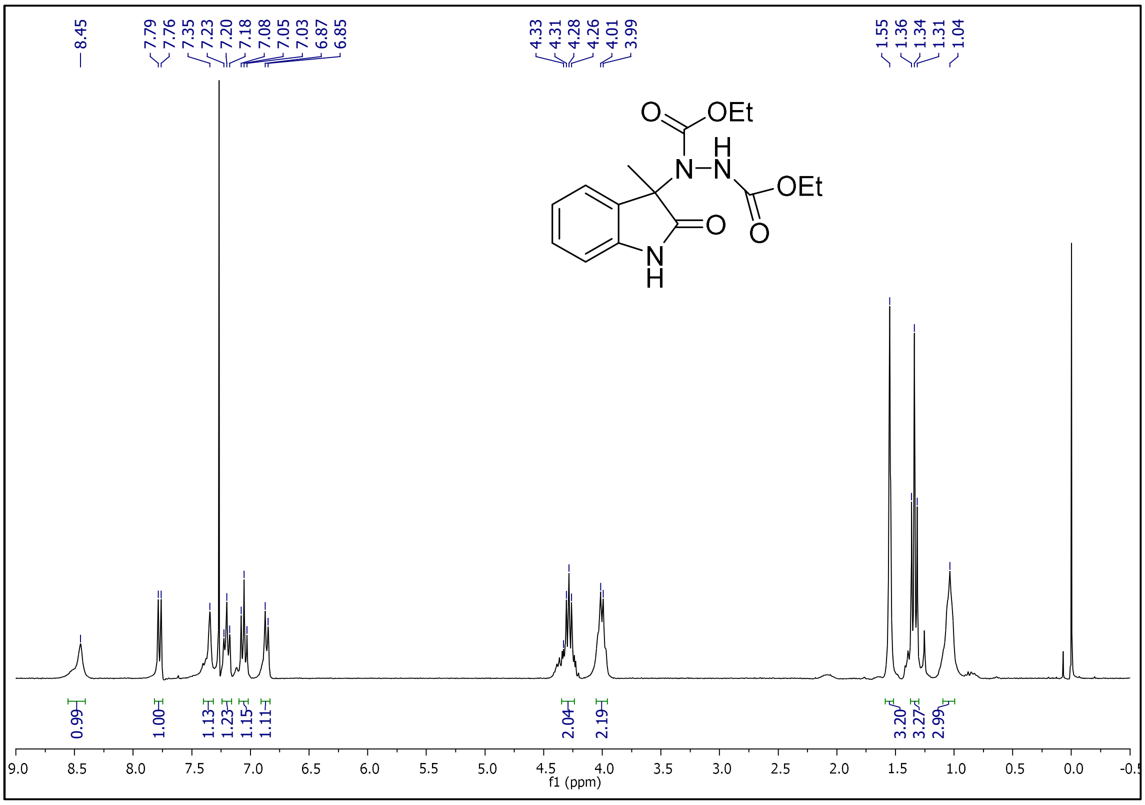
8.45
7.79
7.76
7.35
7.23
7.20
7.18
7.08
7.05
7.03
6.87
6.85
4.33
4.31
4.28
4.26
4.01
3.99
1.55
1.36
1.34
1.31
1.04
OEt
O
H
N
N
OEt
O
O
N
H
0.99
1.00
1.13
1.23
1.15
1.11
2.04
2.19
3.20
3.27
2.99
9.0 8.5 8.0 7.5 7.0 6.5 6.0 5.5 5.0 4.5 4.0 3.5 3.0 2.5 2.0 1.5 1.0 0.5 0.0
f1 (ppm)

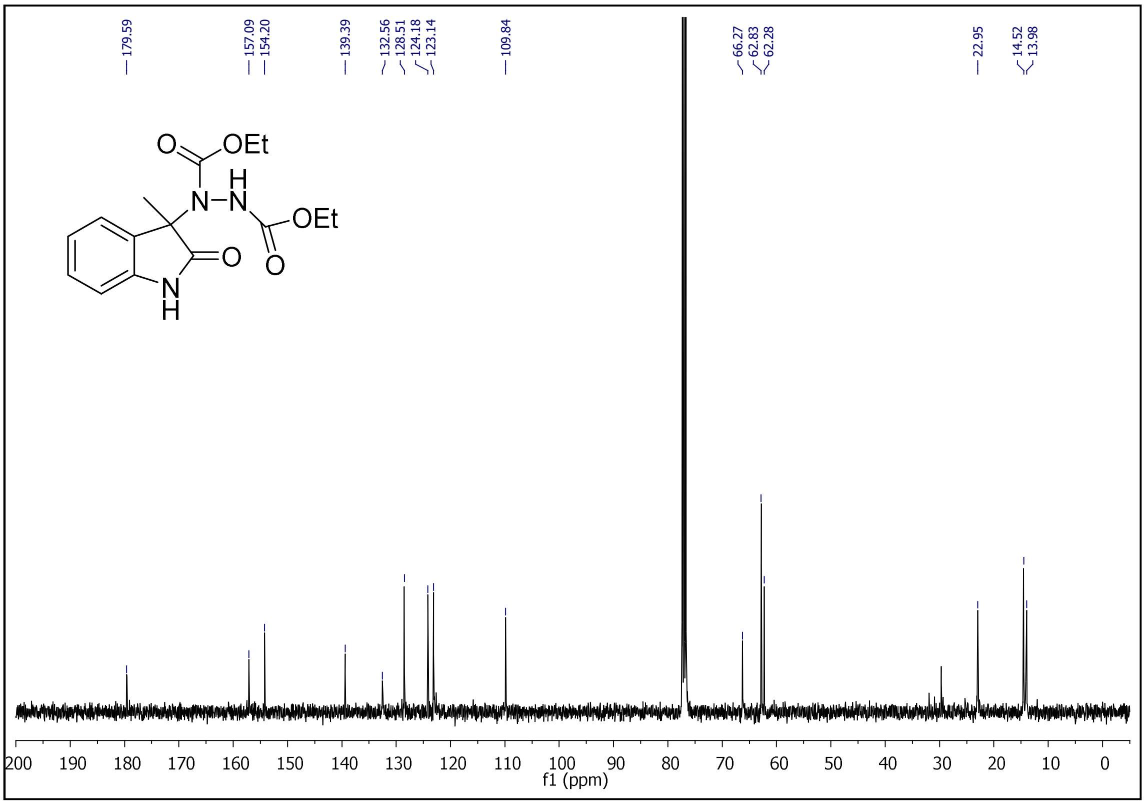
179.59
157.09
154.20
139.39
132.56
128.51
124.18
123.14
109.84
66.27
62.83
62.28
22.95
14.52
13.98
OEt
O
H
N
N
OEt
O
O
N
H
200 190 180 170 160 150 140 130 120 110 100 90 80 70 60 50 40 30 20 10 0
f1 (ppm)

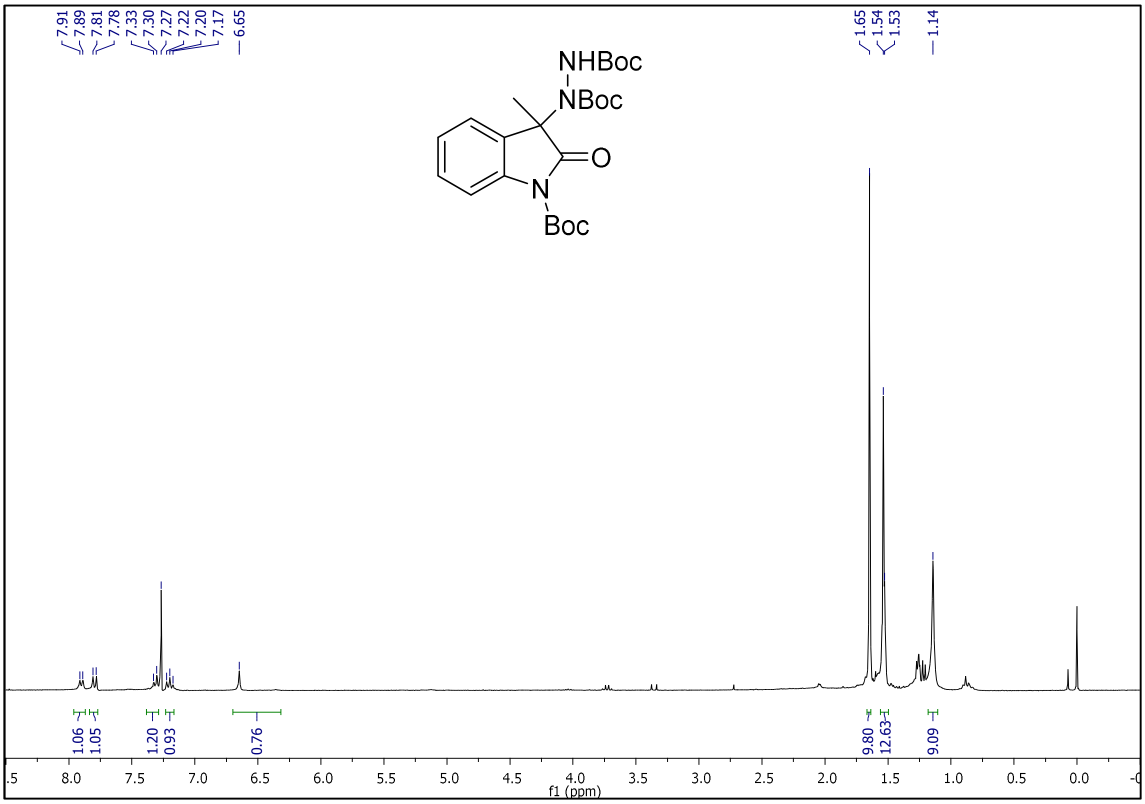
7.91
7.89
7.81
7.78
7.33
7.30
7.27
7.22
7.20
7.17
6.65
1.65
1.54
1.53
1.14
NHBoc
NBoc
O
N
Boc
1.06
1.05
1.20
0.93
0.76
9.80
12.63
9.09
f1 (ppm)

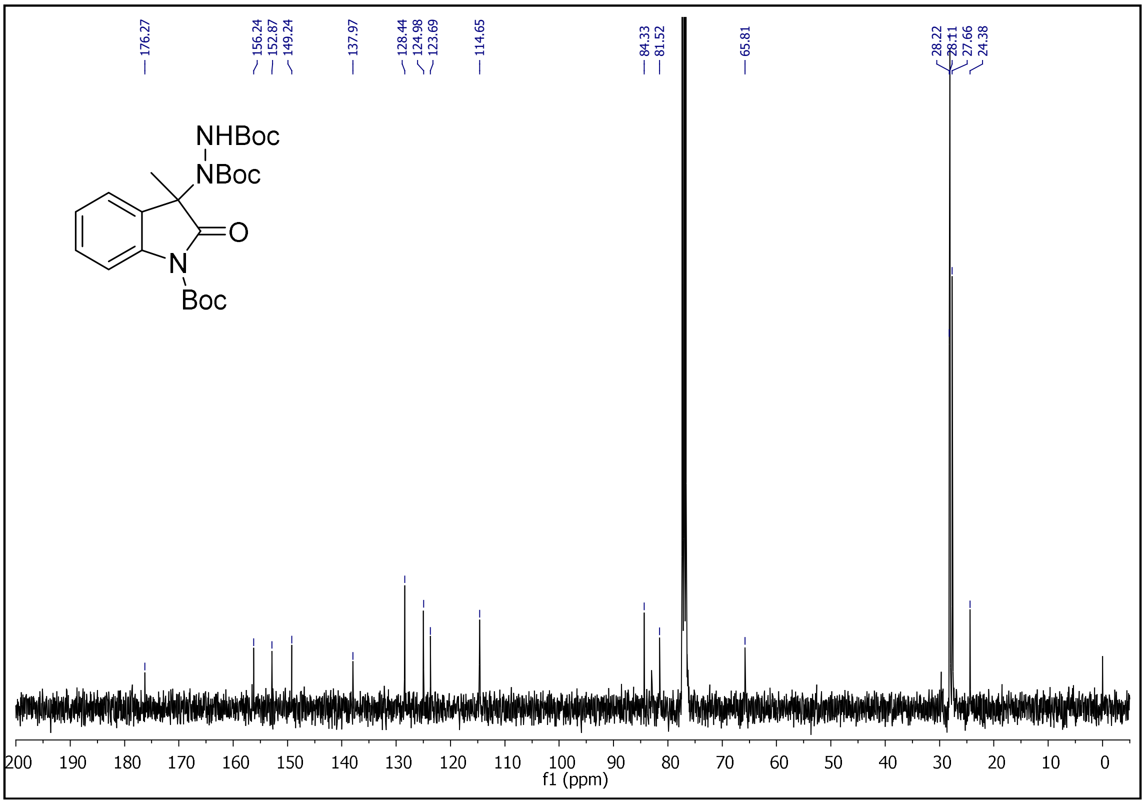
176.27
156.24
152.87
149.24
137.97
128.44
124.98
123.69
114.65
84.33
81.52
65.81
28.22
28.11
27.66
24.38
NHBoc
NBoc
O
N
Boc
f1 (ppm)

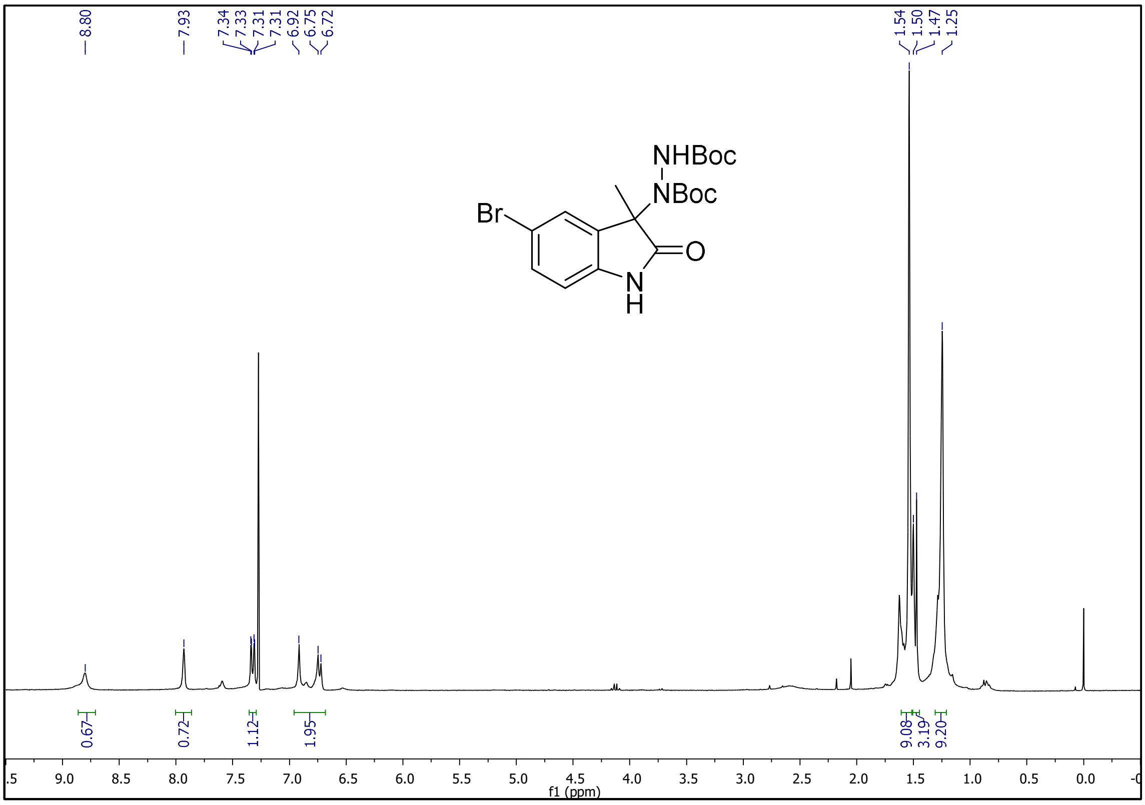
8.80
7.93
7.34
7.33
7.31
7.31
6.92
6.75
6.72
1.54
1.50
1.47
1.25
NHBoc
NBoc
Br
O
N
H
0.67
0.72
1.12
1.95
9.08
3.19
9.20
f1 (ppm)

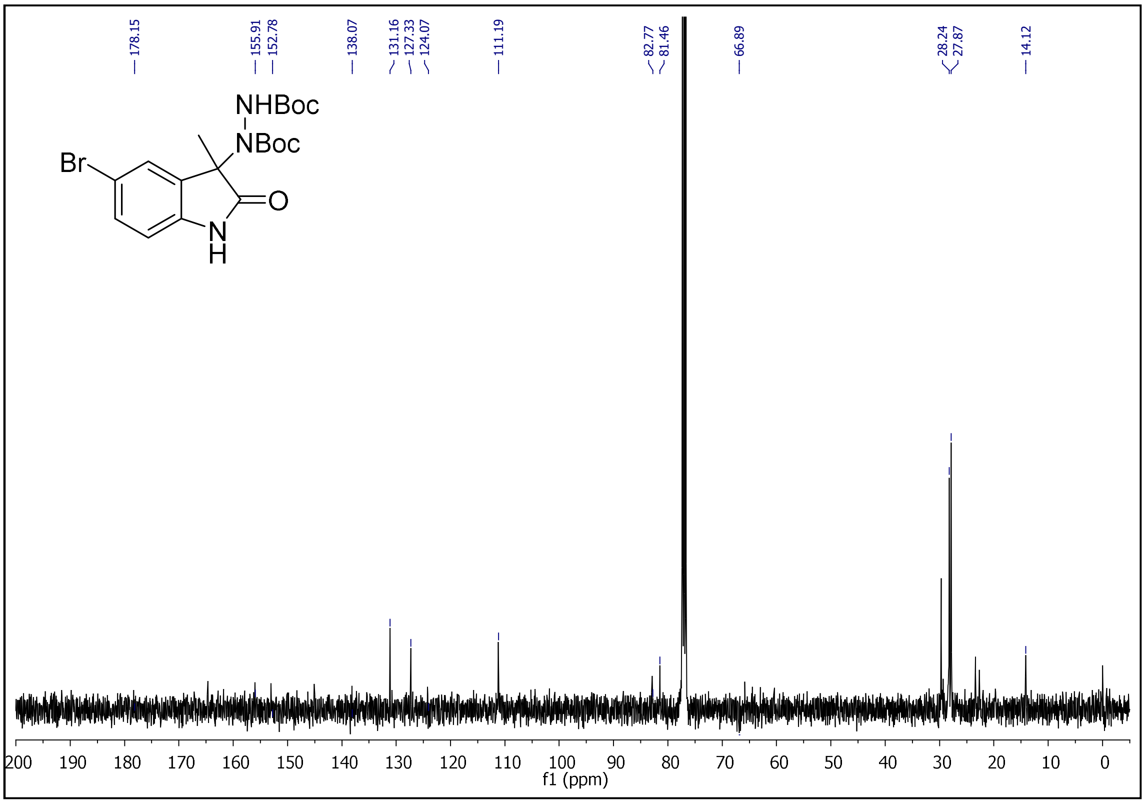
178.15
155.91
152.78
138.07
131.16
127.33
124.07
111.19
82.77
81.46
66.89
28.24
27.87
14.12
NHBoc
NBoc
Br
O
N
H
f1 (ppm)

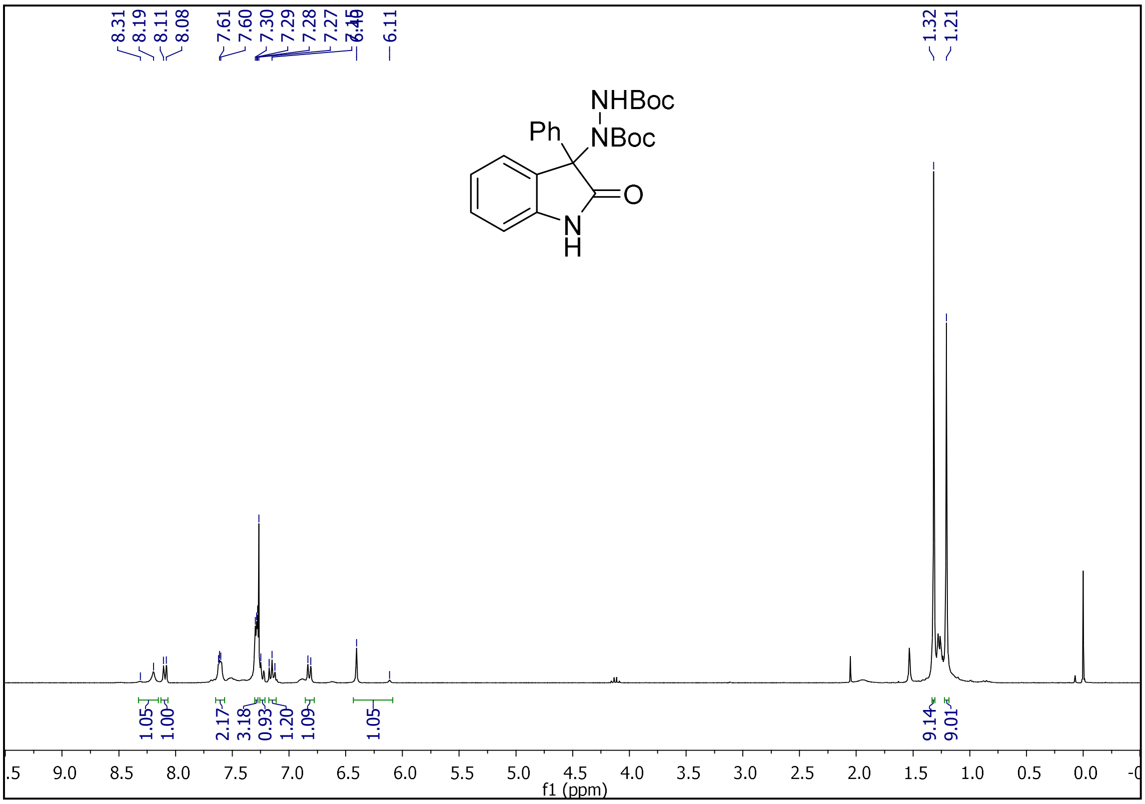

8.31
8.19
8.11
8.08
7.61
7.60
7.30
7.29
7.28
7.27
7.15
6.40
6.11
1.32
1.21
NHBoc
Ph
NBoc
O
N
H
1.05
1.00
2.17
3.18
0.93
1.20
1.09
1.05
9.14
9.01
.5 9.0 8.5 8.0 7.5 7.0 6.5 6.0 5.5 5.0 4.5 4.0 3.5 3.0 2.5 2.0 1.5 1.0 0.5 0.0
f1 (ppm)

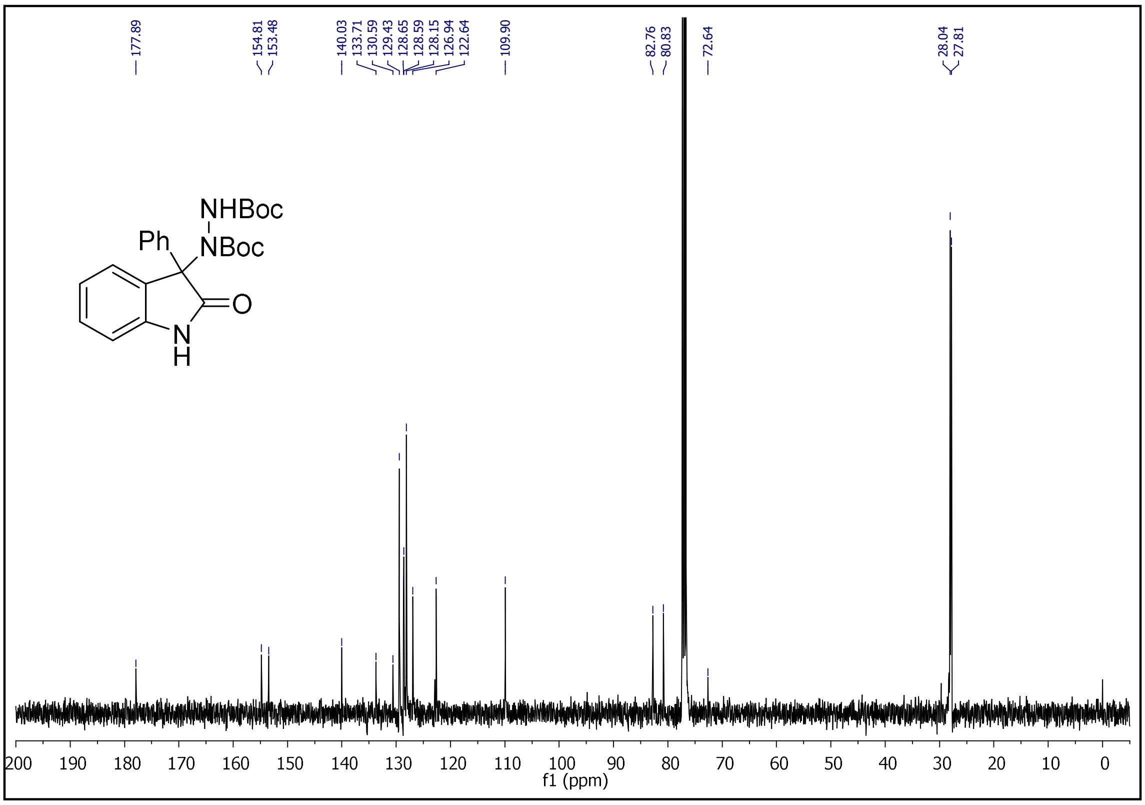

177.89
154.81
153.48
140.03
133.71
130.59
129.43
128.65
128.59
128.15
126.94
122.64
109.90
82.76
80.83
72.64
28.04
27.81
NHBoc
Ph
NBoc
O
N
H
200 190 180 170 160 150 140 130 120 110 100 90 80 70 60 50 40 30 20 10 0
f1 (ppm)

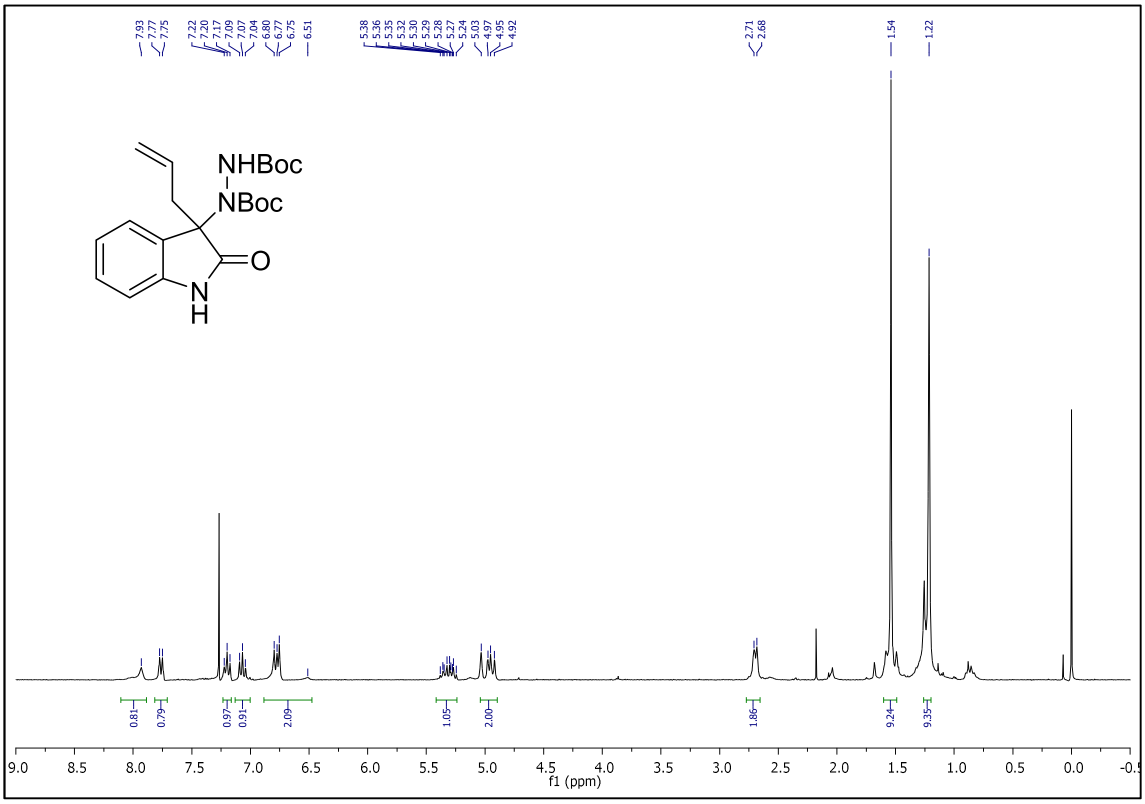
NHBoc
NBoc
O
N
H
f1 (ppm)

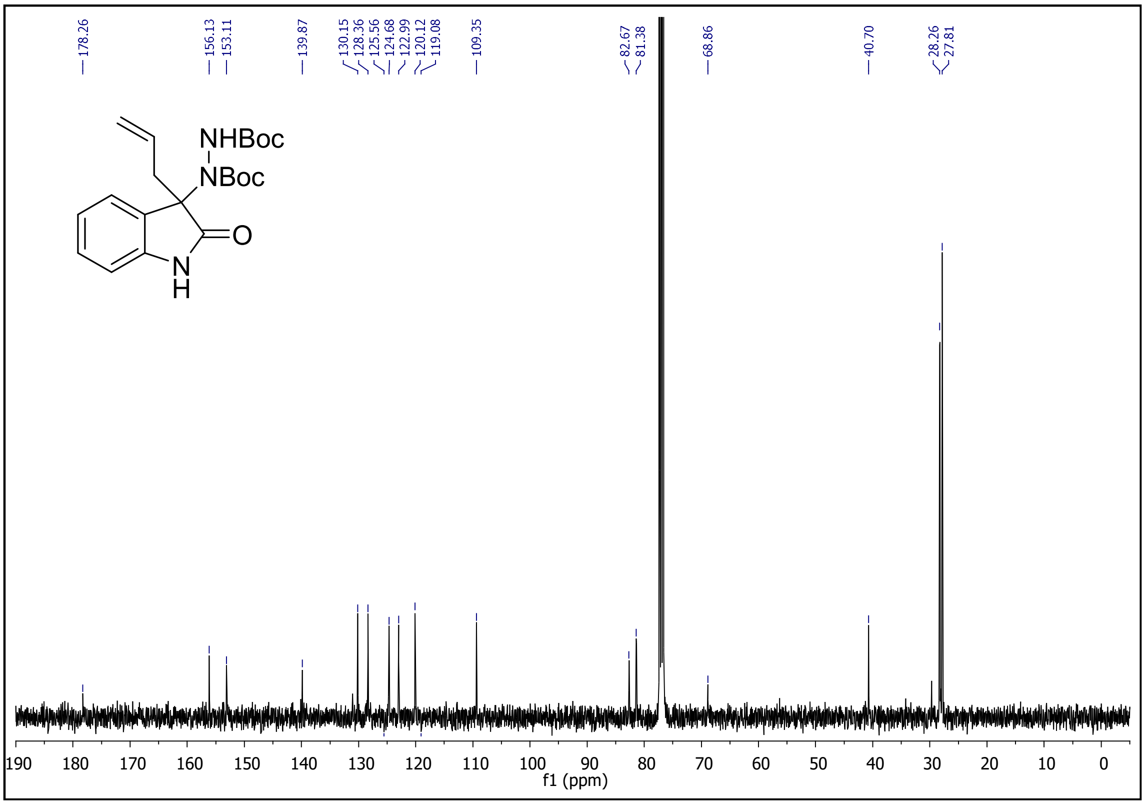
NHBoc
NBoc
O
N
H
f1 (ppm)

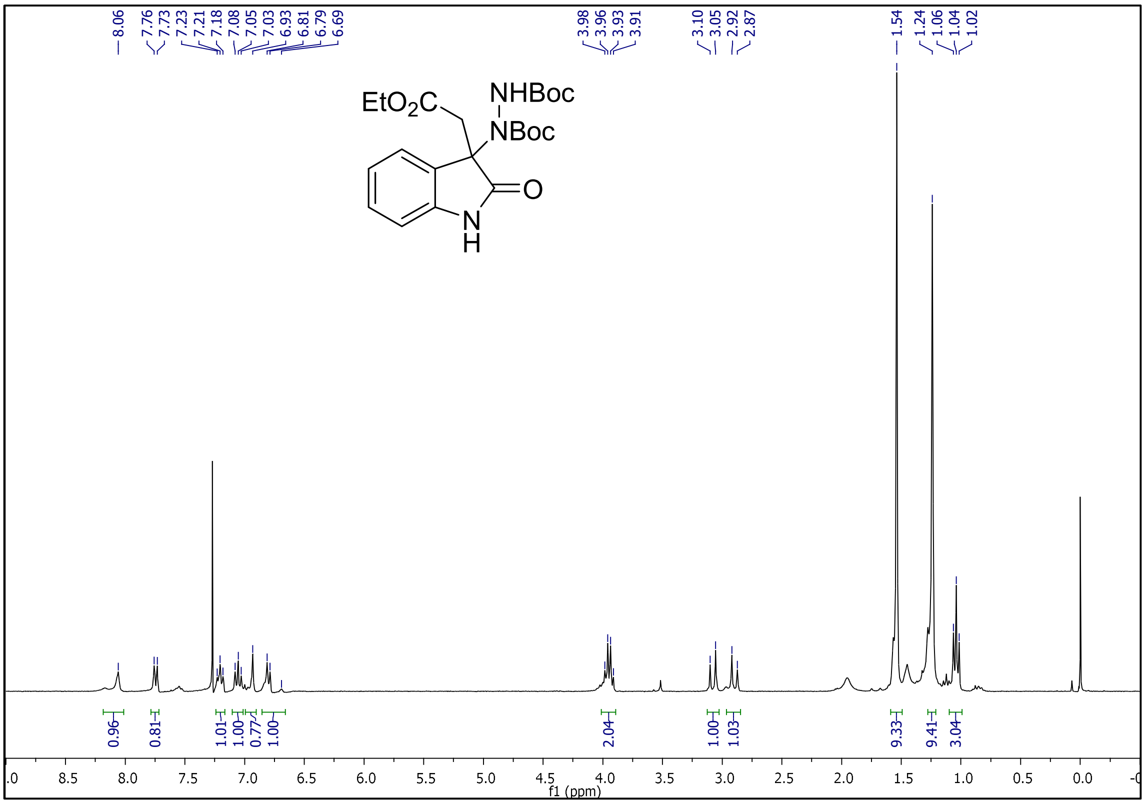
8.06
7.76
7.73
7.23
7.21
7.18
7.08
7.05
7.03
6.93
6.81
6.79
6.69
3.98
3.96
3.93
3.91
3.10
3.05
2.92
2.87
1.54
1.24
1.06
1.04
1.02
EtO2C
NHBoc
NBoc
O
N
H
0.96
0.81
1.01
1.00
0.77
1.00
2.04
1.00
1.03
9.33
9.41
3.04
f1 (ppm)

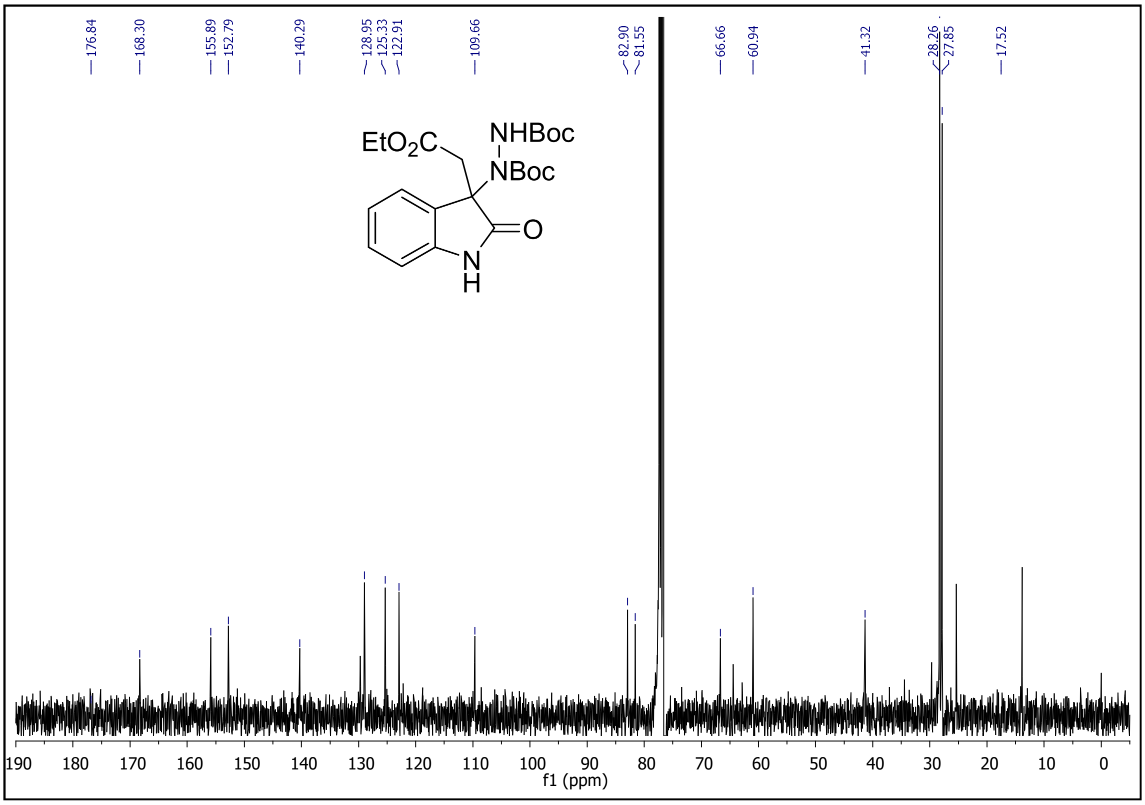
176.84
168.30
155.89
152.79
140.29
128.95
125.33
122.91
109.66
82.90
81.55
66.66
60.94
41.32
28.26
27.85
17.52
EtO2C
NHBoc
NBoc
O
N
H
f1 (ppm)

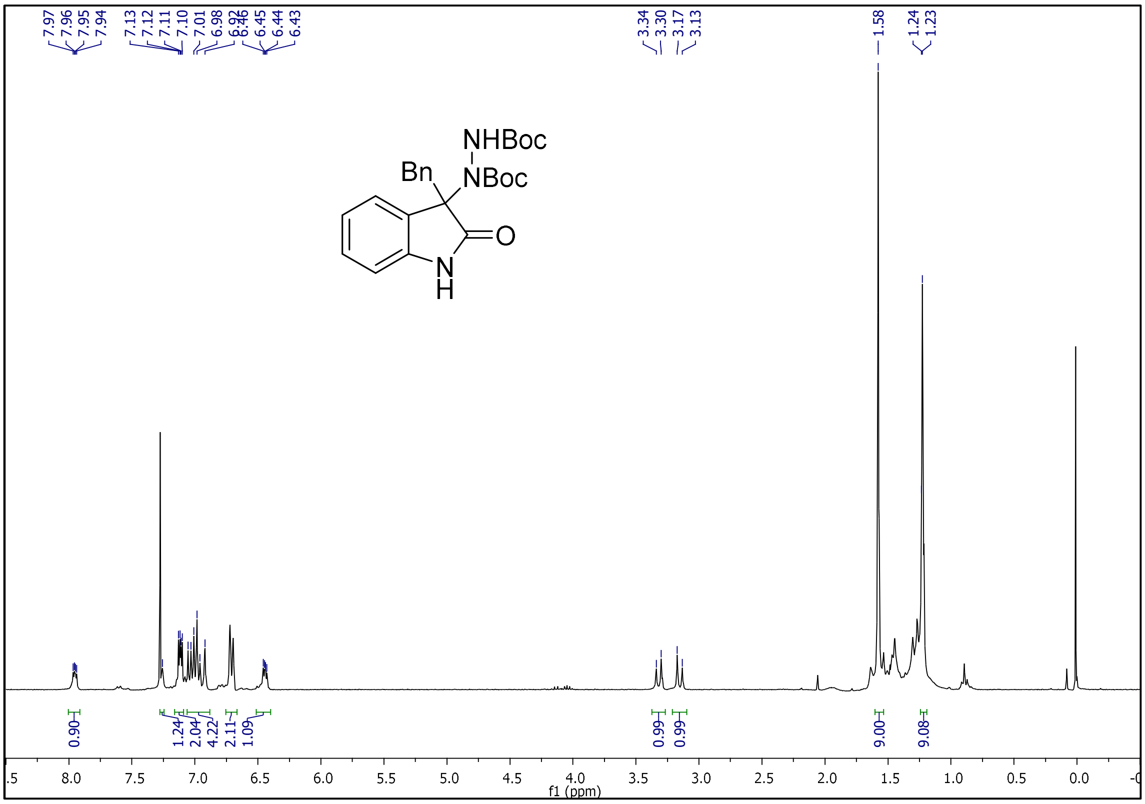

7.97
7.96
7.95
7.94
7.13
7.12
7.11
7.10
7.01
6.98
6.45
6.44
6.43
3.34
3.30
3.17
3.13
1.58
1.24
1.23
NHBoc
Bn
NBoc
O
N
H
0.90
1.24
2.04
4.22
2.11
1.09
0.99
0.99
9.00
9.08
8.0 7.5 7.0 6.5 6.0 5.5 5.0 4.5 4.0 3.5 3.0 2.5 2.0 1.5 1.0 0.5 0.0
f1 (ppm)

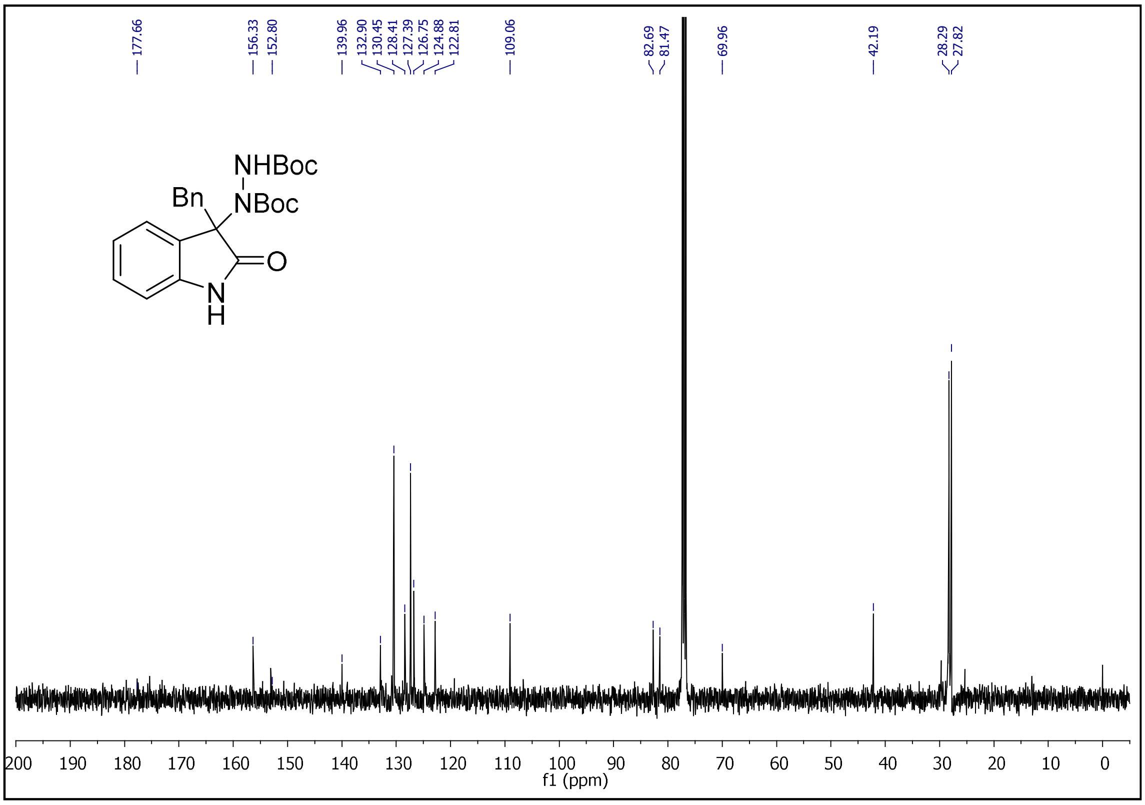

177.66
156.33
152.80
139.96
132.90
130.45
128.41
127.39
126.75
124.88
122.81
109.06
82.69
81.47
69.96
42.19
28.29
27.82
NHBoc
Bn
NBoc
O
N
H
200 190 180 170 160 150 140 130 120 110 100 90 80 70 60 50 40 30 20 10 0
f1 (ppm)

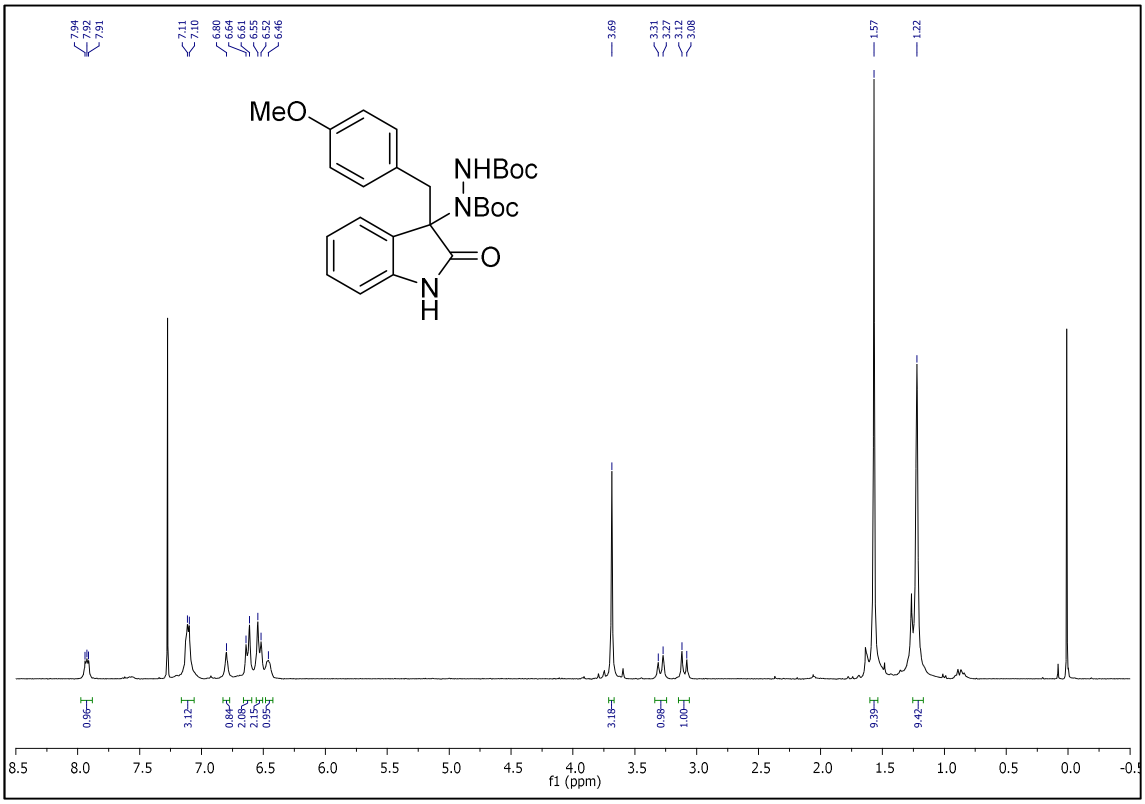
MeO
NHBoc
NBoc
O
N
H
f1 (ppm)

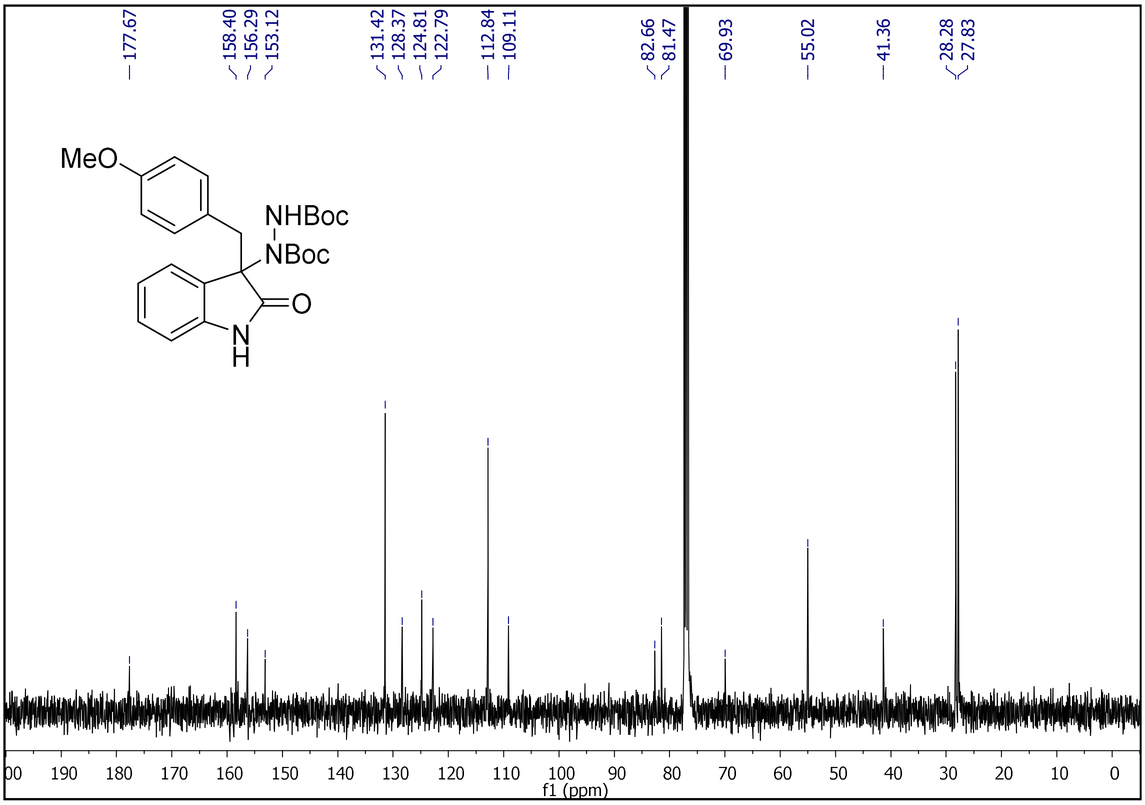
177.67
158.40
156.29
153.12
131.42
128.37
124.81
122.79
112.84
109.11
82.66
81.47
69.93
55.02
41.36
28.28
27.83
MeO
NHBoc
NBoc
O
N
H
f1 (ppm)

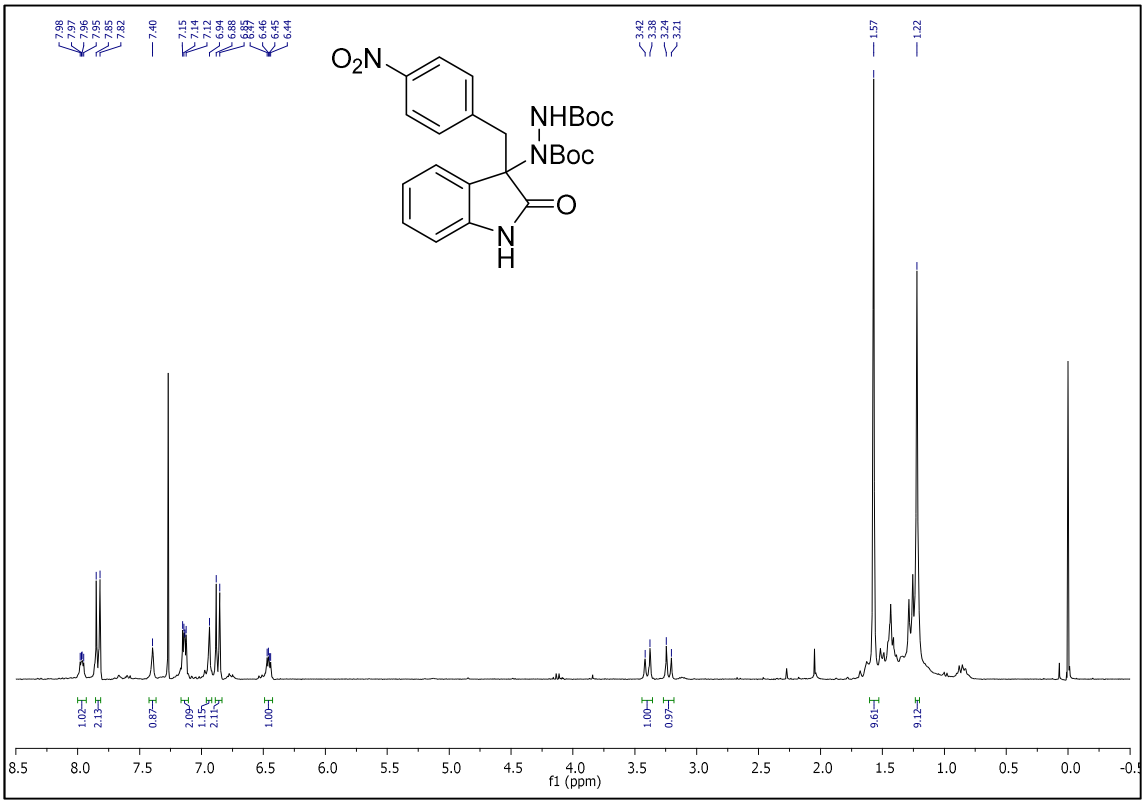
7.98
7.97
7.96
7.95
7.85
7.82
7.40
7.15
7.14
7.12
6.94
6.88
6.85
6.47
6.46
6.45
6.44
3.42
3.38
3.24
3.21
1.57
1.22
O_2N
NHBoc
NBoc
O
N
H
1.02
2.13
0.87
2.09
1.15
2.11
1.00
1.00
0.97
9.61
9.12
f1 (ppm)

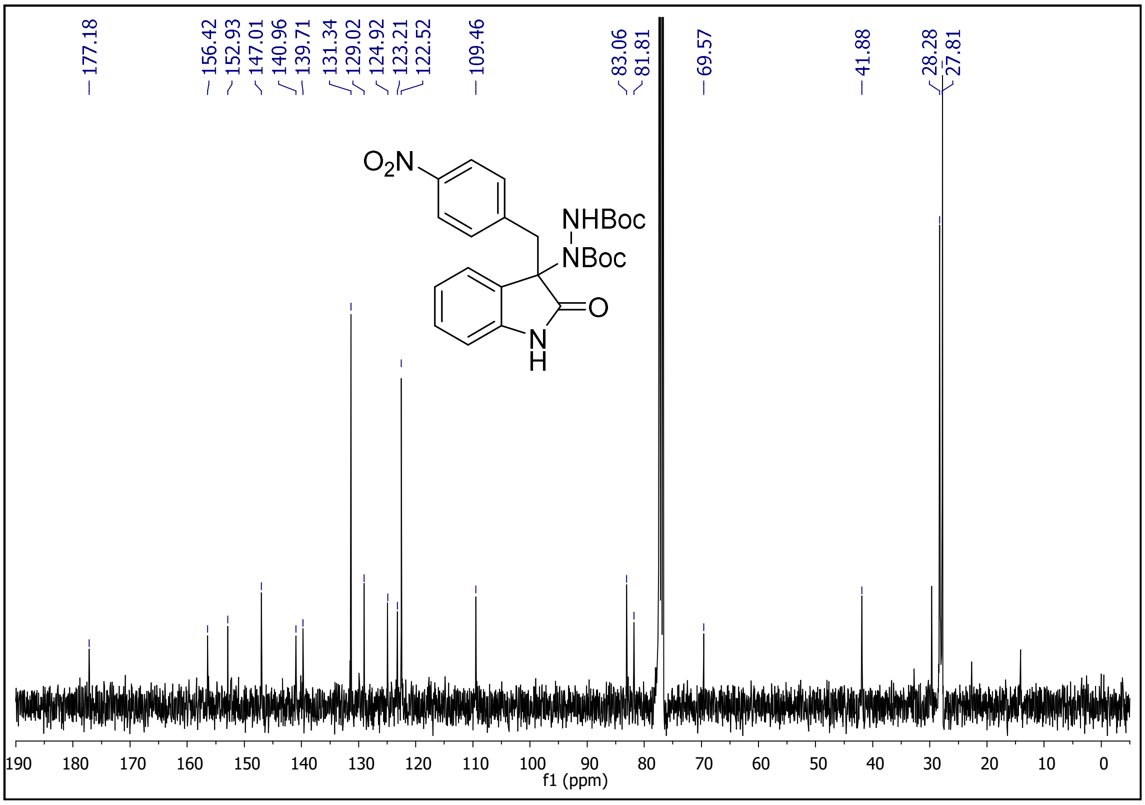
177.18
156.42
152.93
147.01
140.96
139.71
131.34
129.02
124.92
123.21
122.52
109.46
83.06
81.81
69.57
41.88
28.28
27.81
O_2N
NHBoc
NBoc
O
N
H
f1 (ppm)

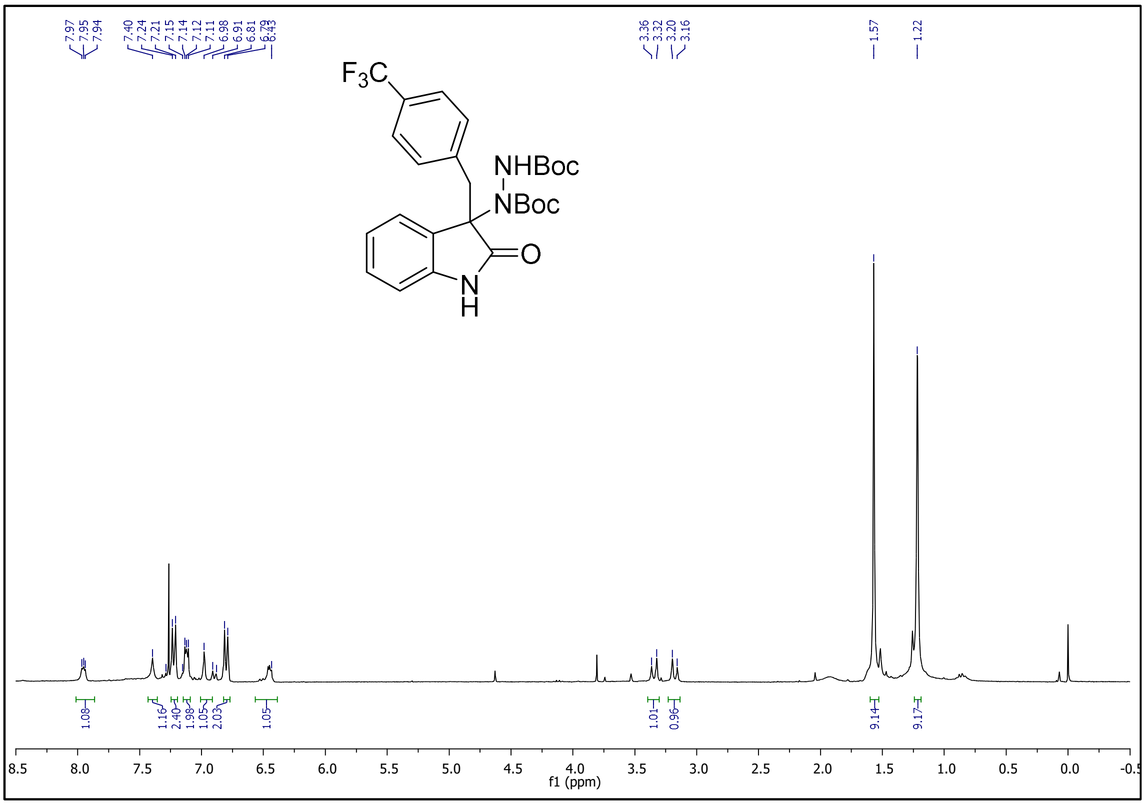

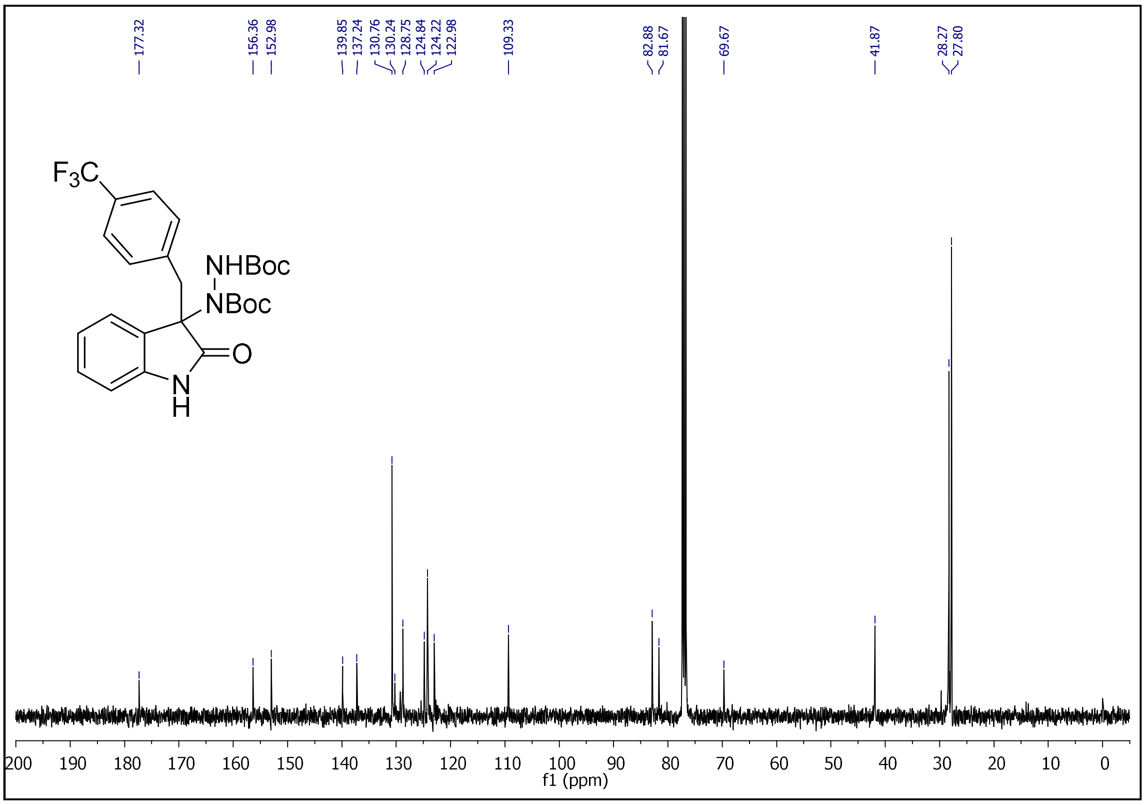

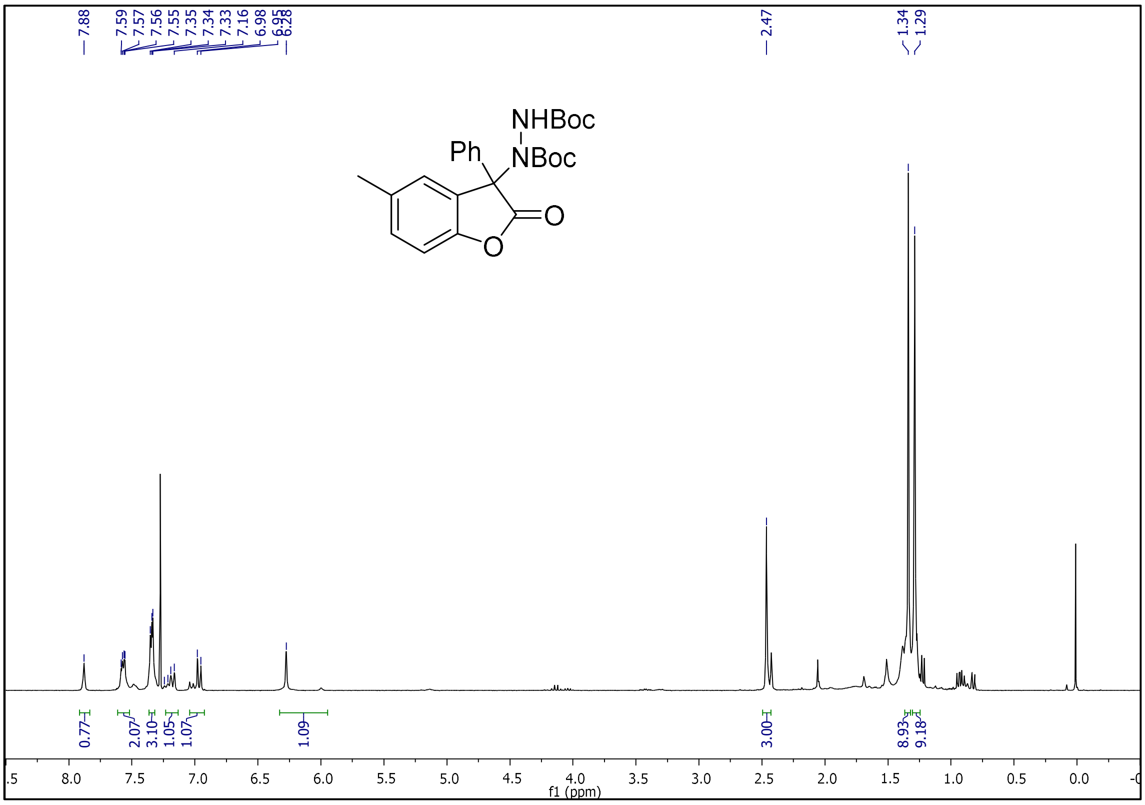
7.88
7.59
7.57
7.56
7.55
7.35
7.34
7.33
7.16
6.98
6.95
6.28
2.47
1.34
1.29
NHBoc
Ph
NBoc
O
O
0.77
2.07
3.10
1.05
1.07
1.09
3.00
8.93
9.18
f1 (ppm)

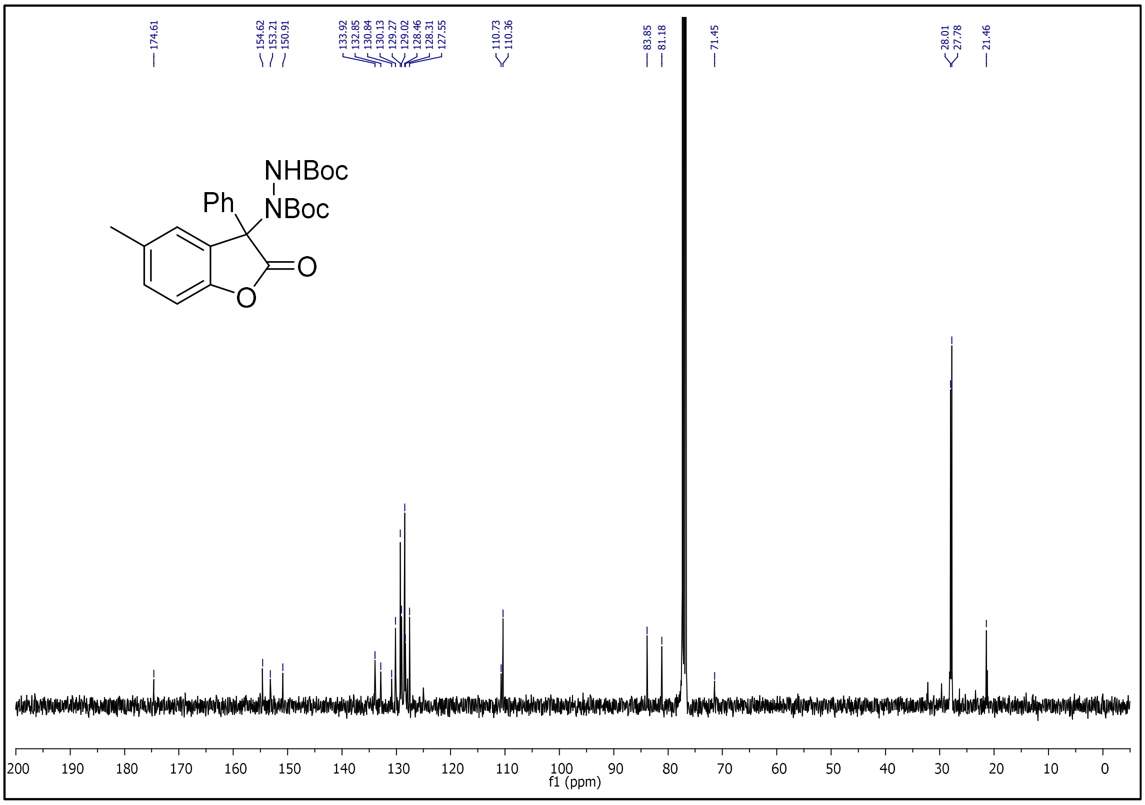
174.61
154.62
153.21
150.91
133.92
132.85
130.84
130.13
129.27
129.02
128.46
128.31
127.55
110.73
110.36
83.85
81.18
71.45
28.01
27.78
21.46
NHBoc
Ph
NBoc
O
O
f1 (ppm)

HPLC CHROMATOGRAM OF THE AMINATION PRODUCTS

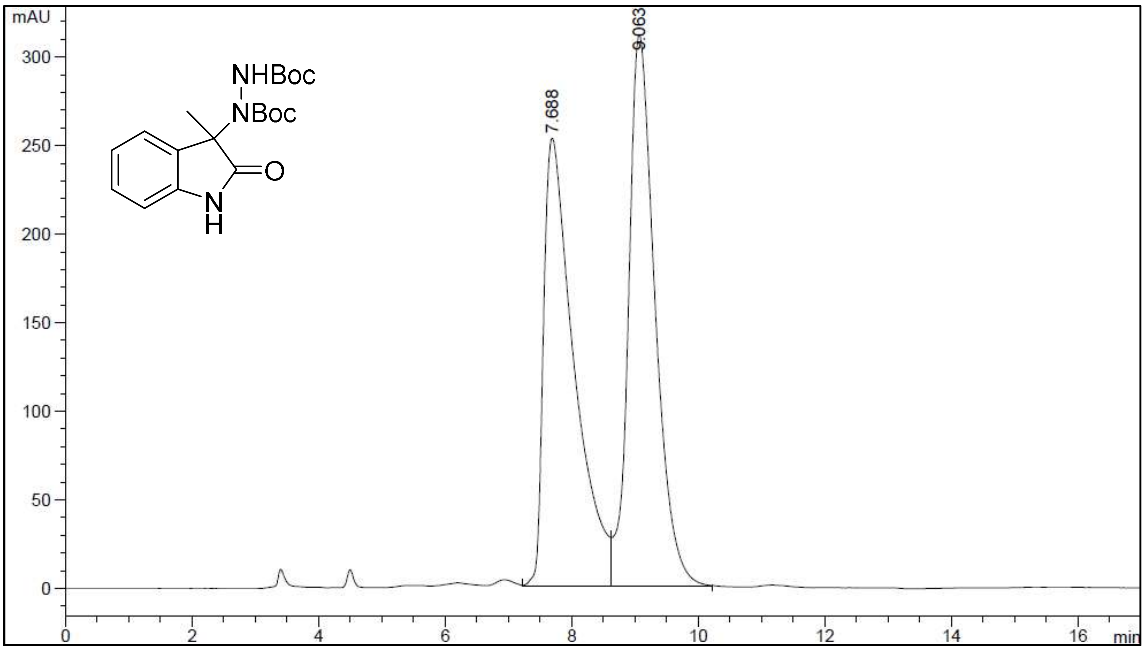

Peak	Ret. Time (min)	Type	Width	Area (mAU*s)	Height (mAU)	Area (%)
1	7.688	VV	0.4929	8478.5654	253.0273	48.3234
2	9.063	VB	0.4416	9066.8945	311.4492	51.6766

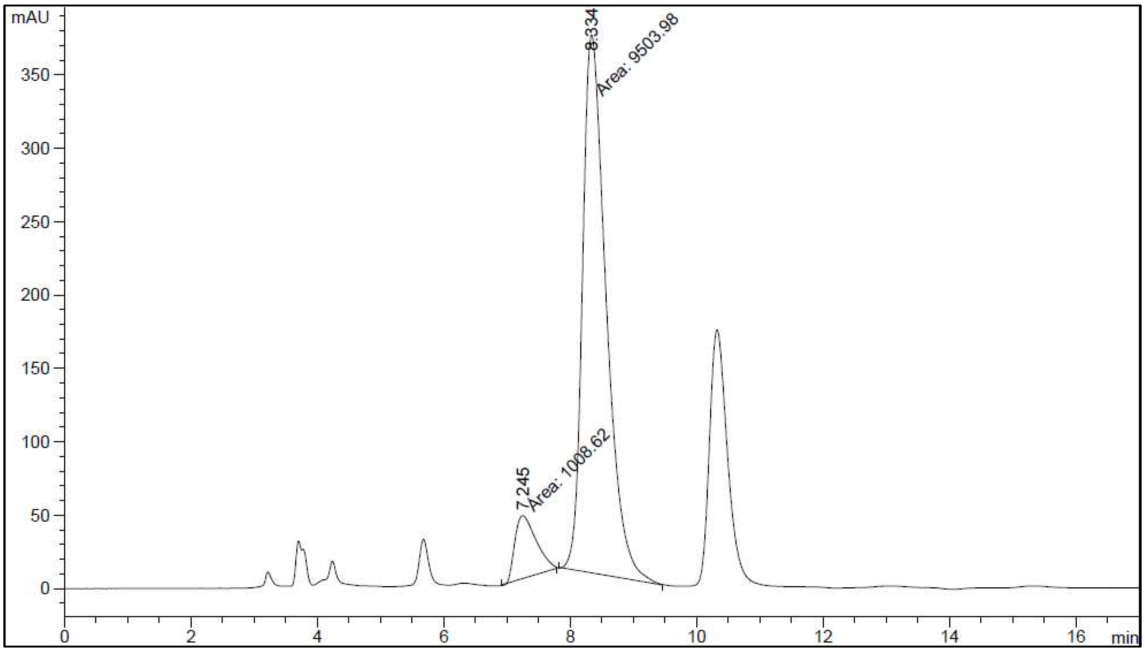

Peak	Ret. Time (min)	Type	Width	Area (mAU*s)	Height (mAU)	Area (%)
1	7245	MM	0.3905	1008.6191	43.0523	9.5944
2	8.334	MM	0.4321	9503.9466	366.5789	90.4056

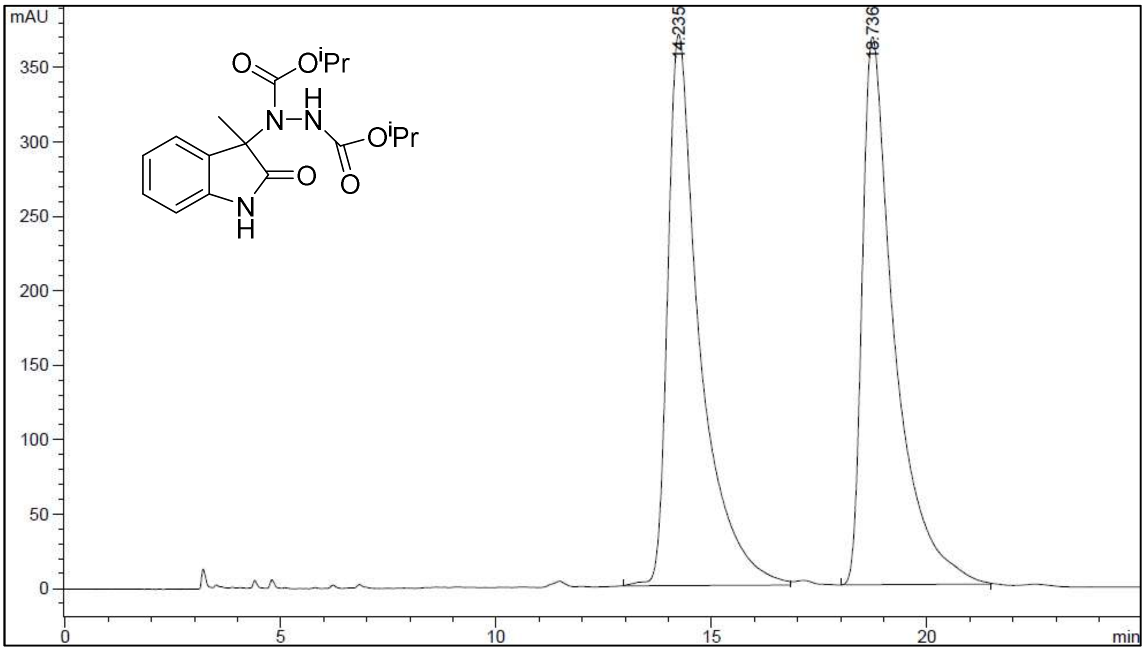

Peak	Ret. Time (min)	Type	Width	Area (mAU*s)	Height (mAU)	Area (%)
1	14.235	BB	0.7498	19244.3	370.6246	50.0934
2	18.736	BB	0.7538	19172.5	368.0325	49.9066

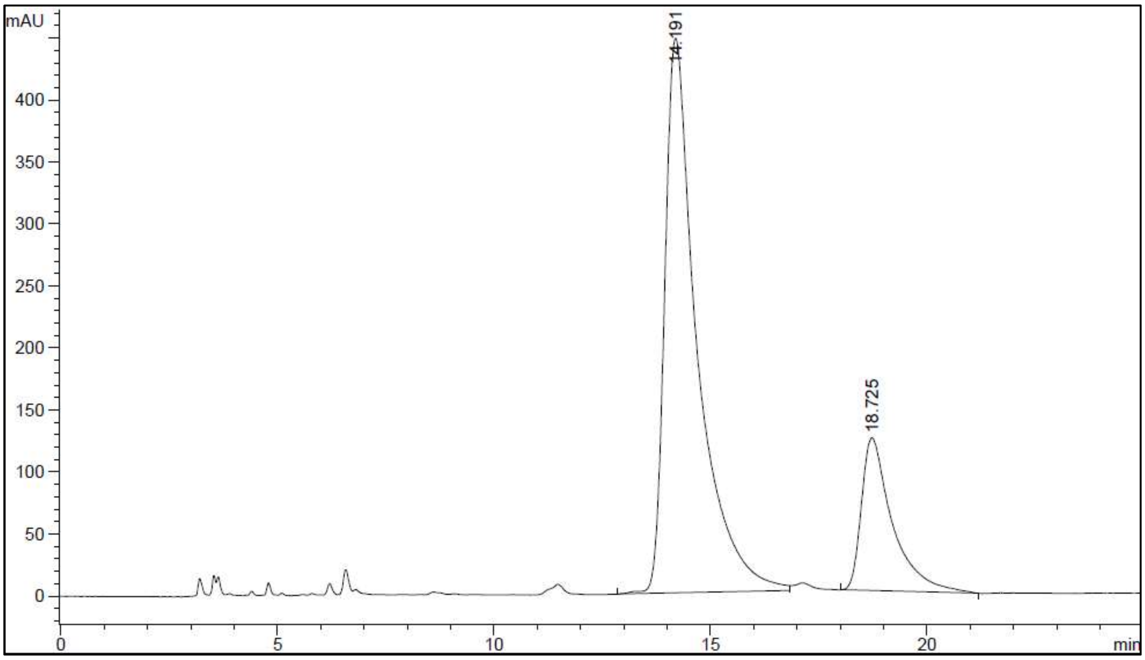

Peak	Ret. Time (min)	Type	Width	Area (mAU*s)	Height (mAU)	Area (%)
1	14.191	BB	0.7561	23308.6	447.2088	79.1867
2	18.725	BP	0.7102	6162.39	123.1996	20.8133

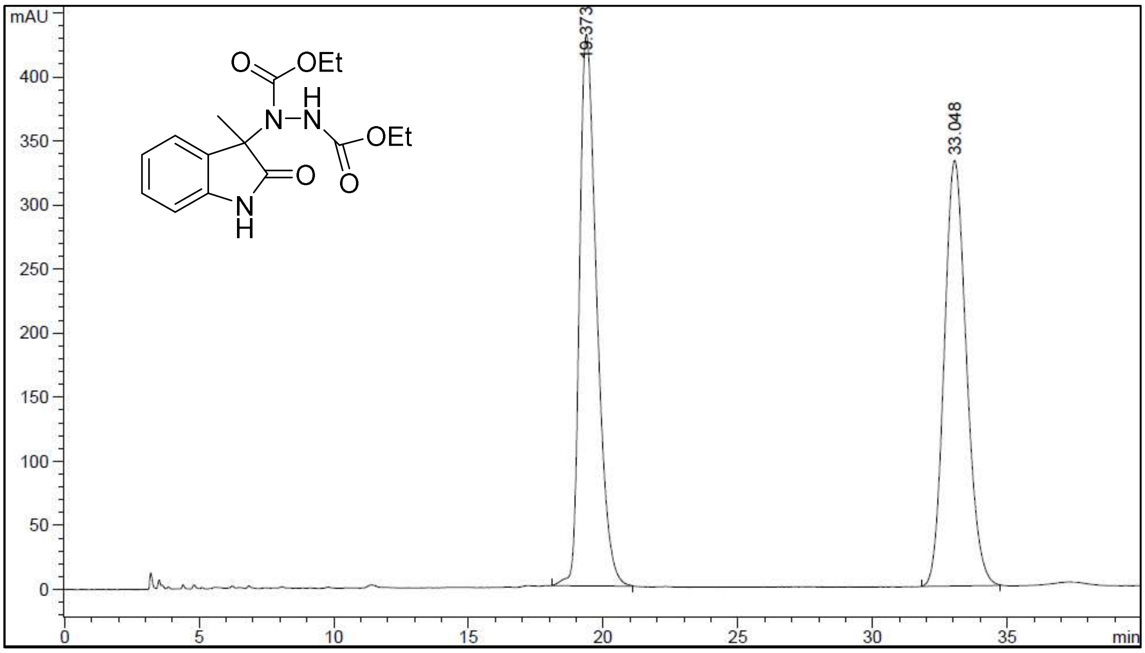

Peak	Ret. Time (min)	Type	Width	Area (mAU*s)	Height (mAU)	Area (%)
1	19.373	BB	0.6791	19237.2	430.7054	50.2589
2	33.048	BB	0.8866	19039.0	332.2327	49.7411

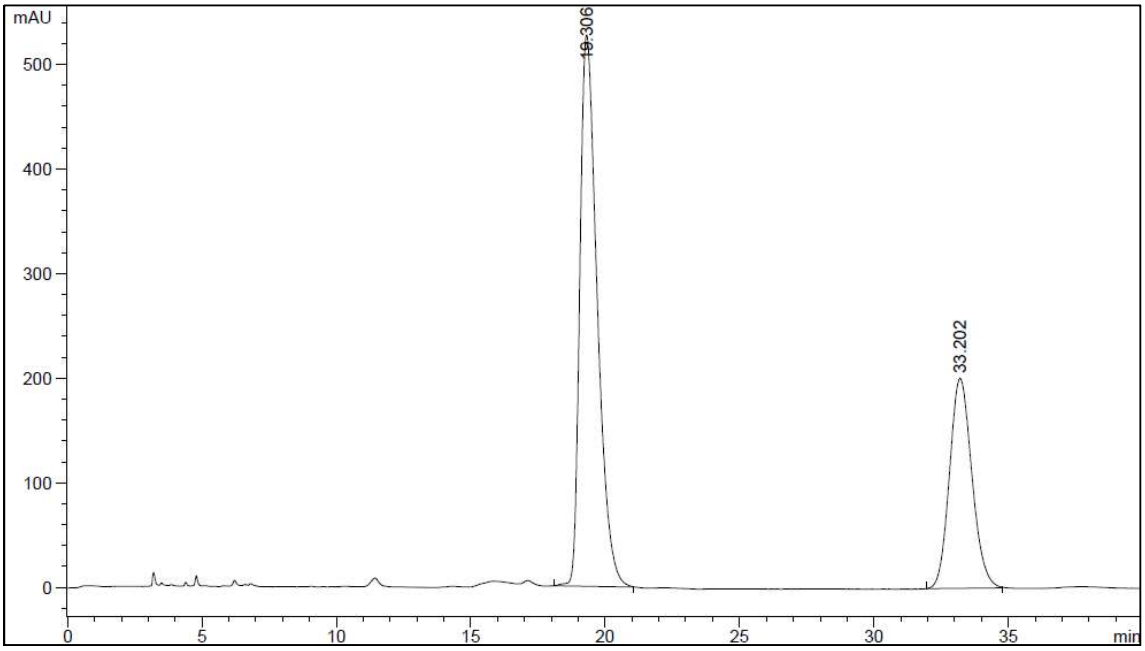

Peak	Ret. Time (min)	Type	Width	Area (mAU*s)	Height (mAU)	Area (%)
1	19.306	BB	0.6736	23657.5	527.1194	67.0371
2	33.202	BB	0.8699	11632.6	200.7762	32.9629

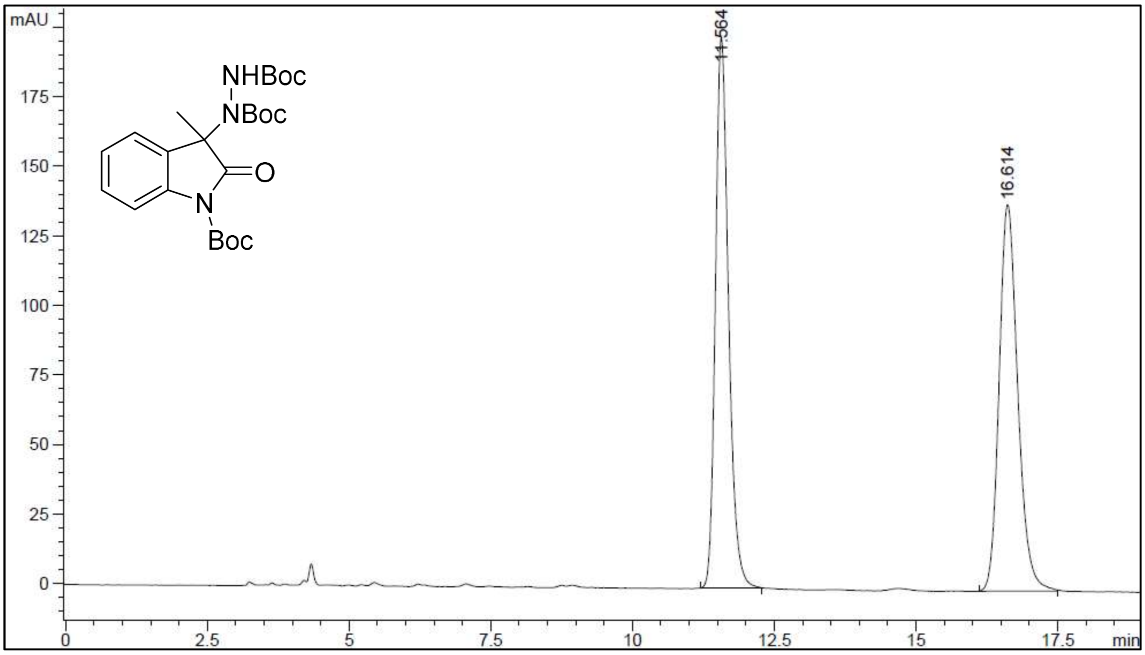

Peak	Ret. Time (min)	Type	Width	Area (mAU*s)	Height (mAU)	Area (%)
1	11.564	BB	0.2456	3188.7012	198.3303	50.0607
2	16.614	BB	0.3490	3180.9678	138.9911	49.9393

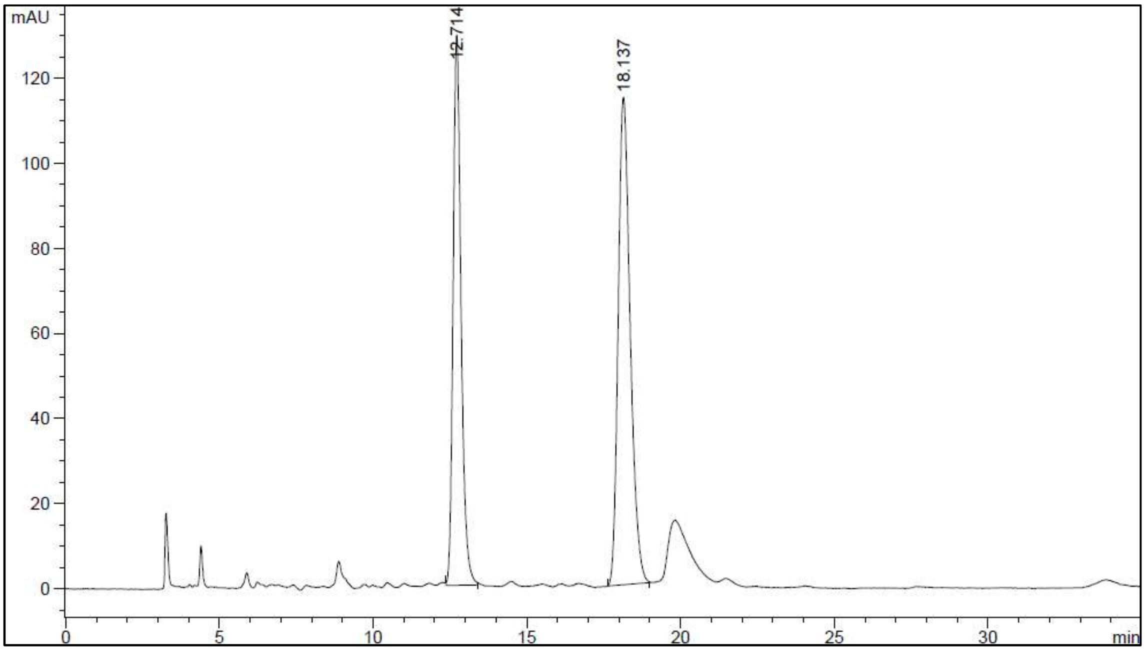

Peak	Ret. Time (min)	Type	Width	Area (mAU*s)	Height (mAU)	Area (%)
1	12.714	BB	0.2735	2323.6633	129.3830	42.7526
2	18.137	BB	0.4109	3111.4812	114.5361	57.2474

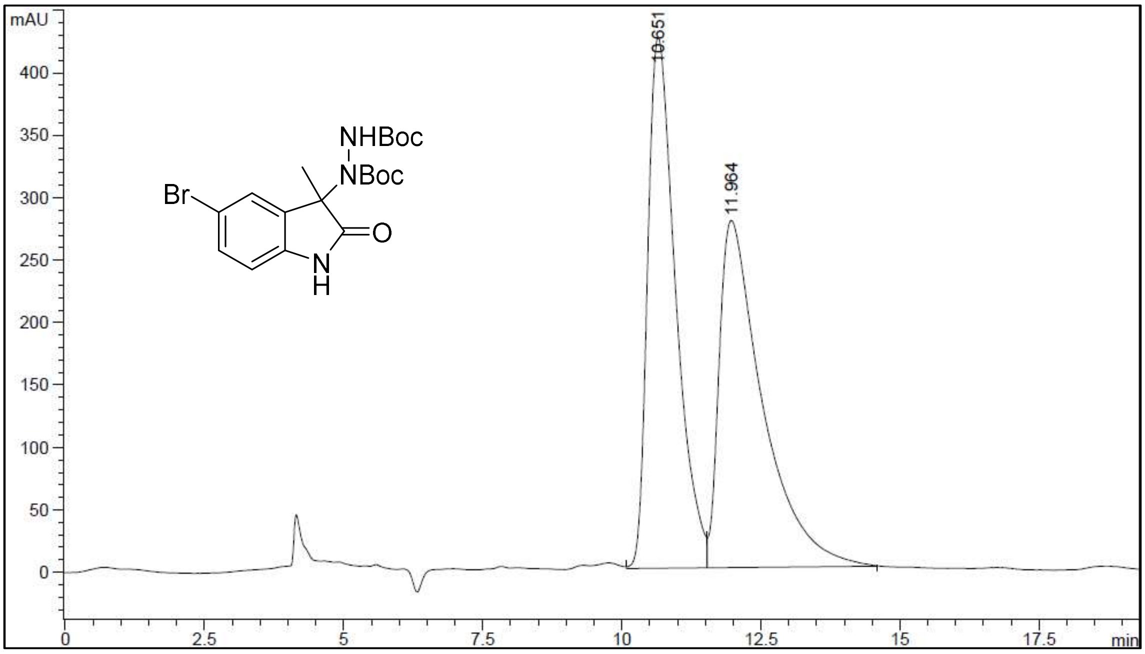

Peak	Ret. Time (min)	Type	Width	Area (mAU*s)	Height (mAU)	Area (%)
1	10.651	VV	0.5367	14974.5	425.4253	49.8070
2	11.964	VB	0.7691	15096.6	278.08582	50.1930

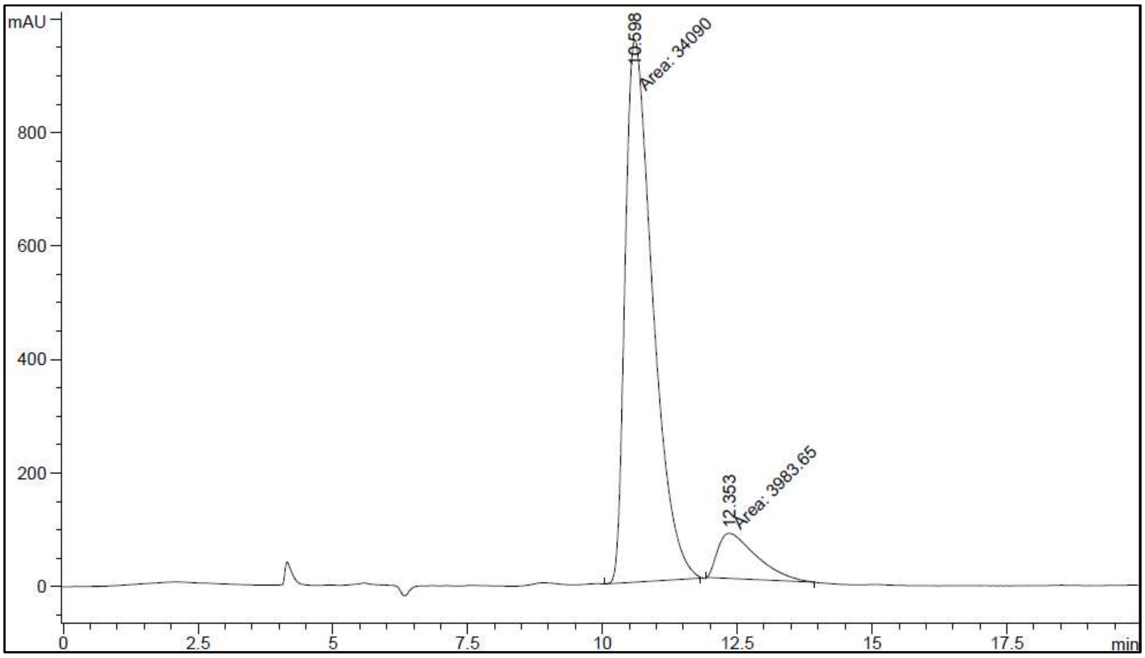

Peak	Ret. Time (min)	Type	Width	Area (mAU*s)	Height (mAU)	Area (%)
1	10.598	MM	0.5941	34090.0	956.31854	89.5370
2	12.353	MM	0.8341	3983.6533	79.60248	10.4630

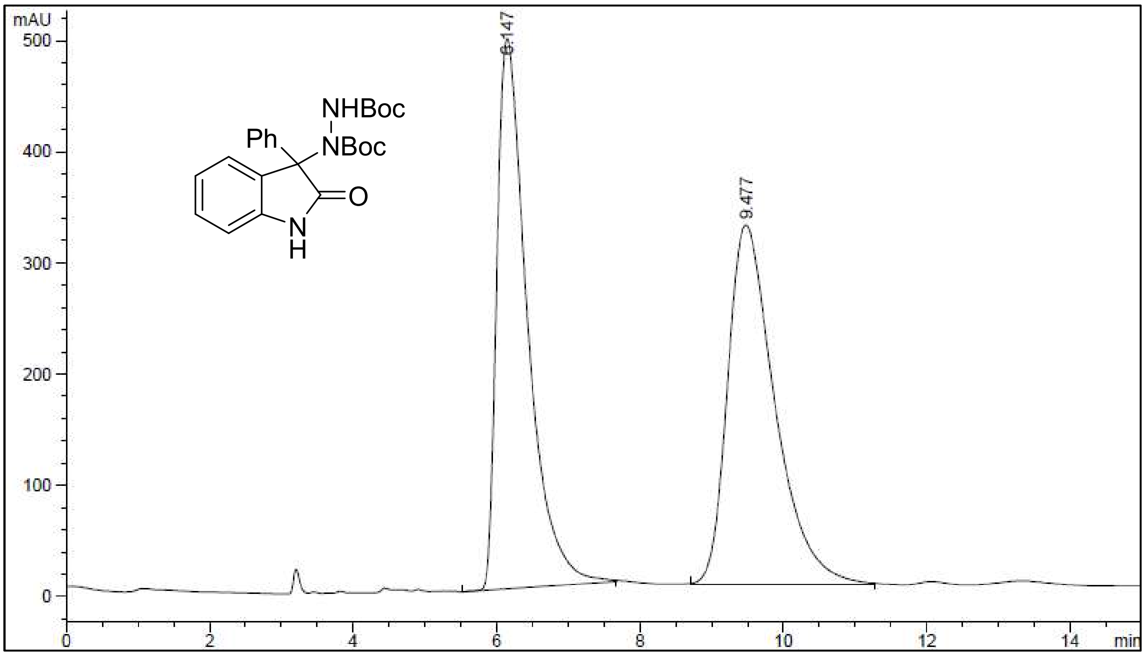

Peak	Ret. Time (min)	Type	Width	Area (mAU*s)	Height (mAU)	Area (%)
1	6.147	PB	0.4498	14661.3	494.65549	49.8696
2	9.477	BB	0.6826	14738.0	322.85226	50.1304

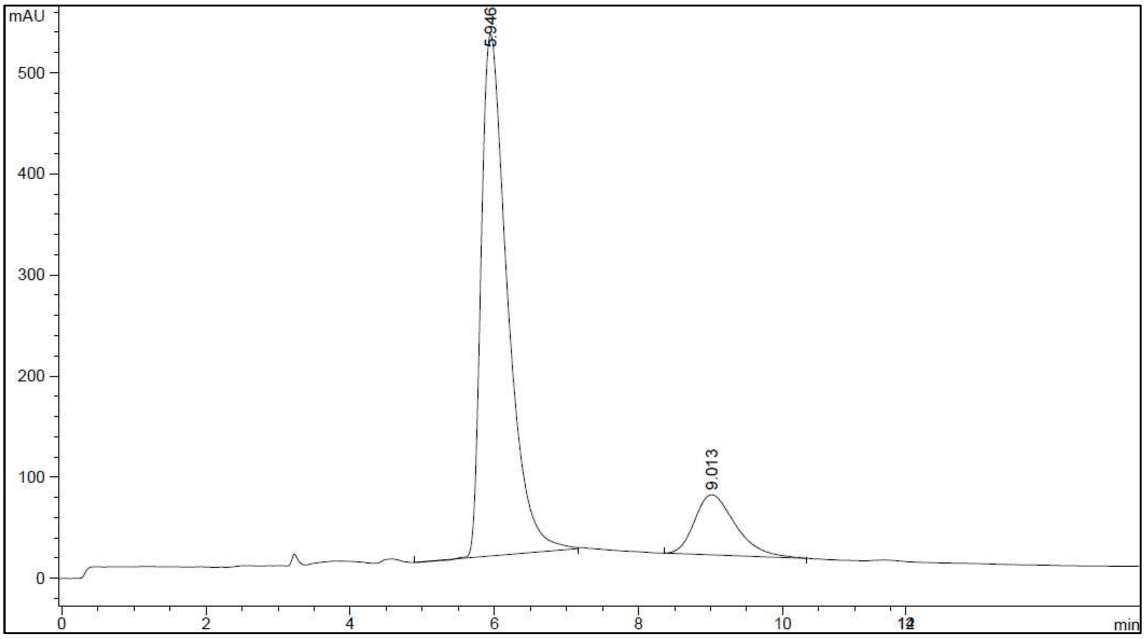

Peak	Ret. Time (min)	Type	Width	Area (mAU*s)	Height (mAU)	Area (%)
1	5.964	VB	0.3857	13082.7	516.24579	84.7728
2	9.013	BB	0.5668	2349.954	59.49723	15.2272

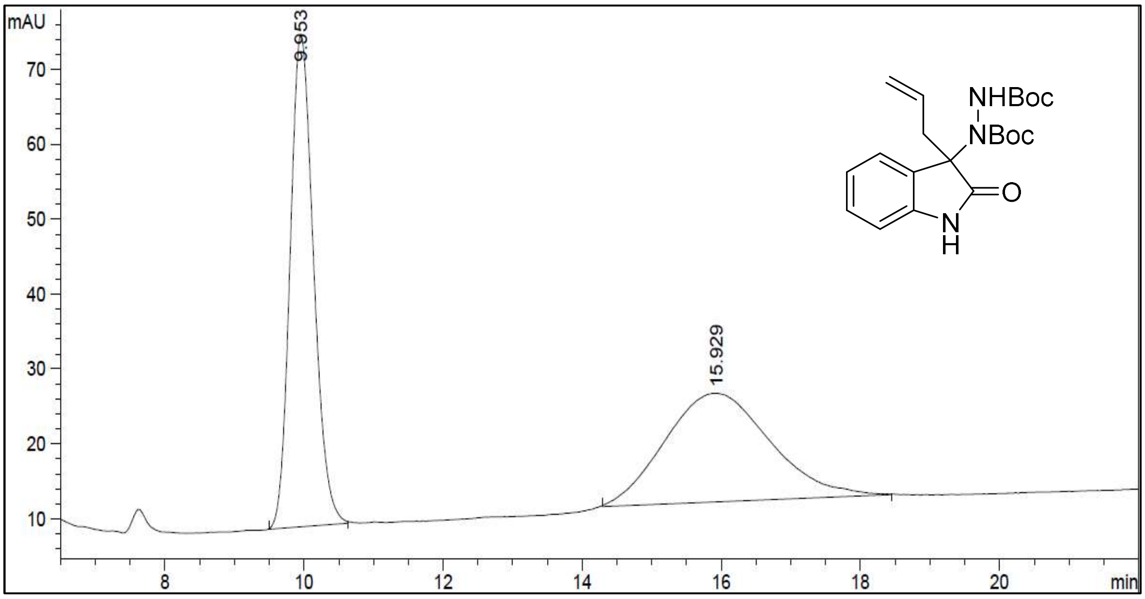

Peak	Ret. Time (min)	Type	Width	Area (mAU*s)	Height (mAU)	Area (%)
1	9.953	BB	0.3634	1540.6179	65.7352	50.1185
2	15.929	BB	1.2487	1533.3331	14.5357	49.8815

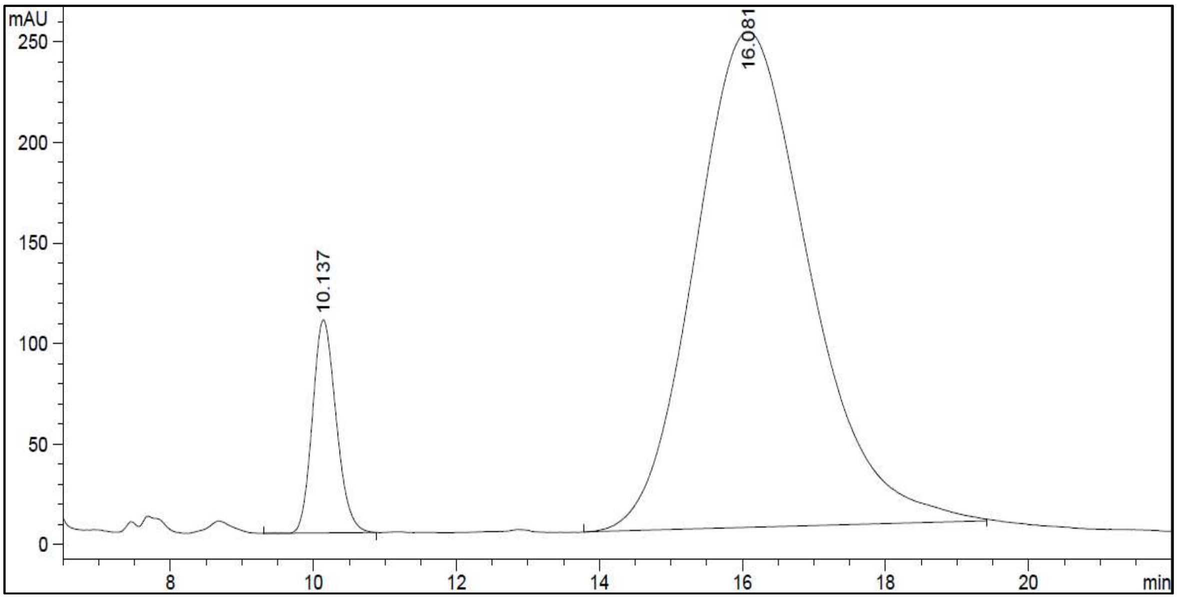

Peak	Ret. Time (min)	Type	Width	Area (mAU*s)	Height (mAU)	Area (%)
1	10.137	VP	0.3573	2448.72	706.0745	8.2257
2	16.081	PB	1.3565	27320.6	246.6083	91.7743

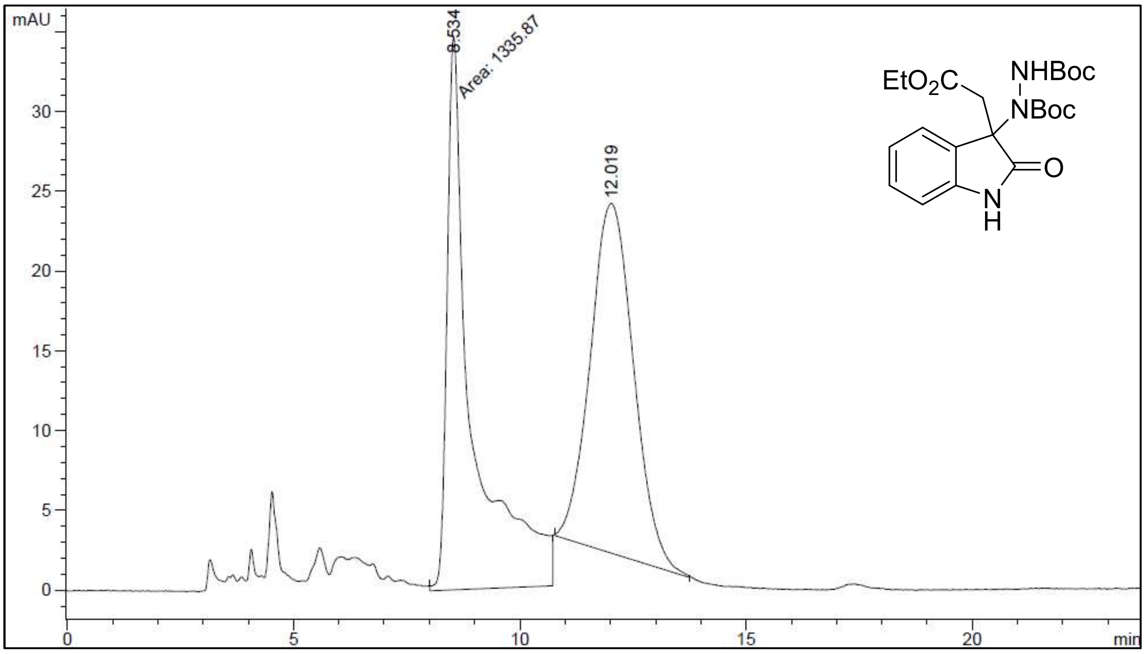

Peak	Ret. Time (min)	Type	Width	Area (mAU*s)	Height (mAU)	Area (%)
1	8.534	MM	0.6425	1335.8685	34.6539	47.7692
2	12.019	BB	0.8237	1460.6367	21.9113	52.2308

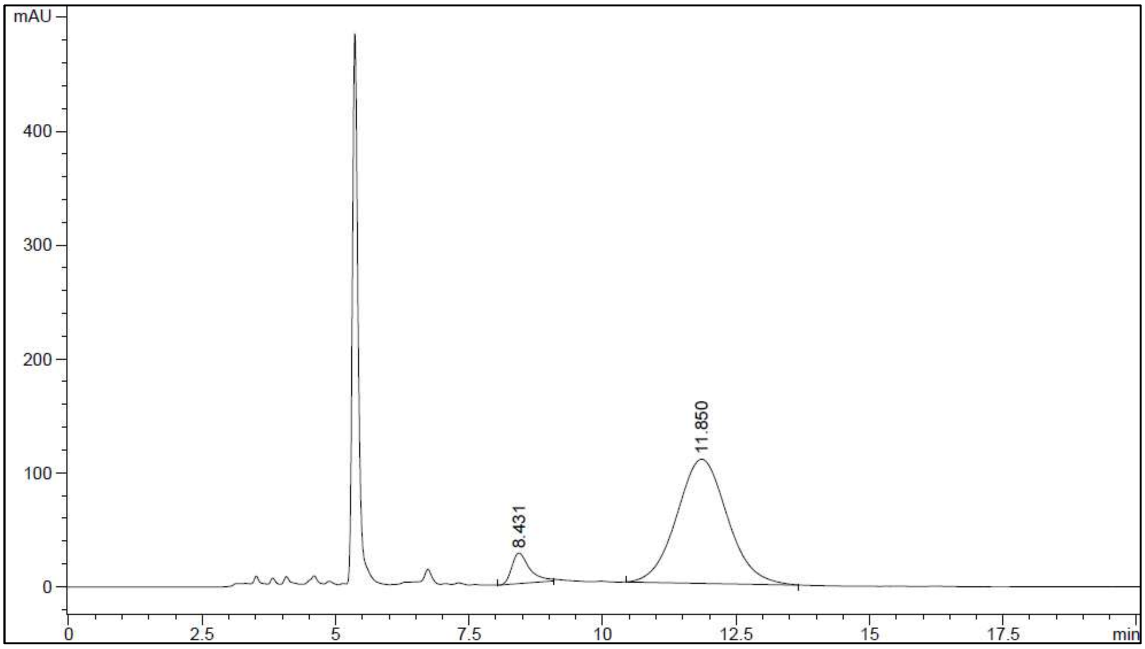

Peak	Ret. Time (min)	Type	Width	Area (mAU*s)	Height (mAU)	Area (%)
1	8.431	PB	0.3431	623.48779	26.83226	7.9315
2	11.850	BB	0.9975	7237.4639	109.1691	92.0685

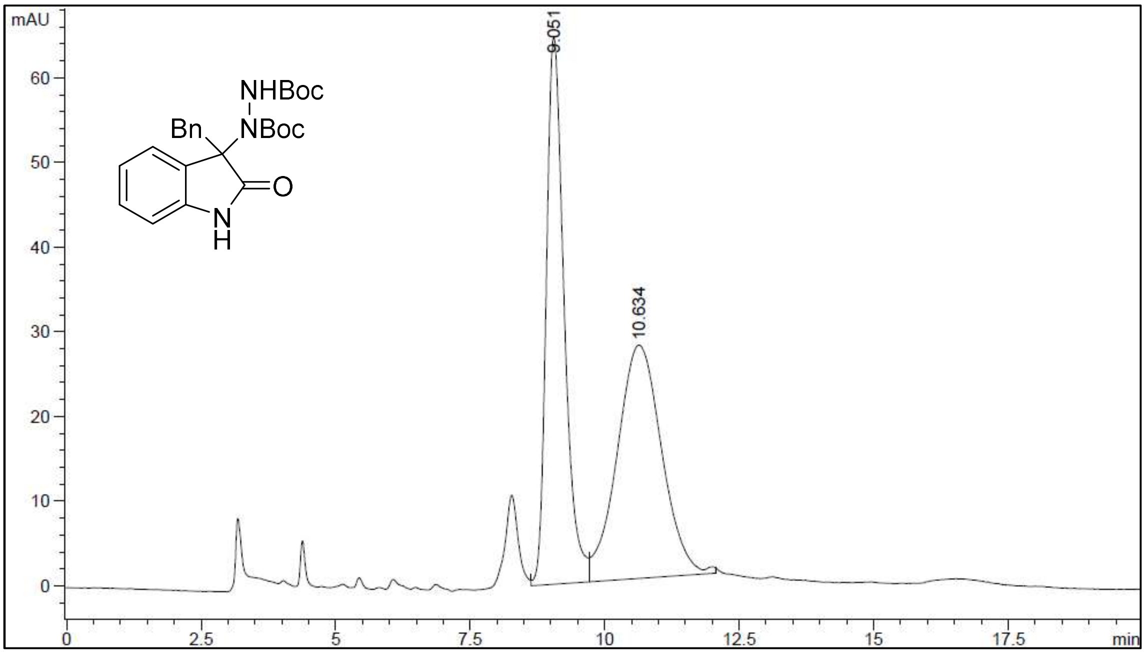

Peak	Ret. Time (min)	Type	Width	Area (mAU*s)	Height (mAU)	Area (%)
1	9.051	VB	0.3512	1492.0859	64.6549	48.3524
2	10.634	BB	0.8401	1593.7694	27.5827	51.6476

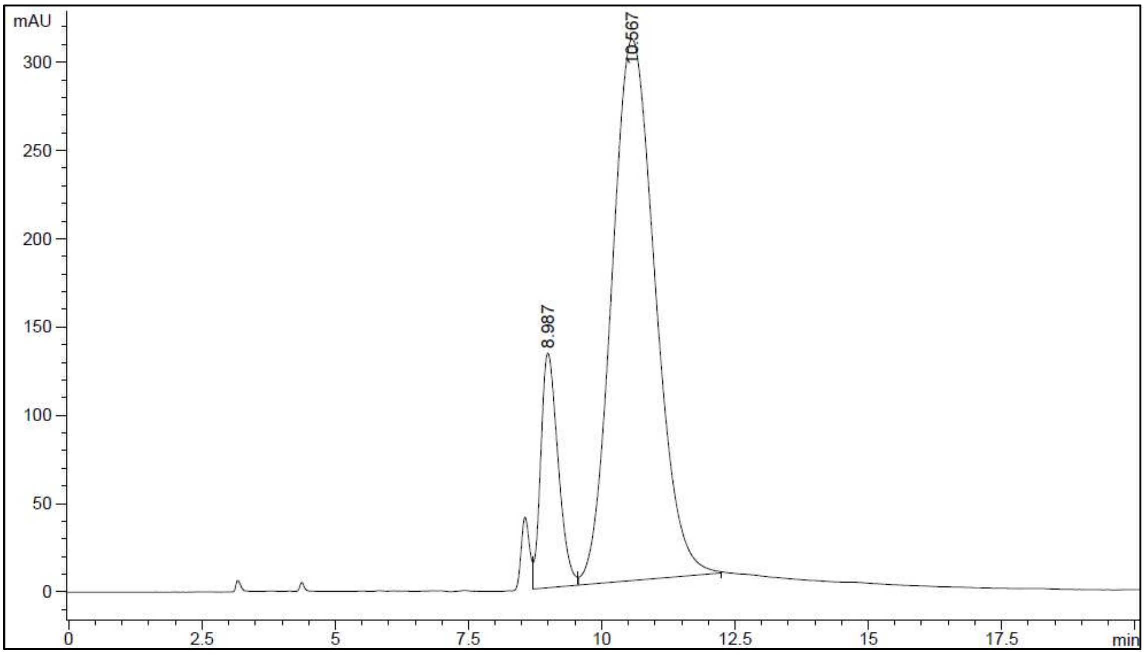

Peak	Ret. Time (min)	Type	Width	Area (mAU*s)	Height (mAU)	Area (%)
1	8.987	VV	0.3482	3008.699	132.8618	14.7821
2	10.567	VB	0.8680	17344.9	306.5994	85.2179

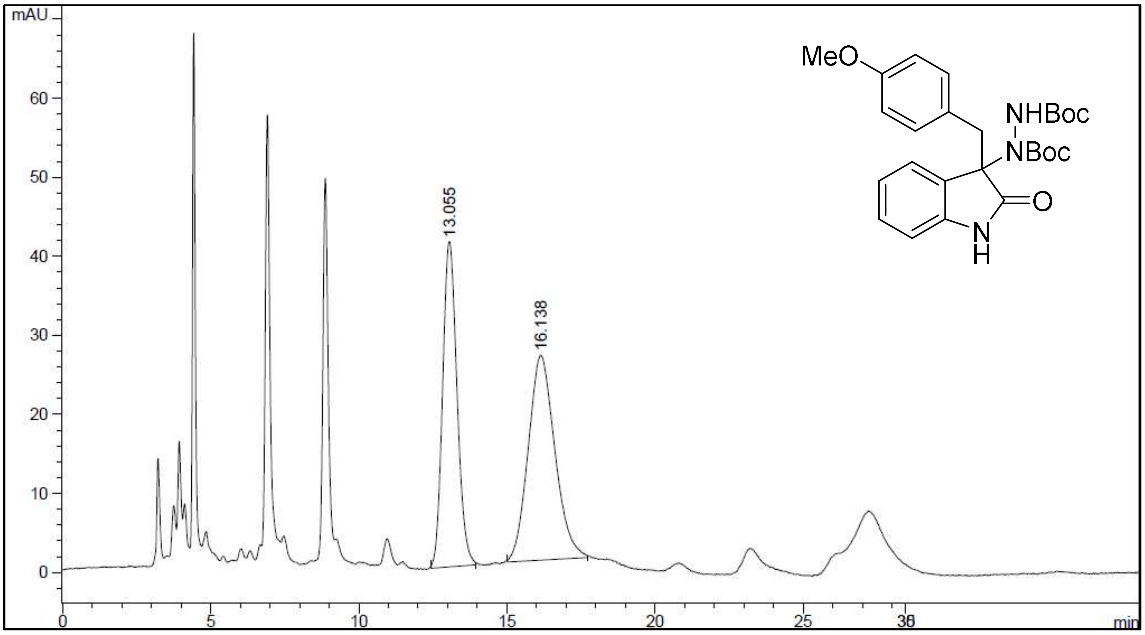

Peak	Ret. Time (min)	Type	Width	Area (mAU*s)	Height (mAU)	Area (%)
1	13.055	BB	0.5158	1389.5295	41.1617	46.1309
2	16.138	BB	0.7455	1622.6172	25.9500	53.8691

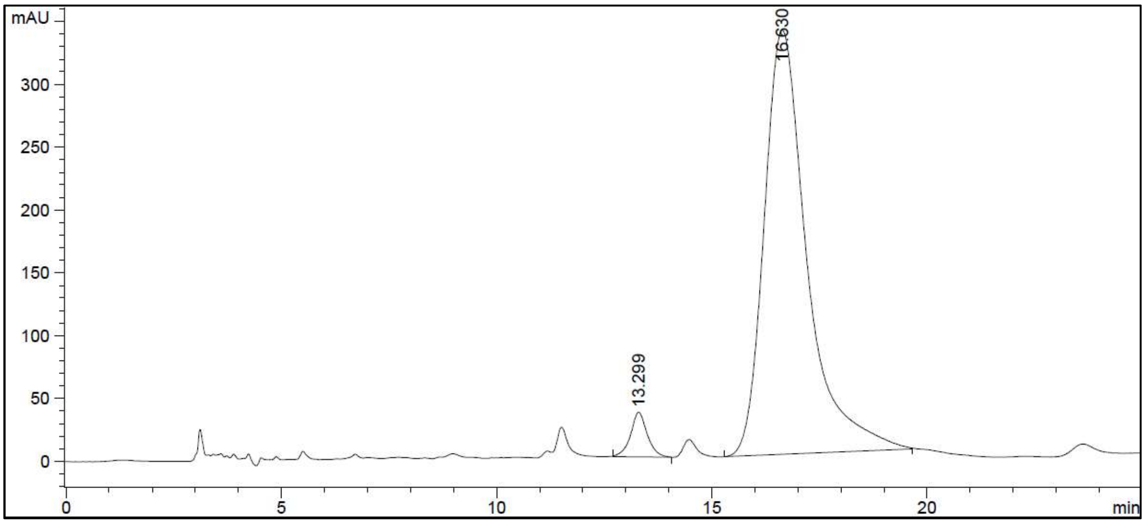

Peak	Ret. Time (min)	Type	Width	Area (mAU*s)	Height (mAU)	Area (%)
1	13.299	BP	0.3952	960.2463	35.53962	3.9522
2	16.630	PB	0.9999	23336.0	337.82153	96.0478

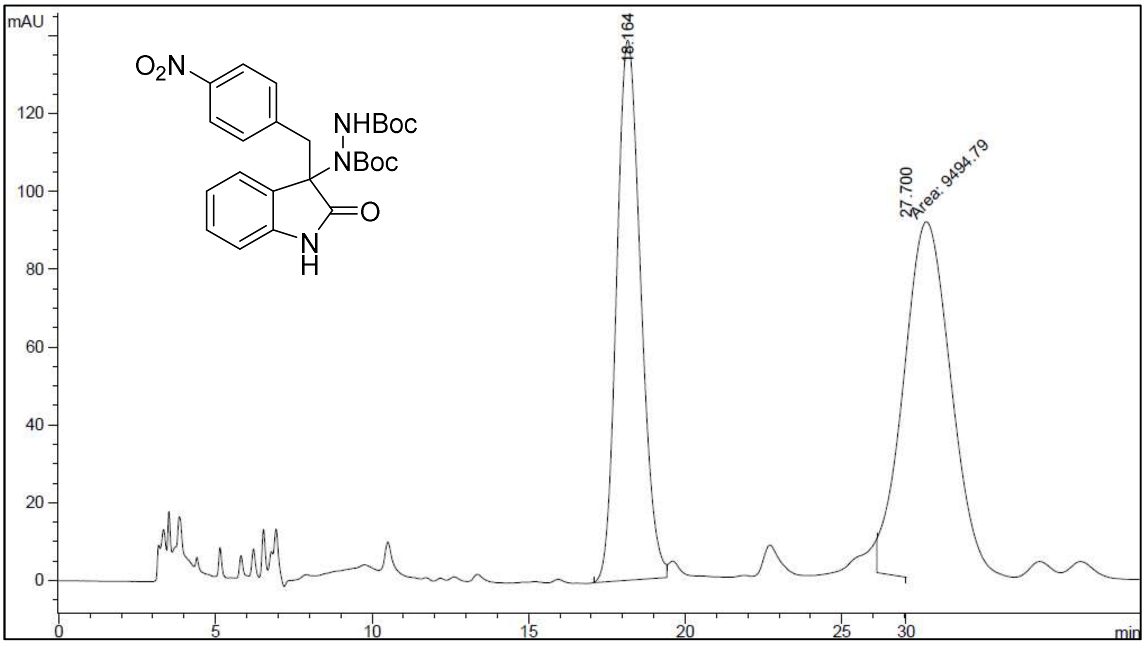

Peak	Ret. Time (min)	Type	Width	Area (mAU*s)	Height (mAU)	Area (%)
1	18.164	BV	0.8445	7428.111	138.768	43.8938
2	27.700	FN	1.7472	9494.792	90.570	56.1062

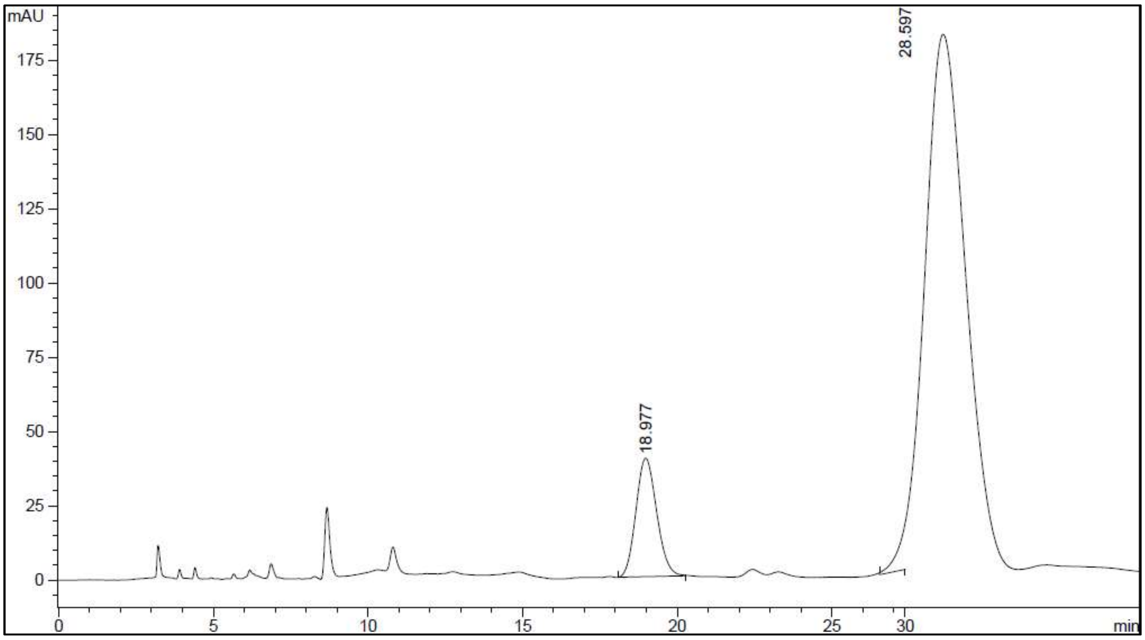

Peak	Ret. Time (min)	Type	Width	Area (mAU*s)	Height (mAU)	Area (%)
1	18.977	PB	0.7250	1863.005	39.8824	9.9558
2	28.597	BP	1.3717	16849.7	181.0661	90.0442

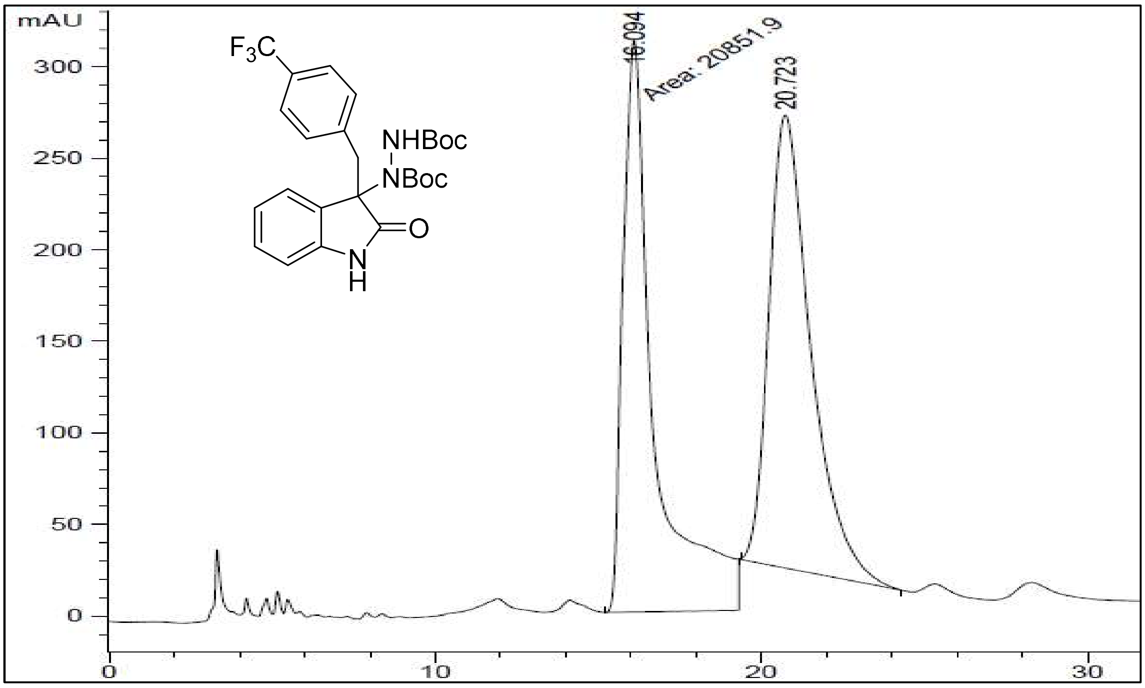

Peak	Ret. Time (min)	Type	Width	Area (mAU*s)	Height (mAU)	Area (%)
1	16.094	MM	1.1134	20851.9	312.13074	48.5496
2	20.723	BB	1.2880	22097.8	247.42282	51.4504

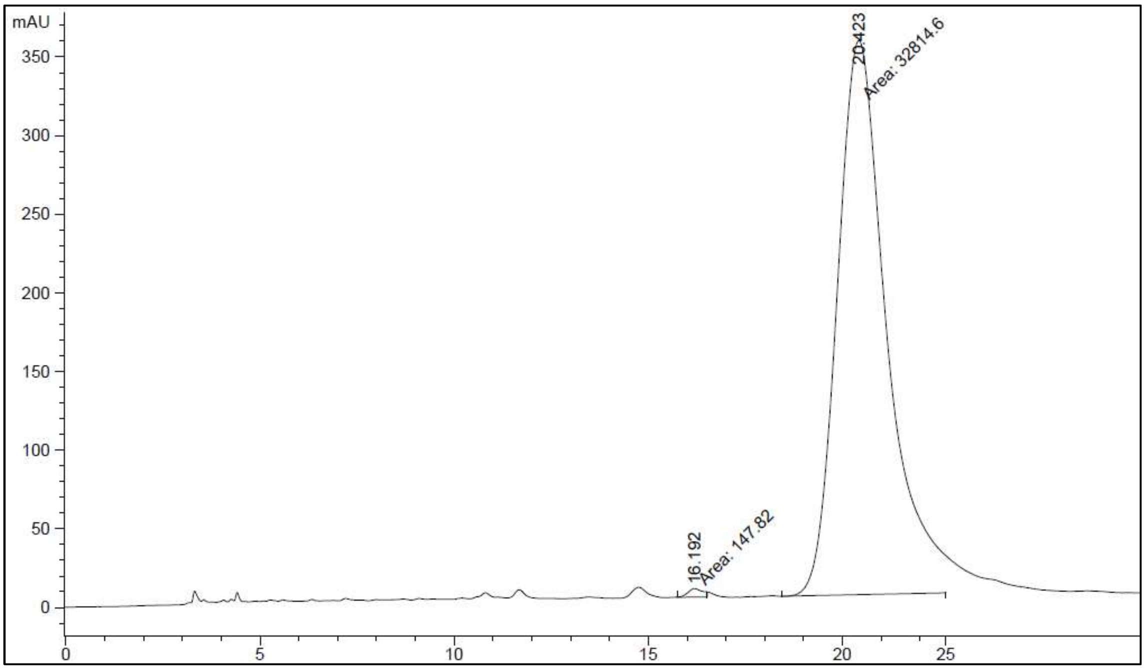

Peak	Ret. Time (min)	Type	Width	Area (mAU*s)	Height (mAU)	Area (%)
1	16.192	MF	0.4519	147.819	5.45208	0.4484
2	20.423	MM	1.5504	32814.6	352.74606	99.5516

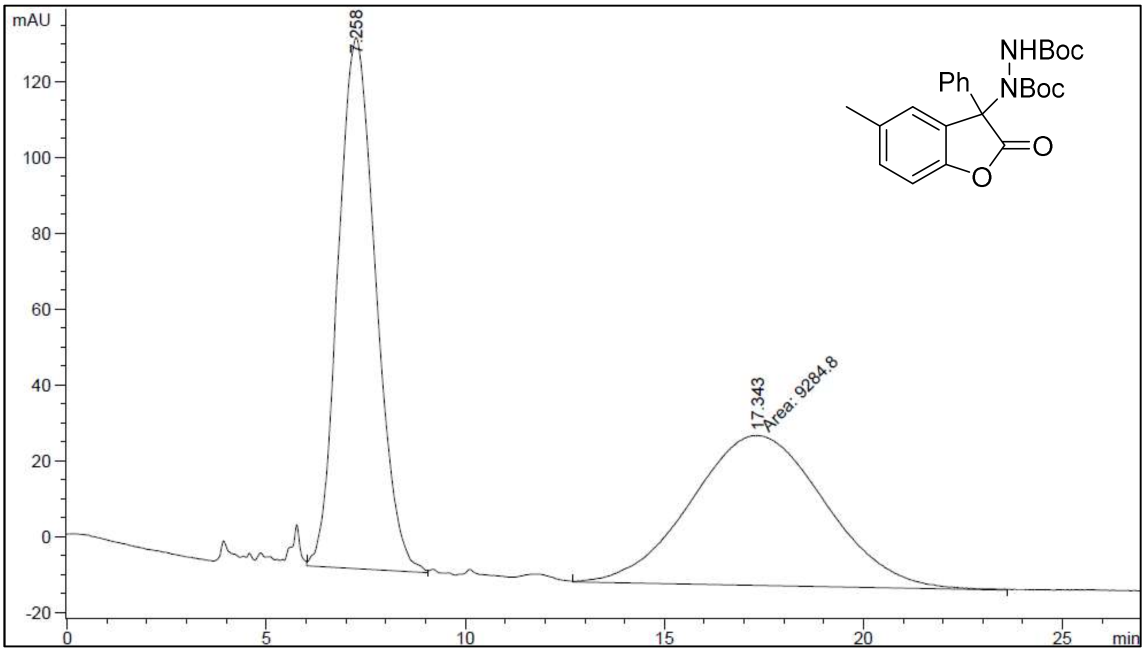

Peak	Ret. Time (min)	Type	Width	Area (mAU*s)	Height (mAU)	Area (%)
1	7.258	VV	0.9189	9241.4785	140.2176	49.8831
2	17.343	MM	3.9054	9284.8037	39.62373	50.1169

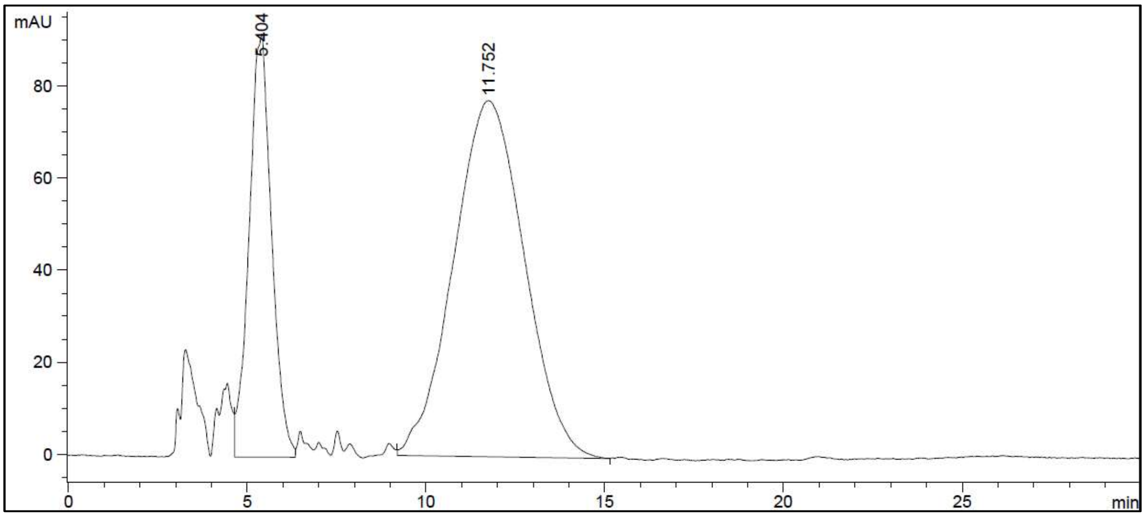

Peak	Ret. Time (min)	Type	Width	Area (mAU*s)	Height (mAU)	Area (%)
1	5.404	VV	0.5489	4027.6177	91.9744	22.4346
2	11.752	VP	1.6163	10645.2	77.3761	77.5654
